# Synthesis and Phase Behavior of a Platform of CO_2_-Soluble Functional Gradient Copolymers Bearing Metal-Complexing Units

**DOI:** 10.3390/polym14132698

**Published:** 2022-06-30

**Authors:** Andrea Ruiu, Cécile Bouilhac, Olinda Gimello, Karine Seaudeau-Pirouley, Marin Senila, Thorsten Jänisch, Patrick Lacroix-Desmazes

**Affiliations:** 1 ICGM, Univ. Montpellier, CNRS, ENSCM, 34293 Montpellier, France; andrea1.ruiu@gmail.com (A.R.); olinda.gimello@enscm.fr (O.G.); 2 Innovation Fluides Supercritiques, Batiment INEED, 26300 Alixan, France; k.seaudeau@supercriticalfluid.org; 3 INCDO INOE 2000, Research Institute for Analytical Instrumentation, ICIA, 400293 Cluj-Napoca, Romania; marin.senila@icia.ro; 4 Fraunhofer Institute for Chemical Technology, 76327 Pfinztal, Germany; tjaenisch82@gmail.com

**Keywords:** fluoropolymers, phase behavior, RAFT polymerization, supercritical carbon dioxide, complexing polymer

## Abstract

The synthesis and characterization of a platform of novel functional fluorinated gradient copolymers soluble in liquid and supercritical CO_2_ is reported. These functional copolymers are bearing different types of complexing units (pyridine, triphenylphosphine, acetylacetate, thioacetate, and thiol) which are well-known ligands for various metals. They have been prepared by reversible addition–fragmentation chain-transfer (RAFT) polymerization in order to obtain well-defined gradient copolymers. The copolymers have been characterized by proton nuclear magnetic resonance (^1^H-NMR) spectroscopy, matrix-assisted laser desorption/ionization time-of-flight (MALDI-TOF) mass spectrometry, thermal gravimetric analysis (TGA), dynamical scanning calorimetry (DSC) and cloud point measurements in dense CO_2_. All the investigated metal-complexing copolymers are soluble in dense CO_2_ under mild conditions (pressure lower than 30 MPa up to 65 °C), confirming their potential applications in processes such as metal-catalyzed reactions in dense CO_2_, metal impregnation, (e.g., preparation of supported catalysts) or metal extraction from various substrates (solid or liquid effluents). Particularly, it opens the door to greener and less energy-demanding processes for the recovery of metals from spent catalysts compared to more conventional pyro- and hydro-metallurgical methods.

## 1. Introduction

Most industrial processes involve the usage of metals [1,2,3]. These metals are usually toxic (lanthanides, cadmium) [4,5,6], precious (gold, platinum, palladium, rhodium) [7,8], represent an environmental risk (lithium, cobalt) [9,10,11,12] and their use can lead to a shortage in supply [13,14]. For these reasons, the disposal of such materials is not trivial and the industrial and urban wastes might be pre-treated to remove the metals in order to dispose of them safely or to recycle the materials. In addition, in particular in the case of precious metals, economic reasons push for the recovery of such metals, due to low resources and high economic impact on industrial processes.

Different processes are already used at an industrial scale for metal recovery. These treatments are usually performed through pyrometallurgy, hydrometallurgy, and leaching methods [15,16,17]. These approaches are highly energy-demanding, expensive, chemically dangerous, and environmentally unfavorable. As a consequence, novel methods to remove metals from industrial and urban wastes are desirable [18,19,20,21,22,23,24,25,26,27]. So far, different methods have shown interesting results: processing of metals using ionic liquids [28,29,30,31], removal of metals using polyphosphonates [32], metal recovery with ion exchange resins [33,34,35,36,37] or solvent extraction [38,39,40,41,42,43] already exhibited good extraction abilities, but in most cases, acidic aqueous solutions or waste pretreatments are necessary to reach the goal.

**Figure 1:**
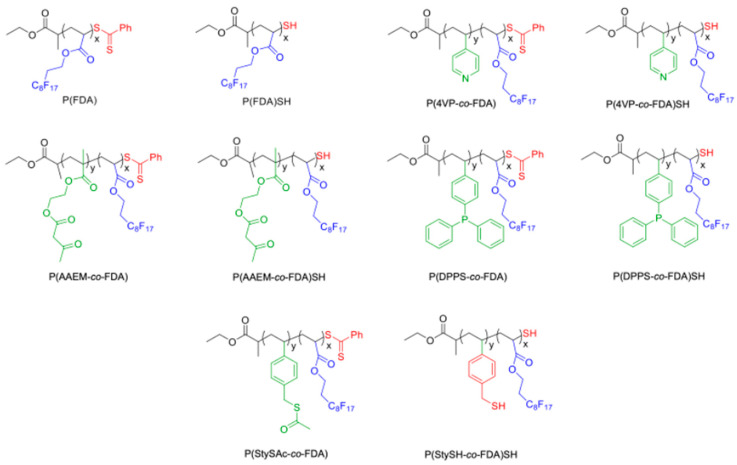
Synthesized CO_2_-soluble fluorinated homopolymers and copolymers bearing metal-complexing groups (acetoacetoxy, triphenylphosphine, pyridine, thioacetate, thiol).

A greener alternative to the aforementioned methods is the use of a different solvent system, which is dense CO_2_ (liquid CO_2_, lCO_2_, or supercritical CO_2_, scCO_2_). Above its critical point (T_c_ = 31 °C, P_c_ = 7.38 MPa), scCO_2_ has properties in between those of liquid and gas, such as high density and high diffusivity. Furthermore, dense CO_2_ is a non-polar solvent, unable to solubilize metals; thus, additives are required for metal extractions. So far, different reports have shown the use of low molecular weight compounds, (e.g., dithiocarbamates, beta-diketones, dithizone, perfluorocarboxylic acids) which can act as complexing additives [16,44,45,46,47,48,49,50,51]. Nevertheless, our group has reported in the last years the possibility to use scCO_2_-soluble metal-complexing polymers to extract metals from solid matrices, without dissolving or destroying the supports [52,53,54,55]. These polymers contain two different types of monomer units, namely CO_2_-philic monomer units ensuring a high solubility of the polymers in scCO_2_, and metal-complexing monomer units enabling binding to the metals. Even if different classes of siloxane-based [56,57] and vinyl alkanoate-based [58,59] polymers have been studied, it is known that compounds having carbon–fluorine bonds are usually soluble at much lower pressures than other compounds [60,61]. The CO_2_-philic monomer unit is represented by 1,1,2,2-tetrahydroperfluorodecylacrylate (FDA), corresponding to a fluorinated poly(1,1,2,2-tetrahydroperfluorodecylacrylate) (PFDA) homopolymer which has an extremely high affinity for dense CO_2_ [62,63,64]. The metal-complexing monomer units are usually characterized by polar groups (CO_2_-phobic) able to interact with various metals. The increasing amount of the metal-complexing monomer units has often a high impact on the phase behavior of these polymers in dense CO_2_, decreasing their solubility in this solvent. Another factor that influences the solubility of the copolymers in dense CO_2_ is the distribution of the CO_2_-phobic monomer units in the polymer chain: it is reported that gradient copolymers are soluble at a lower CO_2_ pressure than block copolymers [65]. A very straightforward method to obtain gradient copolymers is reversible addition–fragmentation chain-transfer (RAFT) copolymerization [66]. In a batch process, the gradient architecture of the copolymer is defined by the reactivity ratio of the monomers (composition drift along the copolymer chain). This reversible-deactivation radical polymerization technique allows the production of gradient copolymer in which one side of the polymer is enriched in the CO_2_-phobic metal-complexing monomer units whereas the other side of the polymer chain is enriched in CO_2_-philic units, while preserving a high solubility of the polymer in dense CO_2_ even at high loading of metal-complexing CO_2_-phobic units. Furthermore, the use of RAFT controlled radical polymerization (composition drift within the polymer chains) instead of conventional free radical polymerization (composition drift between the polymer chains) enables to have gradient polymer chains, with similar length and composition, which facilitates the copolymer solubilization in scCO_2_. In this report, we present the preparation and the phase behavior in dense CO_2_ of a platform of novel metal-complexing gradient polymers synthesized by RAFT polymerization. These polymers contain different complexing moieties (pyridine, triphenylphosphine, acetylacetate, thioacetate and thiol) which are well-known metal ligands [67,68,69,70,71,72]. The wide range of ligands introduced in the fluorinated polymer chains is usually able to complex a wide range of metals such as Li^+^, K^+^, Ag^+^, Au^+^, Pd^2+^, Gd^3+^, Hg^2+^ and Co^2+^, to name a few [69]. Taking advantage of the RAFT technique, the polymers are further modified to improve their potential complexing abilities through aminolysis reaction, transforming the dithiobenzoate moiety coming from the RAFT controlling agent into a terminal thiol group at the polymer chain end. The synthesis of these gradient polymers and the determination of their phase behavior in dense CO_2_ aims at building up a library of CO_2_-soluble metal-complexing polymers (Figure 1) as candidates to recover different types of metals from industrial and urban wastes.

## 2. Materials and Methods

### 2.1. Materials

2,2′-Azobis(2-methylpropionitrile) (AIBN, M = 164.21 g/mol, Fluka, Saint-Quentin-Fallavier, France, 98%) was purified by recrystallization in methanol and dried under vacuum before use. The chain transfer agent (CTA) ethyl-2-(phenylcarbonothioylthio)propionate (M = 254.36 g/mol, Figures S1 and S2 in SI) was synthesized and purified as previously reported in the literature [73]. 1,1,2,2-tetrahydroperfluorodecylacrylate (FDA, M = 518.17 g/mol, Boc Science, Shirley, NY, USA, >98%), α, α, α—trifluorotoluene (TFT, Aldrich, Saint-Quentin-Fallavier, France, >99%), piperidine (Aldrich, Saint-Quentin-Fallavier, France, 99%), butylamine (Aldrich, Saint-Quentin-Fallavier, France, 99.5%), triphenylphosphine (PPh_3_, Aldrich, Saint-Quentin-Fallavier, France, 99%), 4-(diphenylphosphino)styrene (DPPS, M = 288.33g/mol, Aldrich, Saint-Quentin-Fallavier, France, 97%), 4-vinylpyridine (4VP, M = 105.14 g/mol, Aldrich, Saint-Quentin-Fallavier, France, 95%), mixture of 3-vinylbenzyl chloride and 4-vinylbenzyl chloride (ratio between isomers 1/1, M = 152.62 g/mol, Aldrich, Saint-Quentin-Fallavier, France, 95%), potassium thioacetate (M = 114.21 g/mol, ACROS Organics, Illkirch, France, 98%) acetoacetoxyethyl methacrylate (AAEM, M = 214.22 g/mol, EASTMAN, Paris, France, 95%), toluene (Aldrich, Saint-Quentin-Fallavier, France, 99.5%), S-(thiobenzoyl)thioglycolic acid (Aldrich, Saint-Quentin-Fallavier, France, 99%), sodium hydroxide (NaOH, Fisher, Illkirch, France), hydrochloric acid (HCl, Aldrich, Saint-Quentin-Fallavier, France, 37%), sodium sulfate (Aldrich, Saint-Quentin-Fallavier, France, >99%), ethyl 2-mercaptopropionate (Alfa Aesar, Kandel, Germany, 98%), methanol (Aldrich, Saint-Quentin-Fallavier, France, 99%), 1,1,2-trichlorotrifluoroethane (CFC-113, Freon 113, Aldrich, Saint-Quentin-Fallavier, France, 99%), carbon dioxide (CO_2_, SFE 5.2, Air Liquide, Paris, France, 99.9%), were used as received unless otherwise indicated.

### 2.2. Monomer and Polymer Syntheses

#### 2.2.1. Synthesis of S-(Vinylbenzyl) Ethanethioate (StySAc) Monomer

Synthesis of StySAc is illustrated in Figure S3 in the SI. Vinylbenzyl chloride (20 g, 0.131 mol) and 50 mL of acetone were added to a round bottom flask. The mixture was stirred with a magnetic stir bar for 20 min in an ice bath under N_2_ atmosphere. Afterward, potassium thioacetate (1.2 eq., 18 g, 0.157 mol) was added over 30 min and the reaction stirred for 30 min in an ice bath and then 48 h at room temperature. Then, the mixture was filtered on a filter paper and the acetone evaporated under reduced pressure. The obtained yellow oil was solubilized in CH_2_Cl_2_ (40 mL) and washed with deionized water (3 × 50 mL). Finally, the organic phase was dried with Na_2_SO_4_ and the solvent evaporated under reduced pressure. An amount of 21.4 g of final compound was collected as a brown oil (85% yield). The structure of the product, S-(Vinylbenzyl) ethanethioate (M = 192.28 g/mol), was confirmed by ^1^H-NMR analysis, as shown in Figure S4.

#### 2.2.2. Synthesis of Poly(1,1,2,2-tetrahydroperfluorodecylacrylate) (P(FDA)) Homopolymer

FDA (40 g, 0.0771 mol), CTA (2.141 g, 0.00842 mol), AIBN (0.415 g, 0.00253 mol) and TFT (42 mL) were added in a Schlenk flask. The mixture was stirred magnetically and bubbled for 40 min with N_2_. Then, the polymerization was started by heating the Schlenk flask in an oil bath at 65 °C. At the end of the reaction, the mixture was let return to room temperature and it was precipitated in 600 mL of pentane 3 times from TFT solution and the polymer was dried under vacuum overnight at room temperature. After drying, 32.8 g of polymer were recovered as a fine pink powder (78% yield).

#### 2.2.3. Synthesis of Poly(1,1,2,2-tetrahydroperfluorodecylacrylate-*co*-4-vinylpyridine) (P(4VP-*co*-FDA)) Copolymer

General procedure for copolymerization: FDA (30.6 g, 0.0590 mol), 4VP (5.40 g, 0.0513 mol), CTA (0.936 g, 0.00368 mol), AIBN (0.180 g, 0.00110 mol) and toluene (36 mL) (see SI Section S2) were added in a Schlenk flask. The mixture was stirred magnetically and bubbled for 40 min with N_2_. Then, the polymerization was initiated by heating the Schlenk flask in an oil bath at 65 °C. After 120 h, the reaction was let to return to room temperature, and it was precipitated in 600 mL of pentane 3 times from toluene solution and the polymer was dried under vacuum overnight at room temperature. After drying, 21.7 g of polymer were recovered as a fine pink powder (59% yield).

#### 2.2.4. Synthesis of Poly(1,1,2,2-tetrahydroperfluorodecylacrylate-*co*-acetoacetoxyethyl Methacrylate) (P(AAEM-*co*-FDA)) Copolymer

The general copolymerization procedure was applied with the following conditions: FDA (42.0 g, 0.0810 mol), AAEM (18.0 g, 0.0840 mol), CTA (1.566 g, 0.00616 mol), AIBN (0.302 g, 0.00184 mol) and TFT (64.6 mL). Reaction time: 96 h. After drying, 26.7 g of polymer were recovered as a fine pink powder (43% yield).

#### 2.2.5. Synthesis of poly(1,1,2,2-tetrahydroperfluorodecylacrylate-*co*-4-(diphenylphosphino)styrene) (P(DPPS-*co*-FDA)) Copolymer

The general copolymerization procedure was applied with the following conditions: FDA (42.5 g, 0.0820 mol), DPPS (7.50 g, 0.0260 mol), CTA (1.305 g, 0.00513 mol), AIBN (0.252g, 0.00153 mol) and TFT (54 mL). Reaction time: 96 h. After drying, 31.1 g of polymer were recovered as a fine pink powder (61% yield).

#### 2.2.6. Synthesis of Poly(1,1,2,2-tetrahydroperfluorodecylacrylate-*co*-S-(Vinylbenzyl) Ethanethioate) (P(StySAc-*co*-FDA)) Copolymer

The general copolymerization procedure was applied with the following conditions: FDA (42.5 g, 0.0820 mol), StySAc (7.50 g, 0.0390 mol), CTA (1.305 g, 0.00513 mol), AIBN (0.252 g, 0.00153 mol) and TFT (54 mL). Reaction time: 96 h. After drying, 28.5 g of polymer were recovered as a fine pink powder (55% yield).

### 2.3. Aminolysis of the Polymers

As a general procedure for aminolysis of the polymers, polymer (2 g), PPh_3_ (5 molar eq. versus aminolyzable group), and TFT (5 mL) were added in a Schlenk flask. The mixture was stirred magnetically and bubbled for 40 min with N_2_. Afterward, piperidine (3 molar eq. versus polymer) was added. The mixture was stirred for 3 h at room temperature. Then, the reaction mixture was precipitated in 60 mL of pentane 3 times from TFT solution and the polymer was dried under vacuum overnight at room temperature.

### 2.4. Characterizations

#### 2.4.1. Cloud Point Measurements

Cloud point measurements were carried out in a high pressure, variable volume view cell equipped with a sapphire window on the end for visual observations. The cell was equipped with a pressure transducer and an internal thermocouple. It was thermostated by a water/isopropanol alcohol mixture delivered by a Lauda RE206 circulating pump. CO_2_ is delivered by an ISCO 260D automatic syringe pump. An amount of 55 mg of polymer was weighed and transferred to the cell along with a clean stirring bar at a starting volume of 6.40 mL. Subsequently, the cell was fed with CO_2_ at about 25 °C and 10.9 MPa. Then, the cell was heated to 65 °C (taking care to adjust the volume of the cell in order to stay below a pressure of 35 MPa; safety rupture disk at 50 MPa). Cloud points (one-phase/two-phase transition) were obtained by decreasing the pressure of the cell by increasing the cell volume through a hand-driven piston after 15 min of stirring at a given temperature. The cell was cooled by steps of 5 °C down to 25 °C. The uncertainty of the cloud point pressure was ±0.5 MPa.

#### 2.4.2. Nuclear Magnetic Resonance Spectroscopy (NMR)

Chemical structures were determined by ^1^H-NMR spectroscopy on a Bruker Avance 400 MHz spectrometer at room temperature. The spectra were recorded by dissolving 10 mg of sample in 0.5 mL of CDCl_3_ (StySAc and 4VP-based copolymers), acetone-d_6_ (AAEM copolymers), CFC-113 with C_6_D_6_ capillaries (other fluorinated (co)polymers).

#### 2.4.3. Thermogravimetric Analysis (TGA)

TGA measurements were performed by placing 10–15 mg samples in an aluminum pan on a Q50—TA Instruments, heating the sample up to 580 °C at 10 °C min^−1^ under N_2_ inert atmosphere.

#### 2.4.4. Differential Scanning Calorimetry (DSC)

DSC measurements were performed by placing 10–15 mg samples in an aluminum crucible on a Netzsch DSC 200 F3 instrument using three heating–cooling cycles from −40 to 150 °C, with 20 °C/min heating rate under N_2_ inert atmosphere. Calibration of the instrument was performed with noble metals and checked with an indium sample.

#### 2.4.5. MALDI-TOF-MS Mass Spectrometry

MALDI-TOF mass spectra were performed at the Laboratoire de Mesures Physiques of Montpellier University using a Bruker Rapiflex time-of-flight mass spectrometer and a nitrogen laser for MALDI (λ = 337 nm). The measurements in reflectron positive ion mode were recorded with a voltage and reflector lens potential of 20 kV and 21.5 KV, respectively. Mixtures of peptides were used for external calibration.

The matrix and cationizing agent were trans-2-[3-(4-tert-butylphenyl)-2-methyl-2-propenylidene]malononitrile (DCTB) diluted at 20 mg/mL in dichloromethane and sodium trifluoroacetate (Na^+^TFA^−^) diluted at 5 mg/mL in acetone for P(FDA) homopolymer, and P(AAEM-*co*-FDA), P(StySAc-*co*-FDA) and P(4VP-*co*-FDA) gradient copolymers. The polymer concentration was 20 mg/mL in trifluorotoluene (C_6_H_5_CF_3_). The polymer and matrix were mixed in a 1:1 volume ratio. The Na^+^TFA^−^ layer was first deposited on the target and dried. After evaporation of solvent, the mixture, composed of polymer and matrix, was placed on the top of the sodium trifluoroacetate layer in the MALDI target.

Regarding the P(DPPS-*co*-FDA) gradient copolymer, the matrix was dithranol diluted at 20 mg/mL in dichloromethane and the cationizing agent was NaI diluted at 5 mg/mL in acetone. The polymer concentration was 10 mg/mL in hexafluoro-isopropanol (HFIP). The polymer and matrix were mixed in a 1:5 volume ratio. The NaI layer was first deposited on the target and dried. After evaporation of solvent, the mixture, composed of polymer and matrix, was placed on the top of the NaI layer in the MALDI target.

## 3. Results and Discussion

### 3.1. Synthesis and Characterization of the (Co)Polymers before Aminolysis

The synthesis of the proposed (co)polymers (Figure 1) has been achieved through the reversible-deactivation radical polymerization technique, specifically reversible addition–fragmentation chain-transfer (RAFT) polymerization [66]. RAFT polymerization is compatible with many functional groups. This technique allows preparing gradient copolymers, thanks to the reactivity ratio of the different monomers. Indeed, the Alfrey and Price Q and e values of FDA, 4VP and AAEM are available in the literature: Q_FDA_ = 0.44 and e_FDA_ = 0.45 [74], Q_4VP_ = 2.47 and e_4VP_ = 0.84, [75], Q_AAEM_ = 0.68 and e_AAEM_ = 0.13 [76]. The Q and e values of chloromethyl styrene (CMSty) have been considered for DPPS and StySAc as a first approximation: Q_CMSty_ = 1.13 and e_CMSty_ = −0.58 [77]. From these Q and e values, the reactivity ratios could be estimated by using the classical Alfrey and Price equation [75]: r_4VP_ = 4.05 and r_FDA_ = 0.21, r_AAEM_ = 1.61 and r_FDA_ = 0.56, r_CMSty_ = 1.41 and r_FDA_ = 0.24. Thus, the complexing monomers are preferably introduced during copolymerization with FDA, (i.e., polymer chains are first enriched with complexing units), leading to gradient copolymer structures. In addition, we observed by ^1^H-NMR of the crude reaction medium at a given time that the complexing monomers (4VP, AAEM, DPPS, StySAc) polymerize faster than FDA, which confirmed the theoretical data (Figures S5–S7). Thus, the beginning of the polymer chain is enriched in the complexing monomer units whereas the end of the polymer chain is enriched in the FDA CO_2_-philic monomer units (gradient structure).

The targeted molecular weight (M_n,targeted_) (Equation (S1)) was set to 5000 g/mol for P(FDA) and 10,000 g/mol for the other copolymers. The AIBN/CTA molar ratio was set to 0.3 in all cases and polymerizations were performed at 65 °C in TFT or toluene in the case of P(4VP-*co*-FDA). The associated results are gathered in Table 1.

P(FDA) with a molecular weight of around 5000 g/mol and four copolymers with a molecular weight of ca. 10,000 g/mol were successfully obtained. All the polymers were purified by precipitation in pentane. P(FDA) homopolymer and the respective copolymers with DPPS and 4VP were obtained as a fine red powder. Instead, the fluorinated copolymers containing AAEM and StySAc units were isolated as a pink sticky gum after precipitation. After drying under a vacuum, they become fragile foams that can be easily milled into powder. High monomer conversions were reached (from 80 to 100% conversion) and quite good correlations are observed between M_n,targeted_ and M_n,NMR_. The higher values obtained for M_n,NMR_ may be explained by (co)polymer chain fractionation during the precipitation step, causing the loss of lower molecular weight chains.

**Figure 2:**
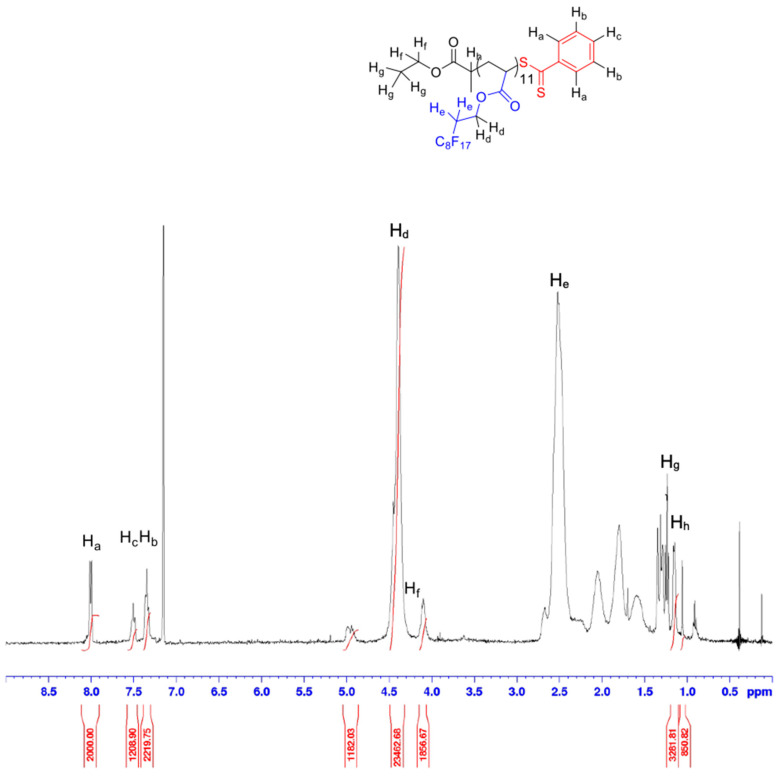
^1^H-NMR spectrum (400 MHz, CFC-113 + C_6_D_6_ capillaries) of P(FDA) after precipitation.

^1^H-NMR analyses were performed to determine polymer chain lengths, as well as the ratio between complexing and CO_2_-philic monomer units (SI Section S2). These analyses have been performed using acetone-d_6_ for AAEM copolymer [78], CDCl_3_ for 4VP and StySAc copolymers, and CFC-113 as a solvent with C_6_D_6_ capillaries (for locking) for the other (co)polymers. The selection of this unusual solvent was due to the different solubility of the polymers in common solvents: the fluorinated copolymers with AAEM and StySAc units were also soluble in acetone and ethyl acetate, while the copolymer with 4VP is soluble in chloroform; contrarily, the other FDA homo- and copolymers were soluble exclusively in fluorinated solvents such as TFT or CFC-113. To calculate the polymer composition, the aromatic signal of the RAFT agent has been used as a reference. ^1^H-NMR of the P(FDA) homopolymer permits to easily identify the typical signal of the fluorinated units once polymerized, as well as the CTA signals (Figure 2). This information is useful for the more complicated copolymer NMR analyses. The signal of the aromatic protons of the CTA is visible between δ = 7.5 and 8.3 ppm. The signal at δ = 5 ppm corresponds to the proton of the last FDA monomer unit, in α-position to the thioester group of the CTA chain end. Another typical signal is present at δ = 4.5 ppm, which is assigned to the methylene protons of the fluorinated alkyl group and is used to calculate the number of FDA monomer units in the polymer.

To calculate the mean number and average degree of polymerization DP_FDA_ and the molecular weight M_n,precipitated,P(FDA)_ for the P(FDA), Equations (S4) and (S5) were used. M_n,precipitated,P(FDA)_ equal to 5850 g/mol and DP_FDA_ of 10.8 were estimated by ^1^H-NMR.

**Figure 3:**
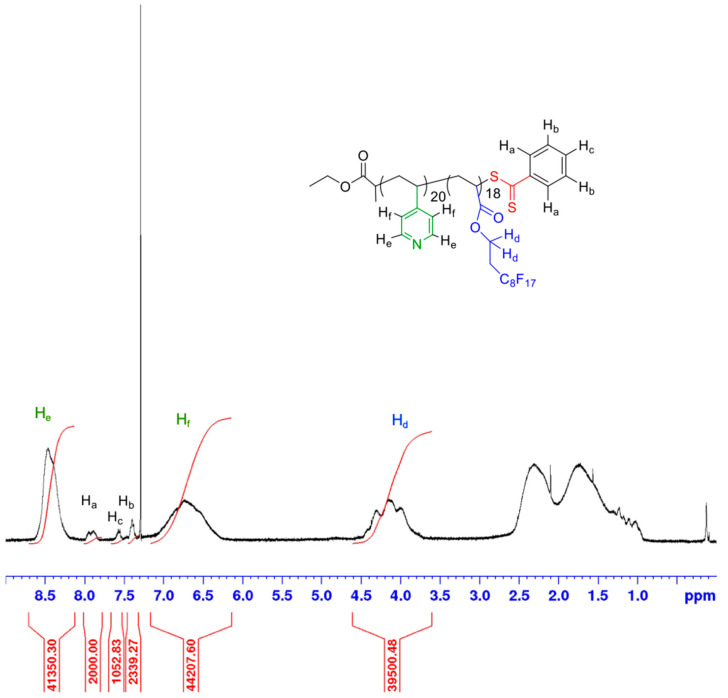
^1^H-NMR spectrum (400 MHz, CDCl_3_) of P(4VP-*co*-FDA) after precipitation.

^1^H-NMR spectrum of P(4VP-*co*-FDA) showed the appearance of two broad peaks at δ = 6.5 and 8.5 ppm, in addition to the typical FDA signal at δ = 4 ppm (Figure 3). These signals are assigned to the pyridinic protons.

As for the P(FDA) homopolymer, Equations (S8)–(S10) can be used to determine the degrees of polymerization and the molecular weight of the copolymer. A copolymer with a molecular weight of 11,820 g/mol and degrees of polymerization of 19.8 for the 4VP complexing monomer and 18.3 for FDA was obtained.

**Figure 4:**
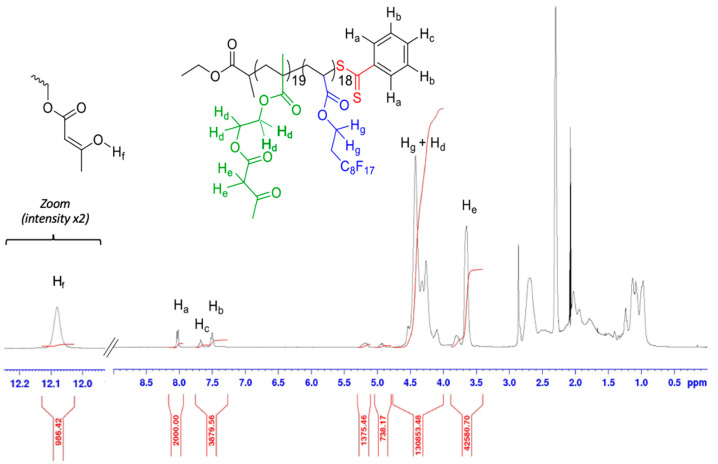
^1^H-NMR spectrum (400 MHz, acetone-d_6_) of P(AAEM-*co*-FDA) after precipitation.

The ^1^H-NMR spectrum obtained after FDA and AAEM copolymerization and recorded using acetone-d_6_ as a solvent, shows two different forms of the AAEM moiety, as ketone and as enol, with 4.5 mol% of the enolic conformation (Figure 4).

The calculation of the degrees of polymerization and polymer molecular weight has been performed by taking into account both ketone and enol forms of the AAEM monomer units, using Equations (S13)–(S15). We obtained a molecular weight equal to 13,480 g/mol with DP_AAEM_ = 18.9 and DP_FDA_ = 17.7 for the copolymer P(AAEM-*co*-FDA) according to ^1^H-NMR spectrum (Figure 4).

**Figure 5:**
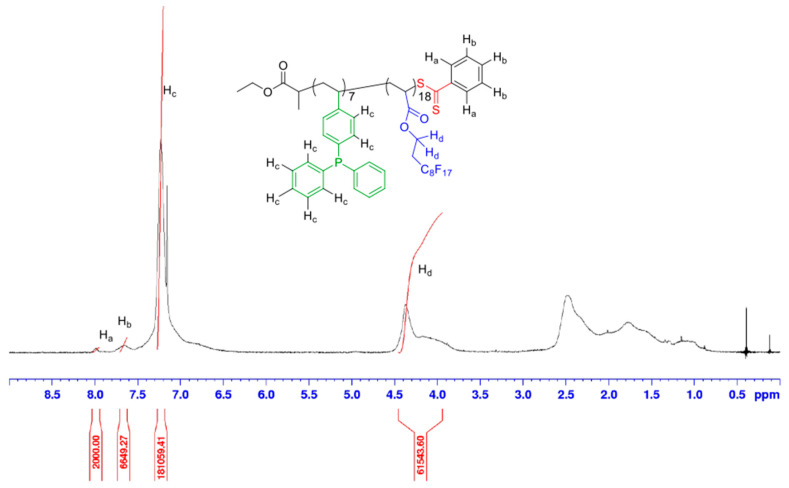
^1^H-NMR spectrum (400 MHz, CFC-113 + C_6_D_6_ capillaries) of P(DPPS-*co*-FDA) after precipitation.

P(DPPS-*co*-FDA) copolymer is only soluble in TFT and CFC-113 solvents, allowing exclusively to perform the ^1^H-NMR analysis using C_6_D_6_ capillaries for locking (Figure 5). A broad peak between δ = 6.5 and 8 ppm can be observed, related to the aromatic protons of the phosphonic monomer.

The degrees of polymerization and the molecular weight of the copolymer P(DPPS-*co*-FDA) were estimated using Equations (S19)–(S21). They were estimated to be equal to M_n,precipitated,P(DPPS-*co*-FDA)_ = 11,640 g/mol with DP_DPPS_ = 7.5 and DP_FDA_ = 17.8 (Figure 5).

**Figure 6:**
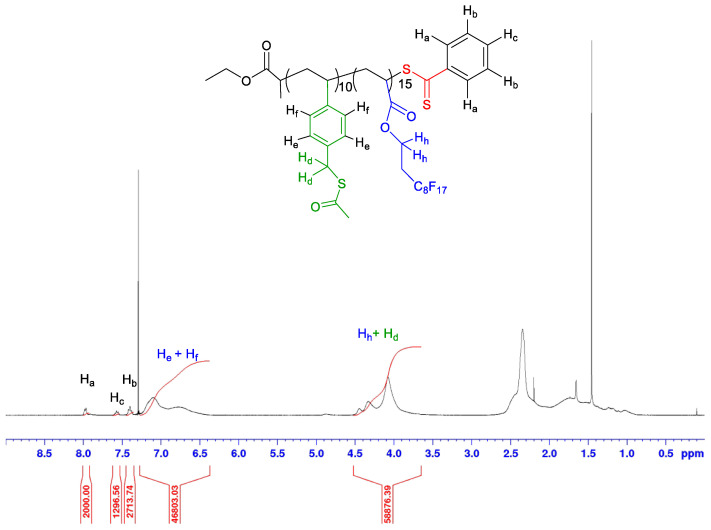
^1^H-NMR spectrum (400 MHz, CDCl_3_) of P(StySAc-*co*-FDA) after precipitation.

P(StySAc-*co*-FDA) copolymer is slightly soluble in acetone, and soluble in chloroform. Its ^1^H-NMR spectrum shows a broad peak between δ = 6.5 and 7 ppm relative to the aromatic protons of the StySAc monomer (Figure 6).

To calculate the degrees of polymerization and the molecular weight of the P(StySAc-*co*-FDA), it needs to take into account the overlapping at δ = 4 ppm of the typical methylene signal of the FDA units with the benzylic signal of StySAc units, using Equations (S26)–(S28). A copolymer with a molecular weight of 9790 g/mol and degrees of polymerization of 9.7 for the StySAc complexing monomer and 14.8 for FDA was obtained.

Through all these data, it was possible to estimate the weight fractions of complexing monomer units and FDA monomer units for all the copolymers according to general Equations (S2) and (S3), respectively, (see SI Section S2). Weight fractions of complexing monomer units ranging from 0wt% for P(FDA) homopolymer to 30.6wt% for P(AAEM-*co*-FDA) copolymer were thus estimated.

(Co)polymer structures were also confirmed by MALDI-TOF-MS analyses using reflectron positive ion mode (see SI Section S2.6).

**Figure 7:**
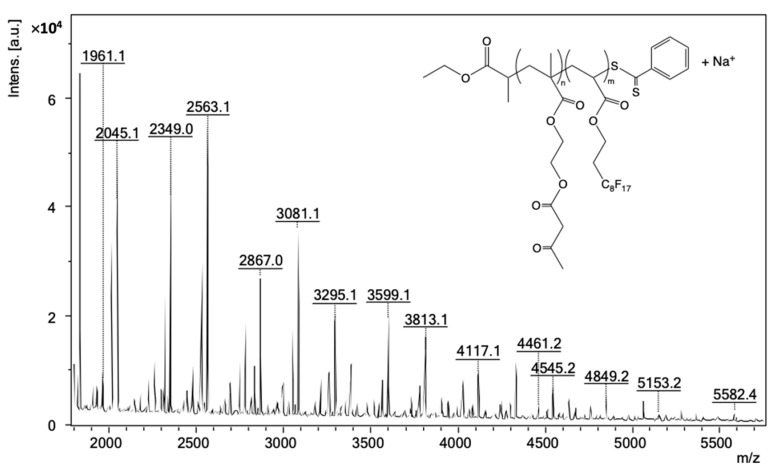
MALDI-TOF-MS mass spectrum in positive ion mode of P(AAEM-*co*-FDA) gradient copolymer with DCTB as matrix and sodium trifluoroacetate as cationizing agent.

The MALDI-TOF-MS mass spectrum of P(AAEM-*co*-FDA) was detected between 1800 and 5750 *m*/*z* (Figure 7) although M_n,NMR_ of the precipitated copolymer was estimated to be equal to 13,480 g/mol. Such discrepancy is not surprising as it is well known that high-accuracy mass measurements are possible for oligomer samples only with a mass range no greater than 3000 *m*/*z* [79].

The main population corresponds to the species ionized with sodium with the formula CH_3_CH_2_OC(O)CHCH_3_(AAEM_n_-*co*-FDA_m_)SC(S)C_6_H_5_ + Na^+^, i.e., one CTA end-group associated with some units of AAEM and some units of FDA monomer incremented with one sodium cation due to ionization.

**Figure 8:**
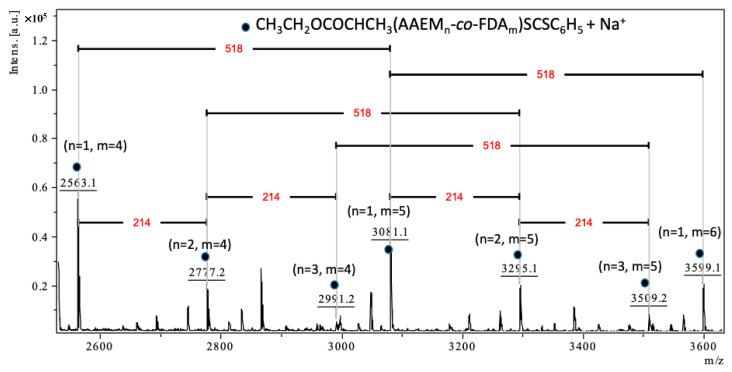
Enlarged MALDI-TOF-MS mass spectrum in positive ion mode of P(AAEM-*co*-FDA) (zoom between 2500 and 3610 *m*/*z*).

A more detailed analysis of the MALDI-TOF-MS data in the region corresponding to the strongest peaks (2500–3610 *m*/*z*; Figure 8) shows both the presence and the combination of a series of peaks with differences to be either 518 (M_FDA_ = 518.02 *m*/*z*), (e.g., 2563.1 and 3081.1, 2777.2 and 3295.1, 3081.1 and 3599.1, etc.), or 214 (M_AAEM_ = 214.08 *m*/*z*), (e.g., 2563.1 and 2777.2, 2777.2 and 2991.2, 3295.1 and 3509.2, etc.). The number of repeating units of AAEM (n) and FDA (m) confirms the presence of copolymer. The comparison of the experimental mass/charge (*m*/*z*)_expt_ values with the calculated monoisotopic mass/charge (*m*/*z*)_calc_ values are presented in Table 2. Each peak of the copolymer was calculated according to Equation (1):

(*m*/*z*)_calc_ = n_AAEM_ × M_AAEM_ + m_FDA_ × M_FDA_ + M_CTA_ + M_Na._ (1)

where M_AAEM_ = 214.08 *m*/*z*, M_FDA_ = 518.02 *m*/*z*, M_CTA_ = 254.04 *m*/*z* and M_Na_ = 22.99 *m*/*z*

For instance, the calculated mass 2563.19 *m*/*z* = 1 × 214.08 + 4 × 518.02 + 254.04 + 22.99. The experimental mass value 2563.12 *m*/*z* is 0.069 Da different from the calculated mass. All the (*m*/*z*)_expt_ values for these series of peaks were in good correlation with the calculated (*m*/*z*)_calc_ values (Table 2).

**Figure 9:**
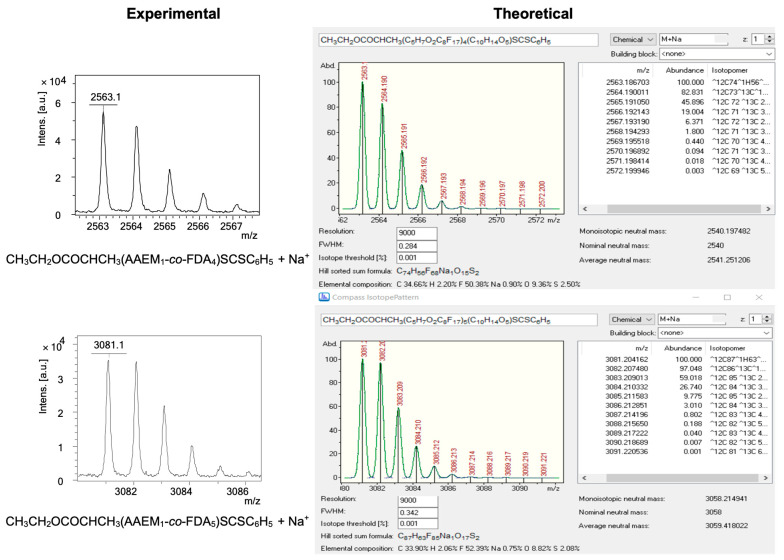
Experimental and theoretical isotope patterns of sodium adducts of CH_3_CH_2_OC(O)CHCH_3_(AAEM_n_-*co*-FDA_m_)SC(S)C_6_H_5_.

Lastly, note that the isotope patterns of theoretical and experimental sodium adducts of P(AAEM-*co*-FDA) copolymer exhibit a similar shape and are therefore in good agreement (Figure 9). Hence, the MALDI-TOF spectrum confirms the formation of the P(AAEM-*co*-FDA) copolymer.

P(FDA) homopolymer and four other P(FDA)-based copolymers constituted of complexing monomer units were therefore successfully synthesized by RAFT and well characterized.

### 3.2. Aminolysis of the (Co)Polymers

We took advantage of the RAFT technique to introduce a thiol group in our (co)polymers by aminolysis of the (co)polymer chain end. Indeed, thiols are known to complex some metals, (e.g., gold) and can therefore bring additional properties to our CO_2_-soluble metal-complexing (co)polymers. The aminolysis of the different fluorinated (co)polymers has been performed using three equivalents of piperidine. In order to avoid disulfide formation, triphenylphosphine was added as a reductant in a previously degassed solution of the polymer in TFT (SI Section S3).

**Figure 10:**
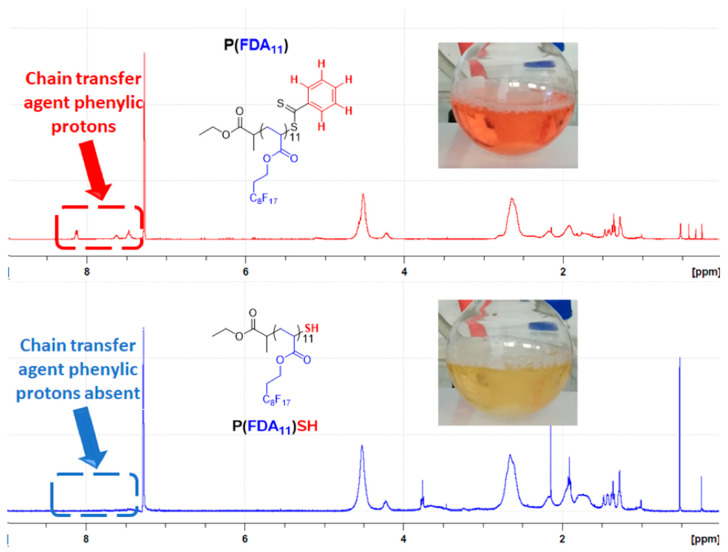
Comparison of the ^1^H-NMR spectra (400 MHz, CFC-113 + C_6_D_6_ capillaries) and of the color of the reaction mixture in the case of P(FDA) before (top) and after (bottom) aminolysis with piperidine.

Usually, end-group removal of RAFT polymers is performed by primary amines, such as hexylamine, butylamine, or cyclohexylamine [80,81]. The reaction can be easily followed through the color change of the polymer solution upon aminolysis. Indeed, a few seconds after the addition of piperidine, the solution switches from red color (typical of the phenylcarbonothioyl terminal group derived by the CTA) to a bright yellow color (typical of the phenylthioamide, which is the by-product of the aminolysis reaction) as shown in Figure 10.

**Figure 11:**
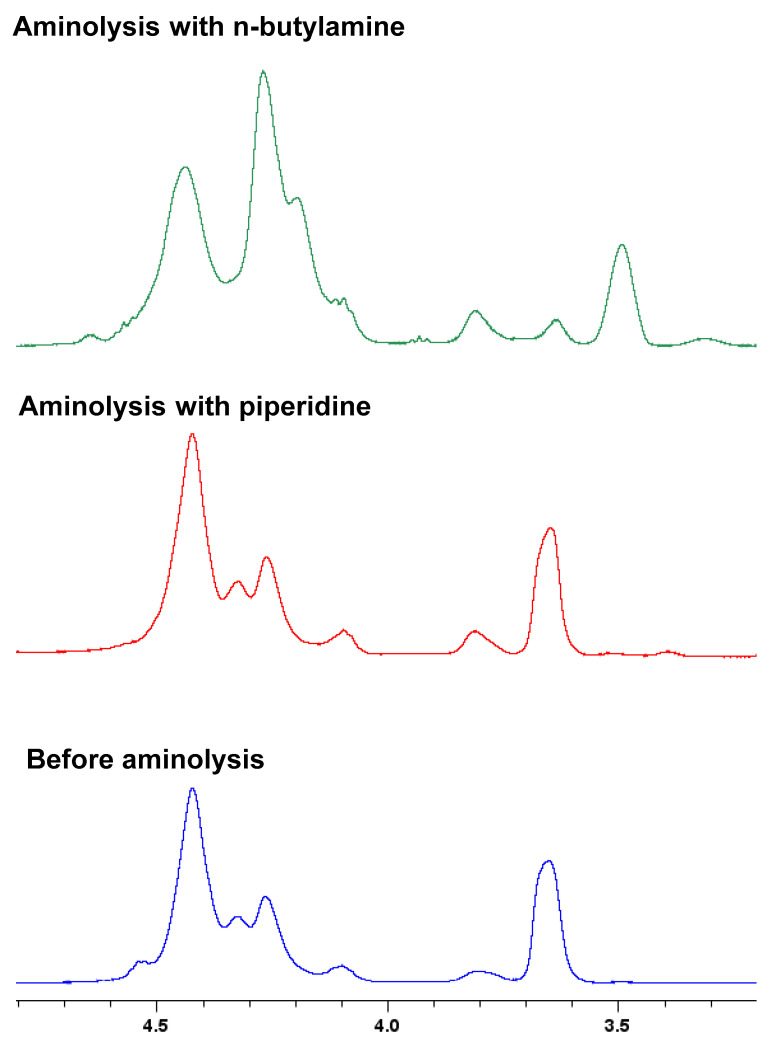
Comparison of ^1^H-NMR spectra (400 MHz, acetone-d_6_) of P(AAEM-*co*-FDA) before and after aminolysis using n-butylamine and piperidine.

Even if this end-group removal method can be applied to all our synthesized polymers, the use of n-butylamine (primary amine) led to a secondary reaction for the fluorinated copolymer containing AAEM units. Indeed, comparing the ^1^H-NMR spectra of the polymer before and after aminolysis by *n*-butylamine, it is possible to see some differences in the signal related to the acetoacetoxy protons (Figure 11).

In Figure 11, it is possible to identify two main peaks. The first peak (δ = 4–4.5 ppm) is related to the ethylenic protons of each monomer unit; meanwhile, the second (δ = 3.7 ppm) is attributed to the acetoacetate protons. The ratio between them changes depending on the different amines used to perform the aminolysis. This change is most probably due to the formation of enamine between butylamine and the acetoacetate group of AAEM [82,83]. Thus, for the sake of comparison, all the aminolyzed polymers reported in this work were obtained by using piperidine (secondary amine) as nucleophile. The ^1^H-NMR spectra of the aminolyzed copolymers are given in the SI (Figures S19–S24).

### 3.3. Thermal Polymers Characterization Pre- and Post-Aminolysis

The polymers before and after aminolysis were thermally characterized in order to investigate their thermal stability (TGA analyses under nitrogen) as well as the presence of thermal transitions (DSC analysis) (SI, Figures S25–S44) (Table 3). These analyses have been performed to study the influence of the different metal-complexing units of the copolymers and chain ends (pre- and post-aminolysis) on such gradient copolymers.

TGA analyses show T_2wt%loss_ (temperature where 2% of the weight is lost) higher than 200 °C for all the polymers, except the aminolyzed P(FDA)SH homopolymer (197 °C) and P(StySAc-*co*-FDA) copolymer (160 °C). In addition, higher T_2wt%loss_ are obtained for the copolymers after aminolysis (except for P(FDA) homopolymer): this behavior can be explained by the thermal elimination of the terminal phenyldithio group. Indeed, the cleavage of the dithio group in the case of unreacted CTA (R-S-C(S)Z, in this work, R = EtOC(O)CH(CH_3_)- and Z = Ph) occurs close to 200 °C, depending on the R group of the CTA. The higher residual mass by TGA after removing the end-group coming from the RAFT agent is also consistent with some works showing the lower thermal stability of RAFT polymers, even sometimes opening the route to a free depolymerization approach [84,85]. On the other hand, it is important to mention that the stability of the R group is increased by the integration of the CTA inside the polymer chain [81,86,87]. Thus, the following order of thermal stability was determined according to TGA results: P(FDA) > P(4VP-*co*-FDA) > P(AAEM-*co*-FDA) > P(DPPS-*co*-FDA) > P(StySAc-*co*-FDA).

DSC analyses show that P(FDA) and P(FDA)SH homopolymers present a melting temperature (T_m_), respectively, at 63 and 56 °C, before and after aminolysis, while no glass transition (T_g_) was detected for both homopolymers. On the other hand, all the gradient copolymers exhibit a glass transition temperature and a melting temperature before and after aminolysis, except P(DPPS-*co*-FDA) and P(DPPS-*co*-FDA)SH copolymers that are 100% amorphous with only a T_g_ visible at 67 and 68 °C, respectively. Note that T_g_-values are higher after aminolysis of the gradient copolymers. A decrease in the T_g_ suggests lower intermolecular interactions between the polymer chains, which can be caused by the lower Lewis base character of AAEM and StySAc moieties compared to 4VP and DPPS.

### 3.4. Polymers Phase Behavior in Dense CO_2_

**Figure 12:**
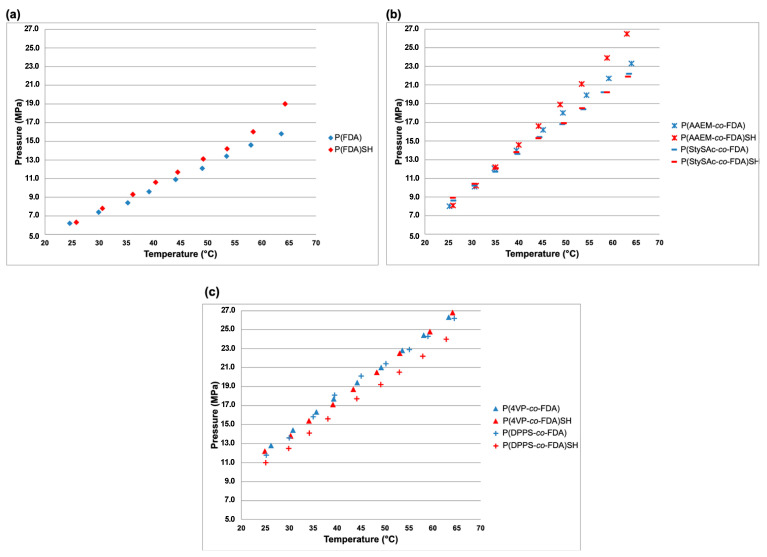
Cloud point curves in dense CO_2_ for the synthesized polymers: (**a**) P(FDA) and P(FDA)SH; (**b**) P(AAEM-*co*-FDA), P(AAEM-*co*-FDA)SH, P(StySAc-*co*-FDA) and P(StySAc-*co*-FDA)SH; (**c**) P(4VP-*co*-FDA), P(4VP-*co*-FDA)SH, P(DPPS-*co*-FDA) and P(DPPS-*co*-FDA)SH (at a polymer concentration of ca. 1 wt% of polymer relative to CO_2_).

The phase behavior in dense CO_2_ was studied for all the synthesized polymers, in both their pre- and post-aminolysis forms (SI Section S5, Figures S45–S55). To this aim, the cloud point measurements were performed in liquid and supercritical CO_2_. This analysis allows determining the transition from a cloudy CO_2_-polymer suspension to a transparent solution. This transition indicates the cloud point from the solubilized polymer (transparent solution) to the cloudy suspension (polymer insoluble) and vice versa. The obtained data, recorded in a cooling process (meaning screening the temperature from 65 down to 25 °C), are shown in Figure 12 and Figure S55.

All the synthesized polymers are soluble at pressures lower than 27 MPa up to 65 °C, which can be considered mild conditions. For the sake of comparison, decaffeination by scCO_2_, which is a major industrial application of supercritical fluids, is routinely operated at a pressure of 22 MPa and a temperature of 90 °C [88]. Mild operating conditions represent an important parameter for the intended application of the synthesized polymers in metal recovery.

Notably, three different trends can be identified in Figure 12 from the non-aminolyzed polymers. The first one corresponds to the P(FDA) homopolymer which shows the lowest cloud point pressures at a given temperature (Figure 12a). A second trend corresponds to the fluorinated copolymers incorporating AAEM and StySAc units, showing intermediate cloud point pressures (Figure 12b). Finally, a third trend corresponds to the copolymer containing DPPS and 4VP units, showing the highest cloud point pressures (Figure 12c). Regarding these last two different trends, it is interesting to notice that, going back to the purification step of the copolymers, the ones with lower cloud point pressures formed a gum during the precipitation. Furthermore, AAEM and StySAc copolymers showed a rather low T_g_ (8 and 24 °C, respectively). On the contrary, the copolymer containing DPPS and 4VP units have shown an easier precipitation behavior during the purification step, forming a powder and showing a higher glass transition temperature (67 and 61 °C, respectively). These facts allow us to draw a correlation between the T_g_ of the copolymers and their solubility in dense CO_2_: the higher T_g_ indicates an increased interaction between the polymer chains, decreasing the solubility of the polymers in dense CO_2_ [89]. These behaviors can be additionally explained by the nature of the comonomers. Indeed, it has been proved that monomers such as pyridine have a favorable nitrogen–nitrogen interaction instead of nitrogen-CO_2_ once incorporated in a polymeric chain, bringing a reduced solubility in dense CO_2_ [90]. Instead, the addition of acetate groups in the polymer chain increases the number of electron-rich groups in the system, improving its CO_2_-philicity [91]. In summary, the following decreasing order of solubility of the polymers has been determined from the cloud point curves: P(FDA) > P(StySAc-*co*-FDA) ≅ P(AAEM-*co*-FDA) > P(DPPS-*co*-FDA) ≅ P(4VP-*co*-FDA).

Furthermore, the solubility of the aminolyzed polymers has also been investigated in dense CO_2_. Interestingly, the aminolysis reaction, with the introduction of a polar thiol group as polymer chain end instead of dithiobenzoate moiety, did not influence significantly the solubility of these polymers: the cloud point pressure only increased by a few MPa (less than 3.2 MPa, in the worst case of P(AAEM-*co*-FDA) at 65 °C) at a given temperature. Thus, this limited difference in cloud point pressure does not preclude the good solubility in dense CO_2_ of such thiol-terminated polymers. The mild conditions necessary to solubilize this wide range of polymers in a green solvent such as CO_2_ demonstrate the remarkable relevance of this library of metal-complexing CO_2_-soluble polymers for their application as extractants for the removal of metals from industrial or urban waste.

### 3.5. Ability of Polymers to Complex Metals

Preliminary experiments have already been performed by our team to verify the ability of such polymers to complex metals. Thus, P(AAEM*-co-*FDA) copolymers showed their ability to be used as palladium ligands [92]. Complexation of palladium acetate was performed by the complexing AAEM units of the copolymer leading to a P(AAEM*-co-*FDA)-Pd^II^ supramolecular complex. The resulting polymer-supported Pd catalyst was successfully used for various applications such as a catalyst for Heck reaction, generation of Pd^0^ nanoparticles in the polymer matrix, or Pd^II^ impregnation of mesoporous silica in scCO_2_ followed by reduction to Pd^0^ (preparation of silica-supported Pd catalyst). In addition to Pd, P(AAEM*-co-*FDA) copolymer has also shown its ability to complex cobalt and more precisely cobalt acetate hydrate. Complexes Co-P(AAEM-*co*-FDA) exhibit cloud point pressure approximately 30 to 40 bar higher than the copolymer alone [93]. Thiol-terminated homopolymer P(FDA)SH, P(DPPS*-co-*FDA) and aminolized P(DPPS*-co-*FDA)SH gradient copolymers were used to extract palladium from commercial Pd/Al_2_O_3_ supported catalysts [53]. Extractions were performed in scCO_2_ under mild conditions (40 °C and 25 MPa). Polymers were found to successfully extract up to ca. 40% of Pd from the Pd/Al_2_O_3_ catalysts. P(DPPS*-co-*FDA)SH was the most efficient system, requiring a much lower quantity than the two other polymers to achieve the same level of extraction in one hour. Lastly, P(4VP*-co-*FDA) and aminolyzed P(4VP*-co-*FDA)SH gradient copolymers showed their ability to extract Pd from the aluminosilica-supported catalyst in scCO_2_ under mild conditions (40 °C and 25 MPa). More precisely, ca. 70% of the Pd was removed from an aluminosilica support in the presence of P(4VP*-co-*FDA) copolymer [54].

These different metal-complexing CO_2_-philic polymers demonstrated their ability to be used for various applications. These promising results urged us to enlarge the panel of such polymers and to detail their synthesis method.

## 4. Conclusions

In this work, we have presented the synthesis of ten (co)polymers which are constituted by a CO_2_-philic fraction, represented by FDA monomer units, and by CO_2_-phobic complexing monomer units (4VP, AAEM, DPPS, and StySAc). These (co)polymers were successfully synthesized by the reversible-deactivation radical polymerization technique (RAFT). ^1^H-NMR characterization allowed us to determine the composition as well as the molecular weight of the synthesized (co)polymers. In addition, the thermal properties of the (co)polymers have been studied by TGA and DSC, which confirms the good thermal stability of the (co)polymers (decreasing order of thermal stability: T_2wt%loss,P(FDA)_ = 263 °C > T_2wt%loss,P(4VP-*co*-FDA)_ = 249 °C > T_2wt%loss,P(AAEM-*co*-FDA)_ = 239 °C > T_2wt%loss,P(DPPS-*co*-FDA)_ = 215 °C > T_2wt%loss,P(StySAc-*co*-FDA)_ = 160 °C) and their difference in T_m_ (T_m,P(AAEM-*co*-FDA)_ = 41 °C < T_m,P(StySAc-*co*-FDA)_ = 51 °C < T_m,P(FDA)_ = 63 °C < T_m,P(4VP-*co*-FDA)_ = 85 °C) and T_g_ transition temperatures (T_g,P(AAEM-*co*-FDA)_ = 8 °C < T_g,P(StySAc-*co*-FDA)_ = 24 °C < T_g,P(4VP-*co*-FDA)_ = 61 °C < T_g,P(DPPS-*co*-FDA)_ = 67 °C). Finally, their phase behavior in dense CO_2_ has been studied by cloud point measurements, showing that these metal-complexing (co)polymers are soluble at mild pressure conditions (lower than 27 MPa at 65 °C), which is a key factor for their eventual application as metal extraction agents. Through the synthesis of well-defined copolymers (gradient architecture, monomer composition, and polymer molecular weight) and their promising CO_2_ solubility, this library of metal-complexing CO_2_-soluble polymers show good candidates for the recovery or the decontamination of end-of-life metals-containing liquid or solid wastes. The investigation of their performances in metal extractions from supported catalysts is in progress and will be reported in forthcoming articles.

## Figures and Tables

**Table 1:** Synthesis by RAFT polymerization of P(FDA) and gradient complexing copolymers.

(Co)polymer ^a^	M_n,targeted_ ^c^ (g/mol)	DP_complexing mono_^d^	DP_FDA_ ^d^	M_n,NMR_ ^d^ (g/mol)	f_complexing mono_ ^e^ (wt%)	f_FDA_ ^f^ (wt%)	Yield ^g^ (%)
**P(FDA)**	5020	0	10.8	5850	0	100	78
**P(4VP-*co*-FDA) ^b^**	10,250	19.8	18.3	11,820	18.0	82.0	59
**P(AAEM-*co*-FDA)**	10,090	18.9	17.7	13,480	30.6	69.4	43
**P(DPPS-*co*-FDA)**	10,060	7.5	17.8	11,640	19.0	81.0	61
**P(StySAc-*co*-FDA)**	10,060	9.7	14.8	9790	19.6	80.4	55

^a^ (Co)polymerization of FDA and complexing monomers by RAFT in TFT at 65 °C with molar ratio AIBN/CTA = 0.3. ^b^ Use of toluene instead of TFT as reaction solvent. ^c^ M_n,targeted_ = ((m_FDA_ + m_complexing monomer_)/n_CTA_) + M_CTA_, where m_FDA_ and m_complexing monomer_ are the mass of FDA and complexing monomers, n_CTA_ is the moles of CTA necessary for the polymerization and M_CTA_ is the molecular weight of the chain end groups (254.36 g/mol). ^d^ Determined by ^1^H-NMR peak intensity ratio. ^e^ f_complexing mono_ = fraction of complexing monomer unit = ratio of the weight of complexing monomer units with respect to the total weight of complexing monomer and FDA, determined by ^1^H-NMR peak intensity ratio. ^f^ f_FDA_ = fraction of FDA monomer unit = ratio of the weight of FDA units with respect to the total weight of complexing monomer and FDA, determined by ^1^H-NMR peak intensity ratio. ^g^ Yield = (m_(co)polymer_/(m_complexing monomer_ + m_FDA_ + m_CTA_)) × 100.

**Table 2:** Assignments of peaks in P(AAEM-*co*-FDA) with n AAEM units and m FDA units.

(*m*/*z*)_expt_	n	m	(*m*/*z*)_calc_^a^	∆ = |(*m*/*z*)_expt_ − (*m*/*z*)_calc_|
2563.12	1	4	2563.19	0.069
2777.20	2	4	2777.27	0.070
2991.25	3	4	2991.35	0.100
3081.10	1	5	3081.20	0.100
3295.15	2	5	3295.29	0.140
3509.25	3	5	3509.37	0.120
3599.10	1	6	3599.22	0.120

^a^ Calculated from the monoisotopic mass.

**Table 3:** Thermal properties of the synthesized polymers.

(Co)Polymer	TGA		DSC	
	T_2wt%loss_ (°C)	Residual mass at 570 °C(%)	T_g_^a^ (°C)	T_m_^b^ (°C)
**P(FDA)**	263	0.3	-	63
**P(FDA)SH**	197	1.3	-	56
**P(4VP-*co*-FDA)**	249	9.3	61	85
**P(4VP-*co*-FDA)SH**	274	31.9	67	89
**P(AAEM-*co*-FDA)**	239	10.0	8	41
**P(AAEM-*co*-FDA)SH**	280	31.4	15	42
**P(DPPS-*co*-FDA)**	215	13.7	67	-
**P(DPPS-*co*-FDA)SH**	262	16.7	68	-
**P(StySAc-*co*-FDA)**	160	1.7	24	51
**P(StySH-*co*-FDA)SH**	298	6.3	44	65

^a^ glass transition temperature T_g_, ^b^ melting temperature T_m_.

## Data Availability

The data presented in this study are available in the Supporting Information.

## Supplementary Materials

The following supporting information can be downloaded at: https://www.mdpi.com/article/10.3390/polym14132698/s1, Figure S1: Reaction scheme for the synthesis of CTA; Figure S2: ^1^H-NMR spectrum (400 MHz, CDCl_3_) of CTA; Figure S3: Reaction scheme for the synthesis of StySAc monomer; Figure S4: ^1^H-NMR spectrum (400 MHz, CDCl_3_) of StySAc monomer; Figure S5: ^1^H-NMR spectrum (400 MHz, toluene + C_6_D_6_ capillaries) of P(4VP-*co*-FDA) at t = 72 h; Figure S6: ^1^H-NMR spectrum (400 MHz, TFT + C_6_D_6_ capillaries) of P(DPPS-*co*-FDA) at t = 48 h; Figure S7: ^1^H-NMR spectrum (400 MHz, TFT + C_6_D_6_ capillaries) of P(StySAc-*co*-FDA) at t = 48 h; Figure S8: MALDI-TOF-MS mass spectrum in positive ion mode of P(FDA) homopolymer with DCTB as matrix and sodium trifluoroacetate as cationizing agent; Figure S9: Enlarged MALDI-TOF-MS mass spectrum in positive ion mode of P(FDA) homopolymer (zoom between 4350 and 5500 *m*/*z*); Figure S10: MALDI-TOF-MS mass spectrum in positive ion mode of P(4VP-*co*-FDA) with DCTB as matrix and sodium trifluoroacetate as cationizing agent; Figure S11: Enlarged MALDI-TOF-MS mass spectrum in positive ion mode of P(4VP-*co*-FDA) (zoom between 3460 to 4040 *m*/*z*); Figure S12: Experimental and theoretical isotope patterns of proton adducts of CH_3_CH_2_OCOCHCH_3_(4VP_n_-*co*-FDA_m_)SCSC_6_H_5_; Figure S13: MALDI-TOF-MS mass spectrum in positive ion mode of P(DPPS-*co*-FDA) with dithranol as matrix and NaI as cationizing agent; Figure S14: Enlarged MALDI-TOF-MS mass spectrum in positive ion mode of P(DPPS-*co*-FDA) (zoom between 2960 to 3780 *m*/*z*); Figure S15: Zoom between 3388 and 3455 *m*/*z* of MALDI-TOF-MS mass spectrum in positive ion mode of P(DPPS-*co*-oxidized DPPS-*co*-FDA) copolymer to evidence the mass difference of +16 *m*/*z*; Figure S16: MALDI-TOF-MS mass spectrum in positive ion mode of P(StySAc-*co*-FDA) with DCTB as matrix and sodium trifluoroacetate as cationizing agent; Figure S17: Enlarged MALDI-TOF-MS mass spectrum in positive ion mode of P(StySAc-*co*-FDA) (zoom between 5560 to 6180 *m*/*z*); Figure S18: Experimental and theoretical isotope patterns of sodium adducts of CH_3_CH_2_OCOCHCH_3_(StySAc_n_-*co*-FDA_m_)SCSC_6_H_5_; Figure S19: ^1^H-NMR spectrum (400 MHz, CFC-113 + C_6_D_6_ capillaries) of P(FDA)SH after precipitation; Figure S20: ^1^H-NMR spectrum (400 MHz, CDCl_3_) of P(4VP-*co*-FDA)SH after precipitation; Figure S21: ^1^H-NMR spectrum (400 MHz, acetone-d_6_) of P(AAEM-*co*-FDA)SH after precipitation, obtained by aminolysis with piperidine; Figure S22: ^1^H-NMR spectrum (400 MHz, acetone-d_6_) of P(AAEM-*co*-FDA)SH after precipitation, obtained by aminolysis reaction with n-butylamine; Figure S23: ^1^H-NMR spectrum (400 MHz, CFC-113 + C_6_D_6_ capillaries) of P(DPPS-*co*-FDA)SH after precipitation; Figure S24: ^1^H-NMR spectrum (400 MHz, CDCl_3_) of P(StySH-*co*-FDA)SH after precipitation; Figure S25: TGA measurement of P(FDA); Figure S26: DSC measurement of P(FDA); Figure S27: TGA measurement of P(FDA)SH; Figure S28: DSC measurement of P(FDA)SH; Figure S29: TGA measurement of P(4VP-*co*-FDA), Figure S30: DSC measurement of P(4VP-*co*-FDA); Figure S31: TGA measurement of P(4VP-*co*-FDA)SH; Figure S32: DSC measurement of P(4VP-*co*-FDA)SH; Figure S33: TGA measurement of P(AAEM-*co*-FDA); Figure S34: DSC measurement of P(AAEM-*co*-FDA); Figure S35: TGA measurement of P(AAEM-*co*-FDA)SH; Figure S36: DSC measurement of P(AAEM-*co*-FDA)SH; Figure S37: TGA measurement of P(DPPS-*co*-FDA); Figure S38: DSC measurement of P(DPPS-*co*-FDA); Figure S39: TGA measurement of P(DPPS-*co*-FDA)SH; Figure S40: DSC measurement of P(DPPS-*co*-FDA)SH; Figure S41: TGA measurement of P(StySAc-*co*-FDA); Figure S42: DSC measurement of P(StySAc-*co*-FDA); Figure S43: TGA measurement of P(StySH-*co*-FDA)SH; Figure S44: DSC measurement of P(StySH-*co*-FDA)SH; Figure S45: Cloud Point measurement for P(FDA); Figure S46: Cloud Point measurement for P(FDA)SH; Figure S47: Cloud Point measurement for P(4VP-*co*-FDA); Figure S48: Cloud Point measurement for P(4VP-*co*-FDA)SH; Figure S49: Cloud Point measurement for P(AAEM-*co*-FDA); Figure S50: Cloud Point measurement for P(AAEM-*co*-FDA)SH; Figure S51: Cloud Point measurement for P(DPPS-*co*-FDA); Figure S52: Cloud Point measurement for P(DPPS-*co*-FDA)SH; Figure S53: Cloud Point measurement for P(StySAc-*co*-FDA); Figure S54: Cloud Point measurement for P(StySH-*co*-FDA)SH; Figure S55: Cloud point curves in dense CO_2_ for the synthesized polymers; Table S1: Assignments of peaks in P(4VP-*co*-FDA) copolymer, with n 4VP units and m FDA units; Table S2: Assignments of peaks in P(DPPS-*co*-FDA), with n DPPS units and m FDA units; Table S3: Assignments of peaks in P(DPPS-*co*-oxidized DPPS-*co*-FDA), with n DPPS units, x oxidized DPPS units and m FDA units; Table S4: Assignments of peaks in P(StySAc-*co*-FDA), with n StySAc units and m FDA units.
