# Supplementary material for: Synthesis and Phase Behavior of a Platform of CO2-Soluble Functional Gradient Copolymers Bearing Metal-Complexing Units
Source: Polymers (Basel). 2022 Jun 30;14(13):2698. doi: 10.3390/polym14132698 (PMC9269141; doi:10.3390/polym14132698)
Supplement: Supplementary file 1 [file polymers-14-02698-s001.zip › polymers-1758213-SM.pdf]

Supporting information

Synthesis and phase behavior of a platform of CO_2-soluble functional gradient copolymers bearing metal-complexing units

Andrea Ruiu [1], Cécile Bouilhac [1,*], Olinda Gimello [1], Karine Seaudeau-Pirouley [2], Marin Senila [3], Thorsten Jänisch [4] and Patrick Lacroix-Desmazes [1,*]

[1] ICGM, Univ. Montpellier, CNRS, ENSCM, 34293 Montpellier, France; andrea1.ruiu@gmail.com (A.R.); olinda.gimello@enscm.fr (O.G.)
[2] Innovation Fluides Supercritiques, Batiment INEED, 26300 Alixan, France; k.seaudeau@supercriticalfluid.org (K.S.-P.)
[3] INCDO INOE 2000, Research Institute for Analytical Instrumentation, 400293 ICIA,Cluj-Napoca, Romania; marin.senila@icia.ro (M.S.)
[4] Fraunhofer Institute for Chemical Technology, 76327 Pfinztal, Germany; tjaenisch82@gmail.com (T.J.)
* Correspondence: patrick.lacroix-desmazes@enscm.fr (P.L.-D.); cecile.bouilhac@umontpellier.fr (C.B.)

Table of content

S1. Reactant synthesis ... 3
S1.1 CTA Synthesis ... 3
S1.2 StySAc Synthesis ... 3
S2. Polymer syntheses ... 5
S2.1 FDA homopolymerization ... 5
S2.2 FDA-4VP copolymerization ... 5
S2.3 FDA-AAEM copolymerization ... 7
S2.4 FDA-DPPS copolymerization ... 8
S2.5 FDA-StySAc copolymerization ... 10
S2.6 MALDI-TOF analyses ... 12
S3. Polymer after aminolysis ... 21
S3.1 P(FDA)SH ... 22
S3.2 P(4VP-co-FDA)SH ... 23
S3.3 P(AAEM-co-FDA)SH (piperidine aminolysis) ... 24
S3.4 P(AAEM-co-FDA)SH (n-Butylamine aminolysis) ... 26
S3.5 P(DPPS-co-FDA)SH ... 27
S3.6 P(StySH-co-FDA)SH ... 28
S4. Thermal characterization of the polymers ... 29
S4.1 P(FDA) ... 29
S4.2 P(FDA)SH ... 30

S4.3 P(4VP-co-FDA) 31
S4.4 P(4VP-co-FDA)SH 32
S4.5 P(AAEM-co-FDA) 33
S4.6 P(AAEM-co-FDA)SH 34
S4.7 P(DPPS-co-FDA) 35
S4.8 P(DPPS-co-FDA)SH 36
S4.9 P(StySAc-co-FDA) 37
S4.10 P(StySH-co-FDA)SH 38
S5. Cloud-Point measurements 39
S5.1 P(FDA) 39
S5.2 P(FDA)SH 40
S5.3 P(4VP-co-FDA) 41
S5.4 P(4VP-co-FDA)SH 42
S5.5 P(AAEM-co-FDA) 43
S5.6 P(AAEM-co-FDA)SH 44
S5.7 P(DPPS-co-FDA) 45
S5.8 P(DPPS-co-FDA)SH 46
S5.9 P(StySAc-co-FDA) 47
S5.10 P(StySH-co-FDA)SH 48
S5.11 Overlay of the (co)polymer cloud points 49

S1. Reactant synthesis

S1.1 CTA Synthesis

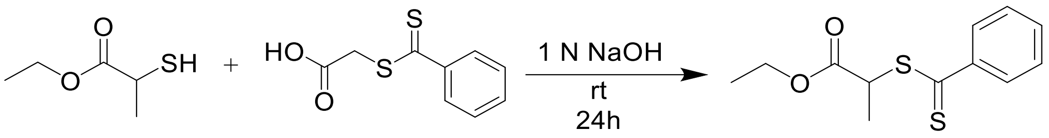

Figure S1. Reaction scheme for the synthesis of CTA.

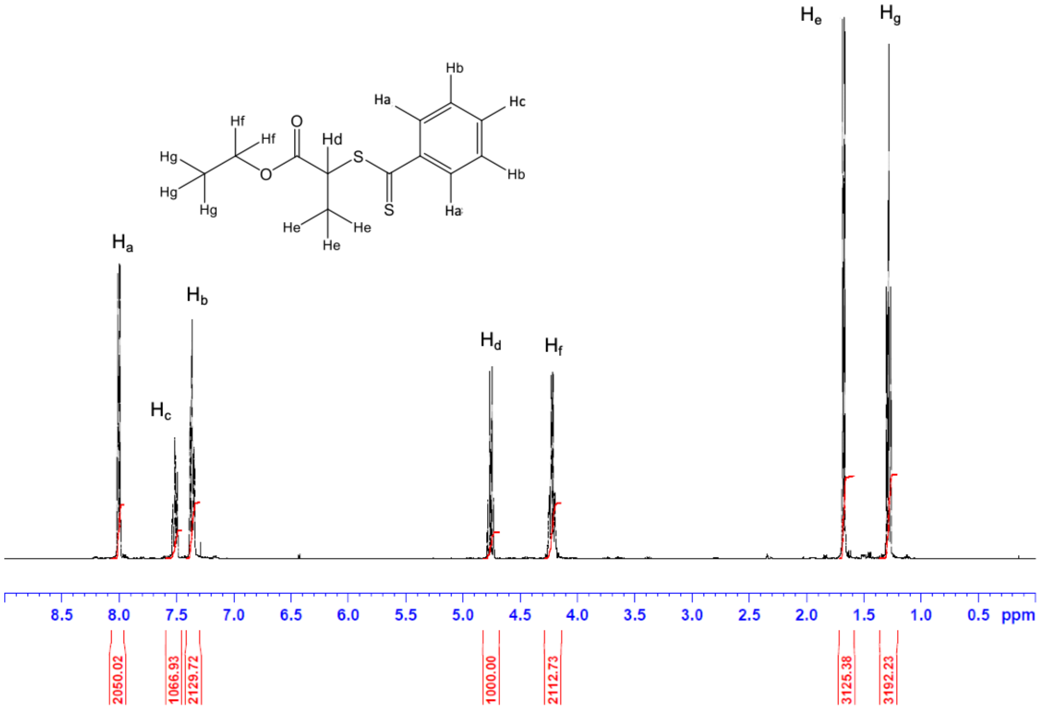

Figure S2. ^{1}H-NMR spectrum (400 MHz, $CDCl_3$) of CTA.

S1.2 StySAc Synthesis

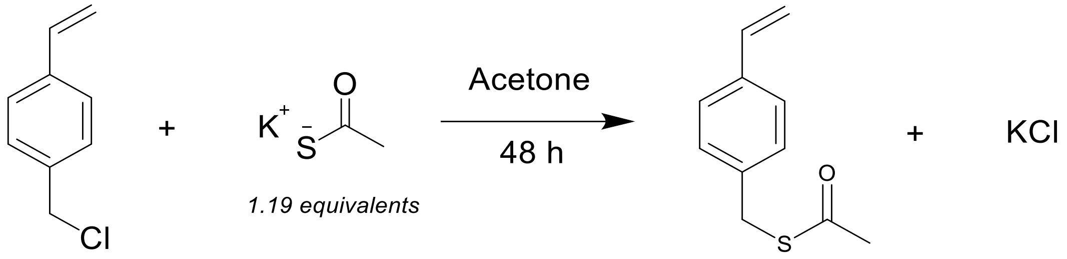

Figure S3. Reaction scheme for the synthesis of StySAc monomer.

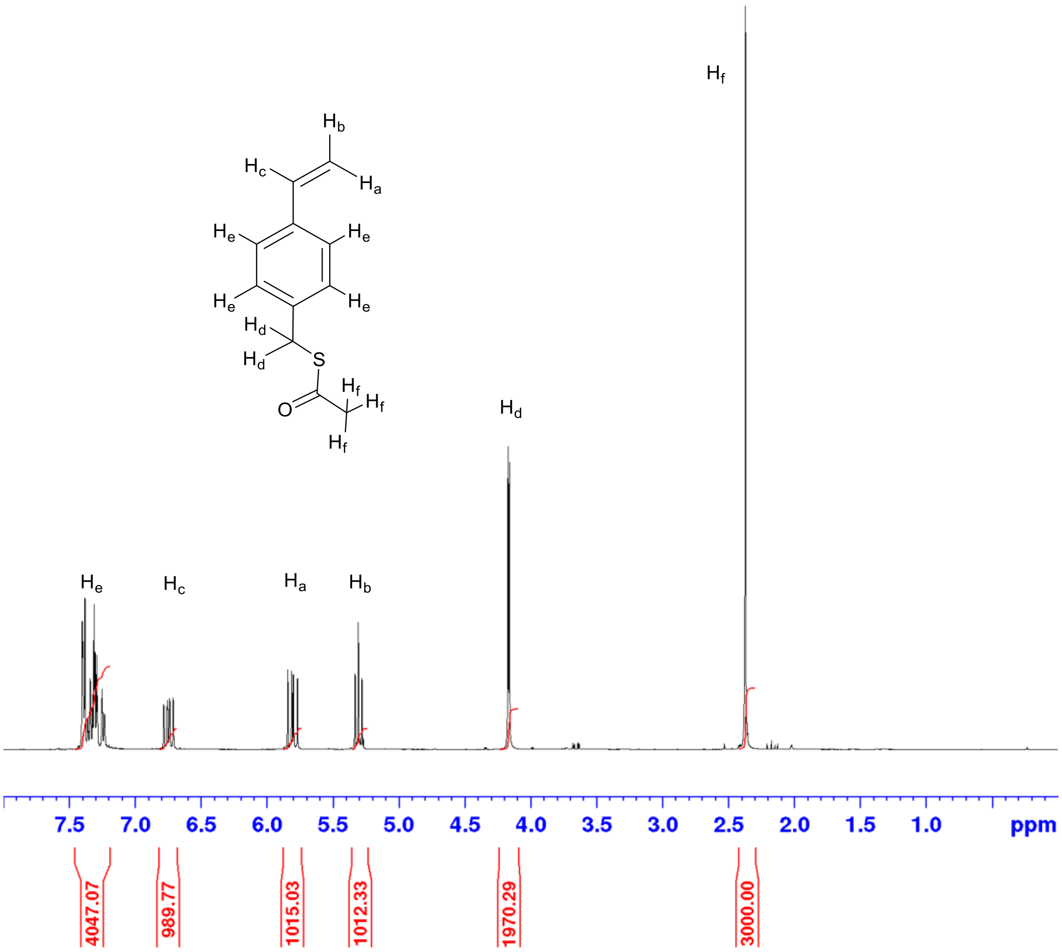

Figure S4.^{1}H-NMR spectrum (400 MHz, $CDCl_3$) of StySAc monomer

S2. Polymer syntheses

The amount of CTA (n_{CTA}) necessary to achieve the targeted molecular weight was calculated according to Equation S1:

$$n_{CTA} = \frac{\text{total mass of monomer}}{M_{n,targeted} - M_{CTA}} \quad \text{(S1)}$$

where n_{CTA}, $M_{n,targeted}$ and M_{CTA} represent the moles of CTA necessary for the polymerization, the targeted molecular weight (e.g. 10000 g/mol for the copolymers) and the CTA molecular weight (254.36 g/mol), respectively.

Furthermore, after estimation of the degrees of polymerization of the FDA and complexing monomers and knowing the molecular weight of the comonomers, it is possible to estimate the weight fractions of complexing monomer units and FDA monomer units for all the copolymers according to general Equation S2 and Equation S3:

$$f_{\text{complexing monomer}} = \frac{DP_{\text{complexing mono}} \times M_{\text{complexing mono}}}{(DP_{\text{complexing mono}} \times M_{\text{complexing mono}}) + (DP_{FDA} \times M_{FDA})} \times 100 \quad \text{(S2)}$$

$$f_{FDA} = 100 - f_{\text{complexing mono}} \quad \text{(S3)}$$

where complexing monomers are respectively 4VP, AAEM, DPPS and StySAc.

S2.1 FDA homopolymerization

$$DP_{FDA} = \frac{H_d/2}{(H_a + H_b + H_c)/5} = 10.8 \quad \text{(S4)}$$

where H_a, H_b, H_c and H_d correspond to the integrals associated to the protons a, b, c and d from CTA aromatic end-group and FDA.

$$M_{n,precipitated,P(FDA)}\ (g/mol) = DP_{FDA} \times M_{FDA} + M_{CTA} = 5850\ g/mol \quad \text{(S5)}$$

where M_{FDA} and M_{CTA} refer to the molecular weight of FDA (518.17 g/mol) and CTA (254.36 g/mol).

S2.2 FDA-4VP copolymerization

Note that in the case of copolymerization between FDA and 4VP, toluene was used instead of TFT as polymerization solvent. Indeed, the addition of 4VP in TFT caused the formation, in small proportion, of a white precipitate (not identified). A reaction with the fluorinated solvent was suspected [Salamone J.C.; Snider B.; Fitch W. L. Polymerization of 4-vinylpyridinium salts. I. The counterion initiation mechanism. *J. Polym. Sci. Part B Polym. Lett.* **1971**, *9*, 13–17]. However, a similar precipitate was also formed with toluene suggesting that both toluene or TFT could be used to perform the RAFT copolymerization.

However, after some time, the polymer started to precipitate (because toluene is not an enough good solvent for FDA-based copolymers), stacking the stirrer bar and interfering the proper agitation of the solution. In fact, it was not possible to properly sample the polymerization mixture at 120 hours. For this reason, the reaction was stopped.

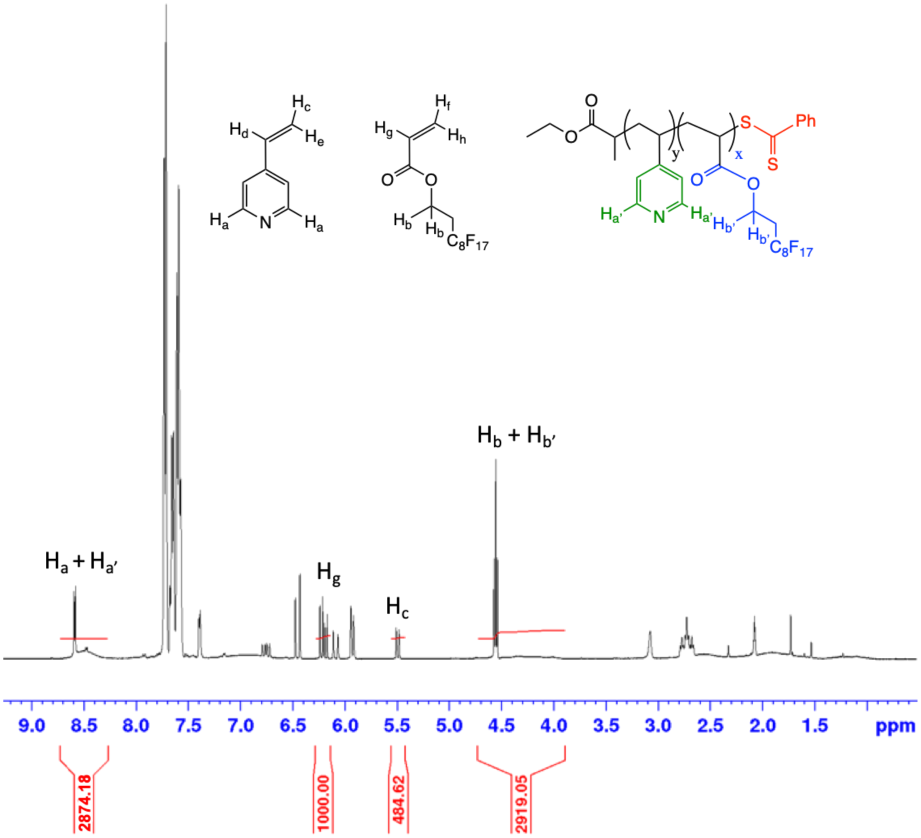

Figure S5. ^{1}H-NMR spectrum (400 MHz, toluene + C_6D_6 capillaries) of P(4VP-*co*-FDA) at t = 72 h

Monomer conversions at t = 72 h:

$$\text{4VP conversion (\%)} = \left(1 - \frac{\mathrm{H_c}}{(\mathrm{H_a} + \mathrm{H_{a'}})/_2}\right) \times 100 \tag{S6}$$

4VP conversion (%) = (1-(485/(2874/2))) x 100

4VP conversion (%) = 66%

$$\text{FDA conversion (\%)} = \left(1 - \frac{\mathrm{H_g}}{(\mathrm{H_b} + \mathrm{H_{b'}})/_2)}\right) \times 100 \tag{S7}$$

FDA conversion (%) = ((1-(1000/(2919/2))) x 100

FDA conversion (%) = 31%

Degrees of polymerization and molecular weight of the final copolymer:

$$DP_{4VP} = \frac{\left((H_e + H_f)/_4\right)}{(H_a + H_b + H_c)/_5} = 19.8 \quad (S8)$$

$$\frac{H_d/_2}{(H_a + H_b + H_c)/_5} = 18.3 \quad (S9)$$

$$M_{n,\text{precipitated P(4VP}-co-\text{FDA)}}\ (g/mol) = DP_{4VP} \times M_{4VP} + DP_{FDA} \times M_{FDA} + M_{CTA} = 11820\ g/mol \quad (S10)$$

where M_{4VP}, M_{FDA} and M_{CTA} refer to the molecular weight of 4VP (105.14 g/mol), FDA (518.17 g/mol) and CTA (254.36 g/mol).

Weight fractions of 4VP complexing monomer units and FDA monomer units in final copolymer:

$$f_{4VP} = \frac{DP_{4VP} \times M_{4VP}}{(DP_{4VP} \times M_{4VP}) + (DP_{FDA} \times M_{FDA})} \times 100 \quad (S11)$$

$$f_{4VP} = \frac{19.8 \times 105.14}{(19.8 \times 105.14) + (18.3 \times 518.17)} \times 100 = 18.0\ wt\%$$

$$f_{FDA} = 100 - f_{4VP} = 100 - 18.0 \quad (S12)$$

$$f_{FDA} = 82.0\ wt\%$$

S2.3 FDA-AAEM copolymerization

Evolution of each monomer conversion in the copolymerization of AAEM with FDA has already been reported by us [Ribaut T.; Lacroix-Desmazes P.; Fournel, B.; Sarrade S.; Synthesis of gradient copolymers with complexing groups by RAFT polymerization and their solubility in supercritical CO_2. *J. Polym. Sci. Part A: Polym. Chem.* **2009**, *47*, 5448–5460].

Degrees of polymerization and molecular weight of the final copolymer:

$$DP_{AAEM} = \frac{\left((H_f + H_e/_2\right)}{(H_a + H_b + H_c)/_5} = 18.9 \quad (S13)$$

$$DP_{FDA} = \frac{\left((H_g + H_d) - 4\left(H_f + H_e/_2\right)\right)/_2}{(H_a + H_b + H_c)/_5} = 17.7 \quad (S14)$$

$$M_{n,\text{precipitated P(AAEM}-co-\text{FDA)}}\ (g/mol) = DP_{AAEM} \times M_{AAEM} + DP_{FDA} \times M_{FDA} + M_{CTA} = 13480\ g/mol \quad (S15)$$

where M_{AAEM}, M_{FDA} and M_{CTA} refer to the molecular weight of AAEM (214.22 g/mol), FDA (518.17 g/mol) and CTA (254.36 g/mol).

Weight fractions of AAEM complexing monomer units and FDA monomer units in final copolymer:

$$f_{AAEM} = \frac{DP_{AAEM} \times M_{AAEM}}{(DP_{AAEM} \times M_{AAEM}) + (DP_{FDA} \times M_{FDA})} \times 100 \quad \text{(S16)}$$

$$f_{AAEM} = \frac{18.9 \times 214.22}{(18.9 \times 214.22) + (17.7 \times 518.17)} \times 100 = 30.6 \text{ wt\%}$$

$$f_{FDA} = 100 - f_{AAEM} = 100 - 30.6 \quad \text{(S17)}$$

$$f_{FDA} = 69.4 \text{ wt\%}$$

S2.4 FDA-DPPS copolymerization

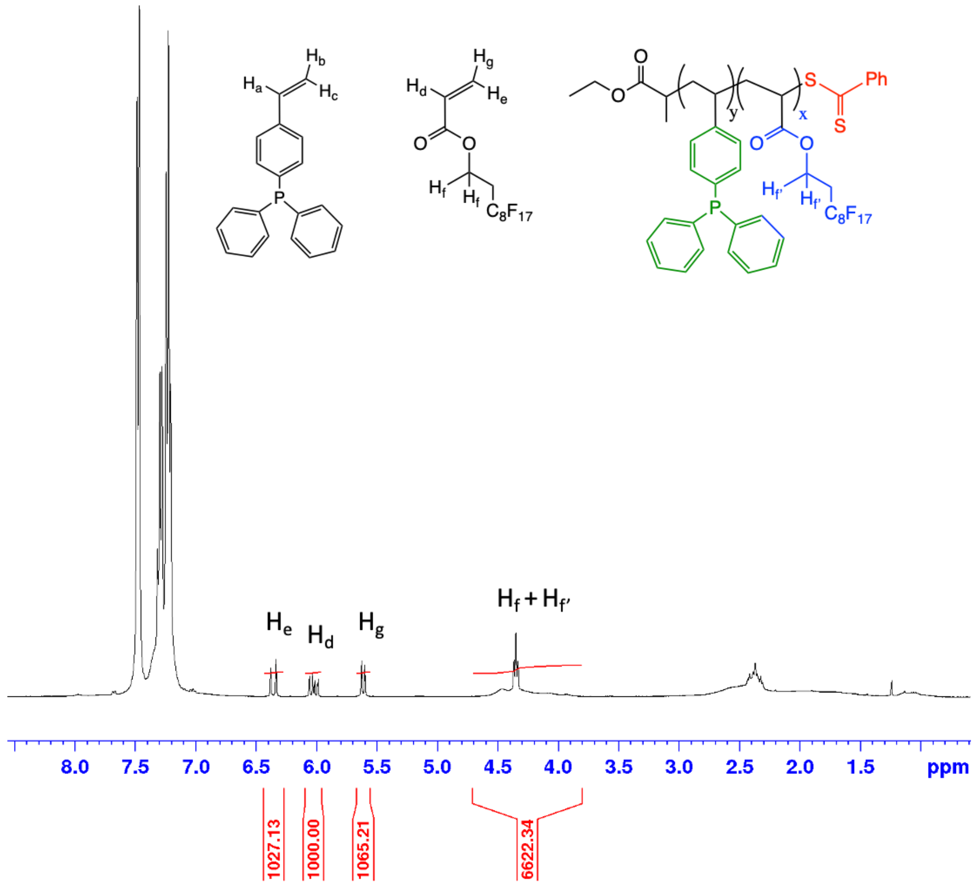

Figure S6. ^{1}H-NMR spectrum (400 MHz, TFT + C_6D_6 capillaries) of P(DPPS-*co*-FDA) at t = 48 h

Monomer conversions at t = 48 h:

DPPS Conversion (%) = 100%

$$\text{FDA Conversion (\%)} = \left(1 - \frac{(H_e + H_d + H_g)/3}{(H_f + H_{f'})/2}\right) \quad (S18)$$

FDA Conversion (%) = 1-((1027+1000+1065)/3)/(6622/2) = 69%

Degrees of polymerization and molecular weight of the final copolymer:

$$DP_{DPPS} = \frac{H_c/14}{(H_a + H_b)/5} = 7.5 \quad (S19)$$

$$DP_{FDA} = \frac{H_d/2}{(H_a + H_b)/5} = 17.8 \quad (S20)$$

$$M_{n,\text{precipitated }P(DPPS-co-FDA)}\ (g/mol) = DP_{DPPS} \times M_{DPPS} + DP_{FDA} \times M_{FDA} + M_{CTA} = 11640\ g/mol \quad (S21)$$

where M_{DPPS}, M_{FDA} and M_{CTA} refer to the molecular weight of DPPS (288.32 g/mol), FDA (518.17 g/mol) and CTA (254.36 g/mol).

Weight fractions of DPPS complexing monomer units and FDA monomer units in final copolymer:

$$f_{DPPS} = \frac{DP_{DPPS} \times M_{DPPS}}{(DP_{DPPS} \times M_{DPPS}) + (DP_{FDA} \times M_{FDA})} \times 100 \quad (S22)$$

$$f_{DPPS} = \frac{7.5 \times 288.32}{(7.5 \times 288.32) + (17.8 \times 518.17)} \times 100 = 19.0\ wt\%$$

$$f_{FDA} = 100 - f_{DPPS} = 100 - 19.0 \quad (S23)$$

$$f_{FDA} = 81.0\ wt\%$$

S2.5 FDA-StySAc copolymerization

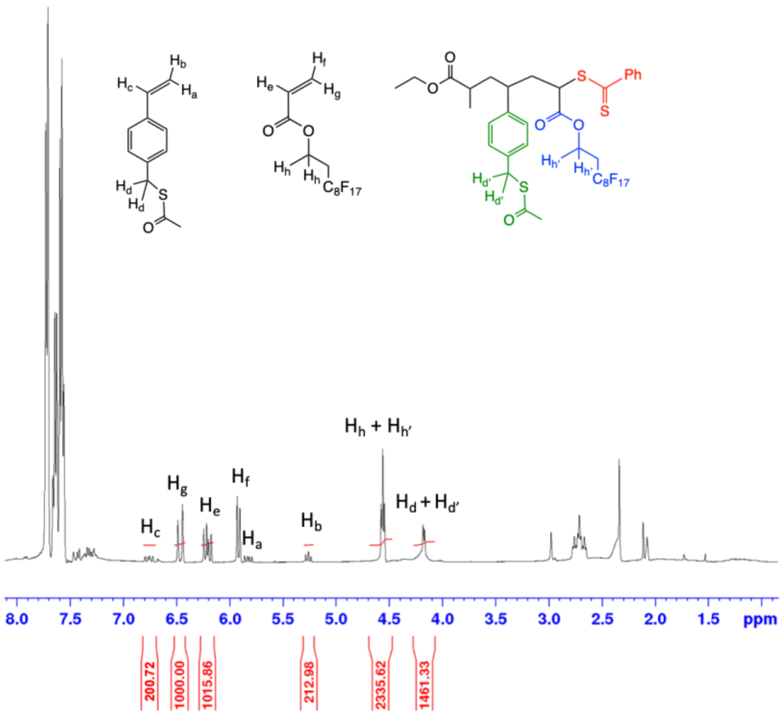

Figure S7. ^{1}H-NMR spectrum (400 MHz, TFT + C_6D_6 capillaries) of P(StySAc-*co*-FDA) at t = 48 h

Monomer conversions at t = 48 h:

$$\text{StySAc conversion (\%)} = \left(1 - \frac{(\mathrm{H_c} + \mathrm{H_b})/2}{(\mathrm{H_d} + \mathrm{H_{d'}})/2)}\right) \times 100 \qquad \text{(S24)}$$

StySAc conversion (%) = (1-(((201+213)/2)/(1461/2))) x 100

StySAc conversion (%) = 72%

$$\text{FDA conversion (\%)} = \left(1 - \frac{(\mathrm{H_e} + \mathrm{H_g})/2}{(\mathrm{H_h} + \mathrm{H_{h'}})/2)}\right) \times 100 \qquad \text{(S25)}$$

FDA conversion (%) = (1-(((1000+1016)/2)/(2336/2))) x 100

FDA conversion (%) = 14%

Degrees of polymerization and molecular weight of the final copolymer:

$$\mathrm{DP_{StySAc}} = \frac{(\mathrm{H_e} + \mathrm{H_f})/4}{(\mathrm{H_a} + \mathrm{H_b} + \mathrm{H_c})/5} = 9.7 \qquad \text{(S26)}$$

$$DP_{FDA} = \frac{((H_h + H_d) - 2\left(\frac{(H_e + H_f)}{4}\right))/2}{(H_a + H_b + H_c)/5} = 14.8 \quad (S27)$$

$$M_{n,precipitated\ P(StySAc-co-FDA)}\ (g/mol) = DP_{StySAc} \times M_{StySAc} + DP_{FDA} \times M_{FDA} + M_{CTA} = 9790\ g/mol \quad (S28)$$

where M_{StySAc}, M_{FDA} and M_{CTA} refer to the molecular weight of StySAc (192.28 g/mol), FDA (518.17 g/mol) and CTA (254.36 g/mol).

Weight fractions of StySAc complexing monomer units and FDA monomer units in final copolymer:

$$f_{StySAc} = \frac{DP_{StySAc} \times M_{StySAc}}{(DP_{StySAc} \times M_{StySAc}) + (DP_{FDA} \times M_{FDA})} \times 100 \quad (S29)$$

$$f_{StySAc} = \frac{9.7 \times 192.28}{(9.7 \times 192.28) + (14.8 \times 518.17)} \times 100 = 19.6\ wt\%$$

$$f_{FDA} = 100 - f_{StySAc} = 100 - 19.6 \quad (S30)$$

$$f_{FDA} = 80.4\ wt\%$$

S2.6 MALDI-TOF analyses

MALDI-TOF-MS mass spectrum of P(FDA) homopolymer was detected between 2000 and 8500 *m/z* (Figure S8).

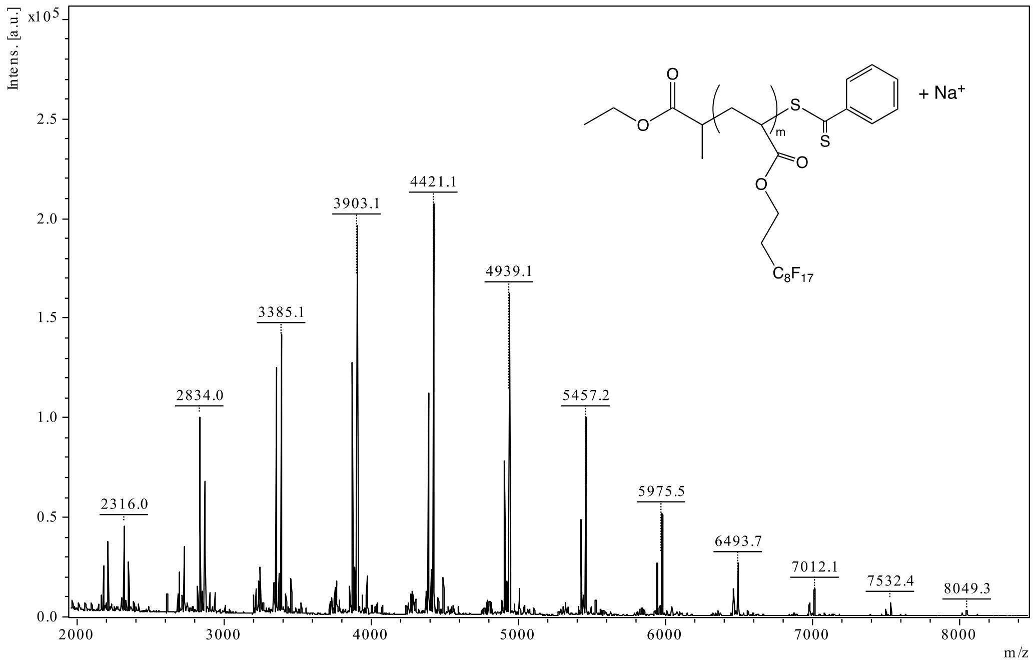

Figure S8. MALDI-TOF-MS mass spectrum in positive ion mode of P(FDA) homopolymer with DCTB as matrix and sodium trifluoroacetate as cationizing agent

Two populations are clearly visible and could be identified as illustrated in the enlarged MALDI-TOF mass spectrum (4350-5500 *m/z*, (Figure S9)). The major population at $(m/z)_{expt}$ = 4421.1, 4939.1, 5457.2 etc, corresponds to sodium ionized species $CH_3CH_2OCOCHCH_3(FDA_m)SCSC_6H_5$ + Na^+, i.e., the expected homopolymer incremented with one sodium cation. The number of repeating units of FDA (m) present in the polymer was determined by comparing the experimental mass/charge ($(m/z)_{expt}$) values with the calculated monoisotopic mass/charge ($(m/z)_{calc}$) values (Equation S31):

$$(m/z)_{calc} = m_{FDA} \times M_{FDA} + M_{CTA} + M_{Na} \quad \text{(S31)}$$

where M_{FDA} = 518.02 *m/z*, M_{CTA} = 254.04 *m/z* and M_{Na} = 22.99 *m/z*

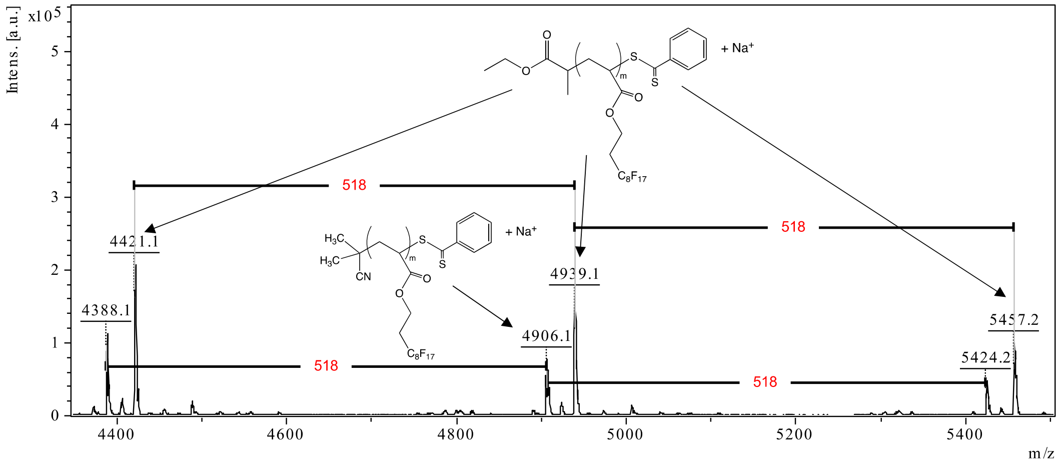

Figure S9. Enlarged MALDI-TOF-MS mass spectrum in positive ion mode of P(FDA) homopolymer (zoom between 4350 and 5500 *m/z*).

The second population at $(m/z)_{expt}$ = 4388.1, 4906.1, 5424.2, etc. could originate from P(FDA) homopolymer initiated by AIBN and terminated by the CTA end-group. For instance, the peak at $(m/z)_{expt}$ = 4906.1 corresponds to 9 units of FDA capped by AIBN and CTA in α and ω chain position, respectively, and cationized with sodium (i.e., $(CH_3)_2C(CN)(FDA)_9SCSC_6H_5 + Na^+$); $(m/z)_{calc}$ = 4906.2, according to Equation S32:

$$(m/z)_{calc} = m_{FDA} \times M_{FDA} + M_{(CH3)2C(CN)\ starting\ group} + M_{SCSC6H5\ end\ group} + M_{Na} \quad (S32)$$

where M_{FDA} = 518.02 *m/z*, $M_{(CH3)2C(CN)\ starting\ group}$ = 68.06 *m/z*, $M_{SCSC6H5\ end\ group}$ = 152.98 *m/z* and M_{Na} = 22.99 *m/z*

Polymer chains initiated by AIBN may exist according to the RAFT polymerization mechanism, but in very small amount. However, it is noteworthy that MALDI-TOF analyses are not quantitative and AIBN initiated polymer chains may be better detected than the expected P(FDA).

For both populations, the differences in the $(m/z)_{expt}$ series of peaks is 518 (i.e., M_{FDA}).

MALDI-TOF-MS mass spectrum of P(4VP-*co*-FDA) copolymer was detected between 1400 and 8400 *m/z* (Figure S10).

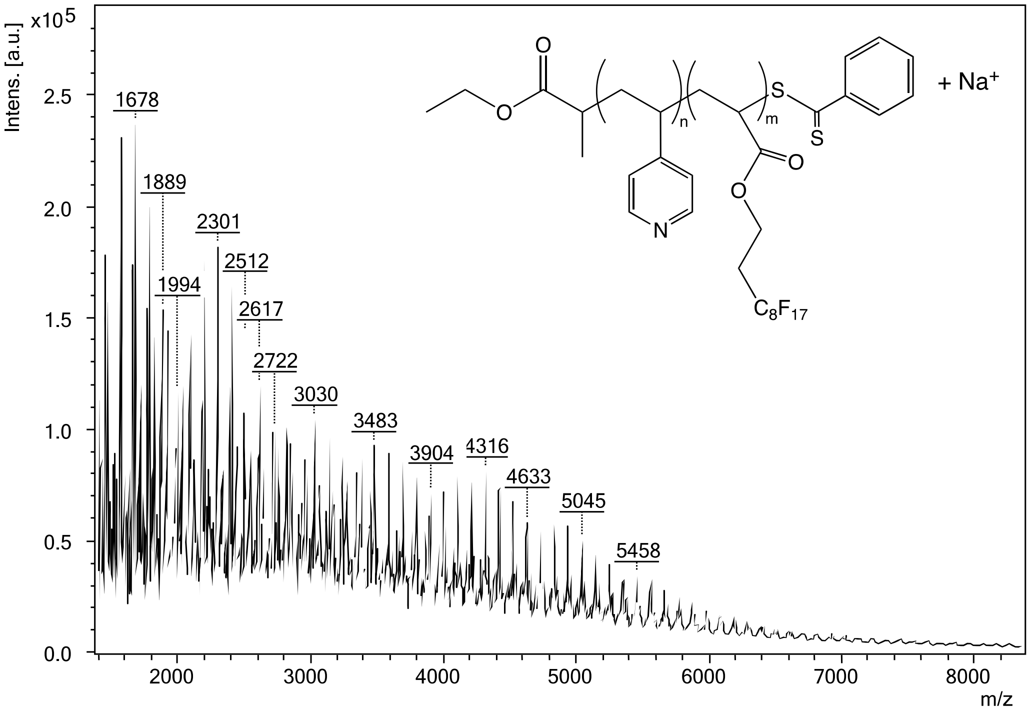

Figure S10. MALDI-TOF-MS mass spectrum in positive ion mode of P(4VP-*co*-FDA) with DCTB as matrix and sodium trifluoroacetate as cationizing agent

Enlargement of the spectrum from 3460 to 4040 *m/z* (Figure S11) revealed the presence of main peaks corresponding to protonated adducts with formula $CH_3CH_2OCOCHCH_3(4VP_n$-*co*-$FDA_m)SCSC_6H_5 + H^+$, i.e. one CTA end-group associated with n units of 4VP and m units of FDA monomer incremented with one proton due to ionization. These adducts are associated with species ionized with sodium with formula $CH_3CH_2OCOCHCH_3(4VP_n$-*co*-$FDA_m)SCSC_6H_5 + Na^+$.

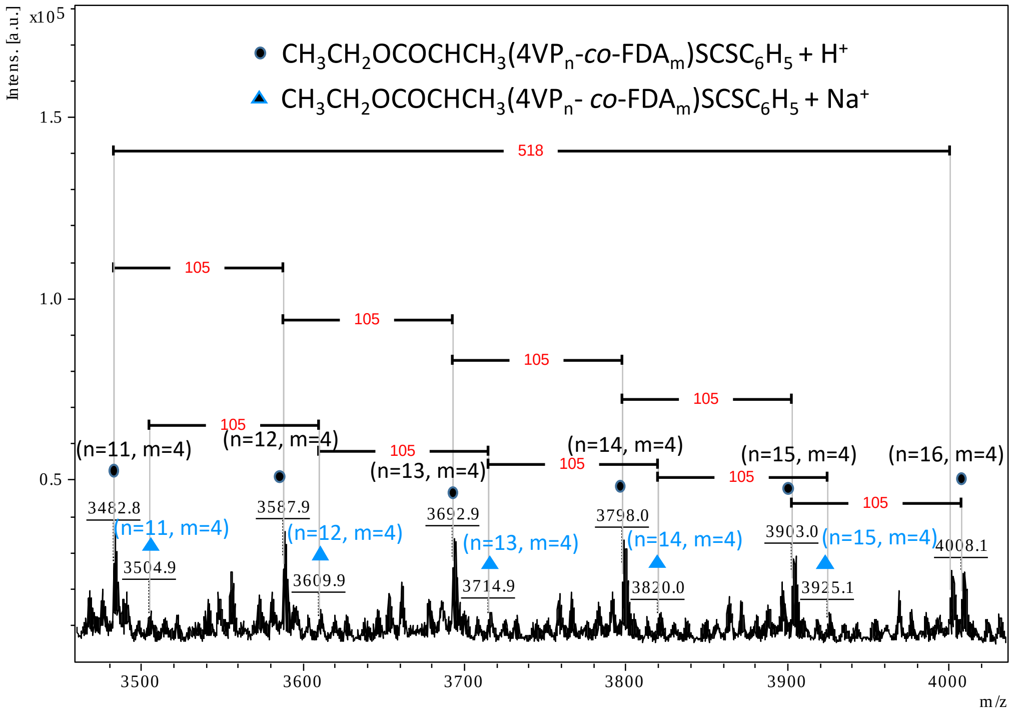

Figure S11. Enlarged MALDI-TOF-MS mass spectrum in positive ion mode of P(4VP-*co*-FDA) (zoom between 3460 to 4040 *m/z*).

The number of repeating units of 4VP (n) and FDA (m) present in the copolymer was determined by comparing the $(m/z)_{expt}$ values with the calculated monoisotopic $(m/z)_{calc}$ values (Equation S33 and Equation S34):

$$(m/z)_{calc} = n_{4VP} \times M_{4VP} + m_{FDA} \times M_{FDA} + M_{CTA} + M_{H} \tag{S33}$$

$$(m/z)_{calc} = n_{4VP} \times M_{4VP} + m_{FDA} \times M_{FDA} + M_{CTA} + M_{Na} \tag{S34}$$

where M_{4VP} = 105.06 *m/z*, M_{FDA} = 518.02 *m/z*, M_{CTA} = 254.04 *m/z*, M_{H} = 1.01 *m/z* and M_{Na} = 22.99 *m/z*

Besides, the MALDI-TOF-MS spectrum exhibits the presence of peaks spaced by one FDA unit (i.e., 518.02 *m/z*) together with the presence of series of peaks with a difference in the $(m/z)_{expt}$ of 105 (M_{4VP} = 105.06 *m/z*). All the $(m/z)_{expt}$ values for these series of peaks were in good correlation with the calculated $(m/z)_{calc}$ values (Table S1). Note that the proton affinity of poly(4-vinylpyridine) introduced by the numerous pyridyl groups (4VP) leads to the almost exclusive formation of $[M + H]^{+}$ ions in the ionization source despite the presence of the non-acidic and aprotic matrices such as DCTB as in the case of poly(2-vinylpyridine) [Gross J. H. Poly(2-vinylpyridine) as a reference compound for mass calibration in positive-ion matrix-assisted laser desorption/ionization-mass spectrometry on different instrumental platforms. *Eur. J. Mass Spectrom.* **2021**, *27*, 191-204], implying a peak intensity of the protonated compounds higher than that of the sodium adducts (Figure S11).

Table S1. Assignments of peaks in P(4VP-*co*-FDA) copolymer, with n 4VP units and m FDA units

(a) ● $CH_3CH_2OCOCHCH_3(4VP_n\text{-}co\text{-}FDA_m)SCSC_6H_5 + H^+$

$(m/z)_{expt}$	n	m	$(m/z)_{calc}$ [a]	$\Delta = \lvert (m/z)_{expt} - (m/z)_{calc} \rvert$
3482.6	11	4	3482.8	0.2
3587.7	12	4	3587.8	0.1
3692.7	13	4	3692.9	0.2
3797.8	14	4	3797.9	0.1
3902.8	15	4	3903	0.2
4000.6	11	5	4000.8	0.2
4007.9	16	4	4008	0.1

(b) ▲ $CH_3CH_2OCOCHCH_3(4VP_n\text{-}co\text{-}FDA_m)SCSC_6H_5 + Na^+$

$(m/z)_{expt}$	n	m	$(m/z)_{calc}$ [a]	$\Delta = \lvert (m/z)_{expt} - (m/z)_{calc} \rvert$
3497.5	6	5	3497.6	0.1
3504.6	11	4	3504.7	0.1
3602.7	7	5	3602.5	0.2
3609.7	12	4	3609.8	0.1
3707.7	8	5	3707.6	0.1
3714.7	13	4	3714.9	0.2
3805.5	4	6	3805.4	0.1
3820	14	4	3819.9	0.1
3910	5	6	3910.4	0.4
3925.1	15	4	3925	0.1

[a] Calculated from the monoisotopic mass.

Lastly, the isotope patterns of theoretical and experimental protonated adducts of P(4VP-*co*-FDA) copolymer exhibit a similar shape and are therefore in good agreement (Figure S12).

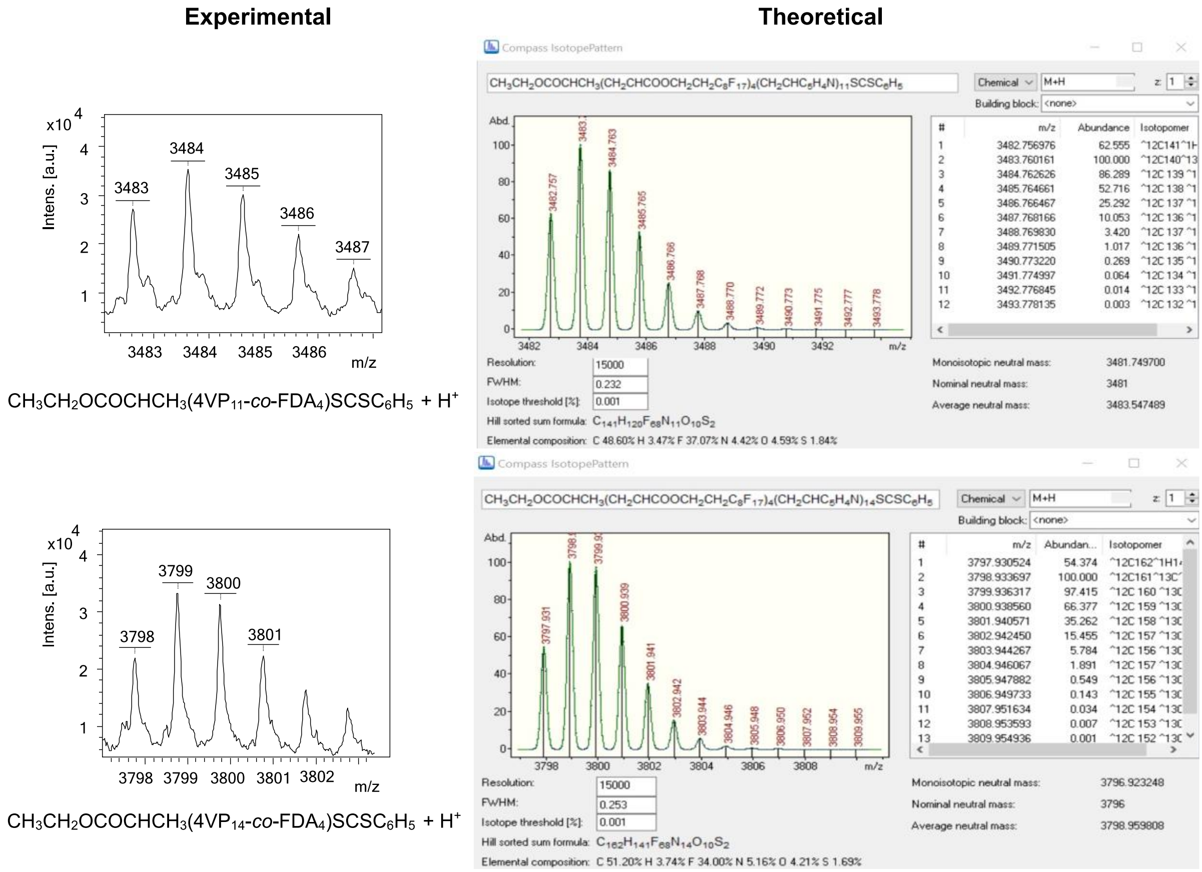

Figure S12. Experimental and theoretical isotope patterns of proton adducts of $CH_3CH_2OCOCHCH_3(4VP_n\text{-}co\text{-}FDA_m)SCSC_6H_5$

Hence, the MALDI-TOF spectrum confirms the formation of the P(4VP-*co*-FDA) copolymer.

Dithranol was used as matrix and sodium iodide (NaI) as a cationizing agent to perform the MALDI-TOF-MS mass spectrum of P(DPPS-*co*-FDA) copolymer shown from 1500 to 6000 *m/z* (Figure S13).

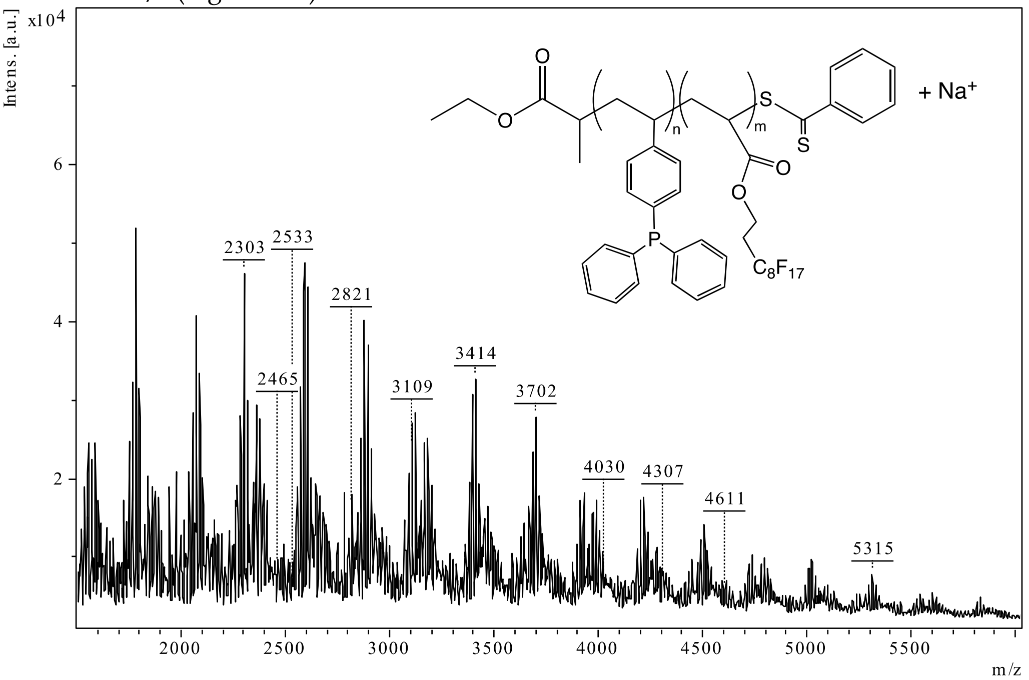

Figure S13. MALDI-TOF-MS mass spectrum in positive ion mode of P(DPPS-*co*-FDA) with dithranol as matrix and NaI as cationizing agent

Enlargement of the MALDI-TOF-MS mass spectrum (2960 – 3780 *m/z*, Figure S14) revealed the presence of peaks arising from the species ionized with sodium with formula $CH_3CH_2OCOCHCH_3(DPPS_n\text{-}co\text{-}FDA_m)SCSC_6H_5 + Na^+$, i.e. one CTA end-group associated with some units of DPPS and some units of FDA monomer incremented with one sodium cation due to ionization. The number of repeating units of DPPS (n) and FDA (m) present in the copolymer was determined by comparing the $(m/z)_{expt}$ values with the calculated monoisotopic $(m/z)_{calc}$ values according to Equation S35:

$$(m/z)_{calc} = n_{DPPS} \times M_{DPPS} + m_{FDA} \times M_{FDA} + M_{CTA} + M_{Na} \qquad (S35)$$

where M_{DPPS} = 288.11 *m/z*, M_{FDA} = 518.02 *m/z*, M_{CTA} = 254.04 *m/z* and M_{Na} = 22.99 *m/z*

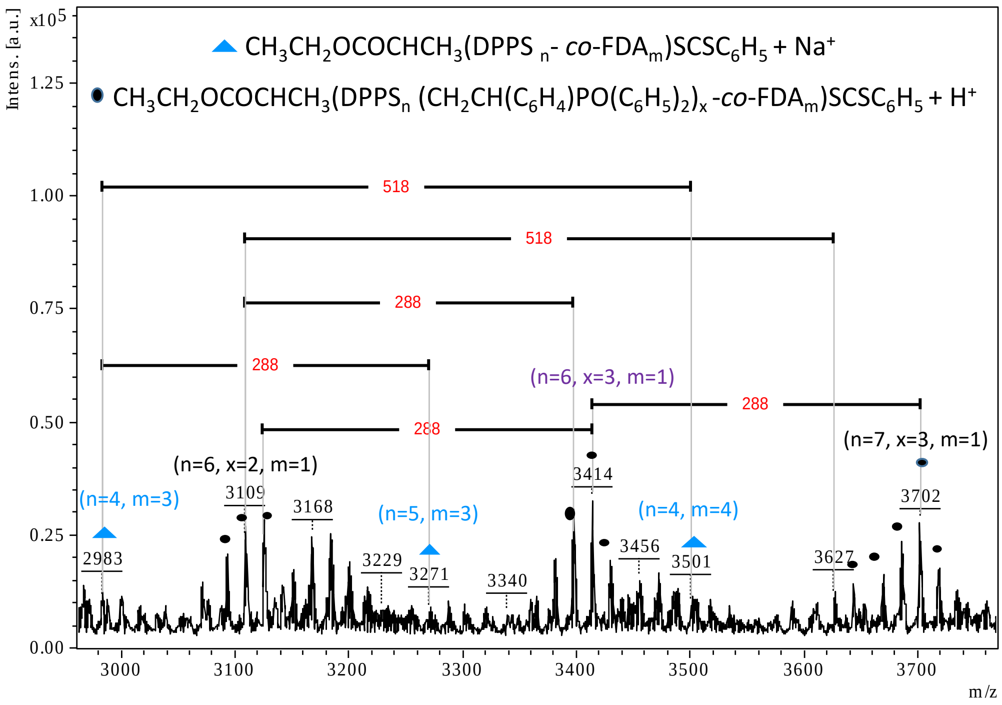

Figure S14. Enlarged MALDI-TOF-MS mass spectrum in positive ion mode of P(DPPS-*co*-FDA) (zoom between 2960 to 3780 *m/z*).

The MALDI-TOF-MS spectrum also exhibits the presence of peaks spaced by one FDA unit (i.e., 518.02 *m/z*) together with series of peaks with a difference in the $(m/z)_{expt}$ corresponding to one DPPS unit (288.11 *m/z*). A good correlation exists between the $(m/z)_{expt}$ and the calculated $(m/z)_{calc}$ values for these series of peaks (
Table S2).

Table S2. Assignments of peaks in P(DPPS-*co*-FDA), with n DPPS units and m FDA units

$(m/z)_{expt}$	n	m	$(m/z)_{calc}$ [a]	$\Delta = \mid (m/z)_{expt} - (m/z)_{calc} \mid$
2983.2	4	3	2983.5	0.2
3271.2	5	3	3271.5	0.3
3501.1	4	4	3501.5	0.4

[a] Calculated from the monoisotopic mass.

However, another population at + 16 *m/z* was also observed (Figure S14 and Figure S15). This mass difference corresponding to an additional oxygen atom, this population was attributed to an oxidation of some DPPS units. Mixture of oxidized and non-oxidized DPPS units being present in a same molecule according to formula $CH_3CH_2OCOCHCH_3(DPPS_n$-*co*-$(CH_2CH(C_6H_4)PO(C_6H_5)_2)_x$-*co*-$FDA_m)SCSC_6H_5 + H^+$, i.e. one CTA end-group associated with some units of DPPS, some units of oxidized DPPS and some units of FDA monomer incremented with one proton to ionization. The number of repeating units of DPPS (n), oxidized DPPS (x) and FDA (m) present in the copolymer was determined by comparing the $(m/z)_{expt}$ values with the calculated monoisotopic $(m/z)_{calc}$ values (Equation S36):

$$(m/z)_{calc} = n_{DPPS} \times M_{DPPS} + x_{oxidized\ DPPS} \times M_{oxidized\ DPPS} + m_{FDA} \times M_{FDA} + M_{CTA} + M_H \quad (S36)$$

where M_{DPPS} = 288.11 *m/z*, $M_{oxidized\ DPPS}$ = 304.10 *m/z*, M_{FDA} = 518.02 *m/z*, M_{CTA} = 254.04 *m/z* and M_H = 1.01 *m/z*

- $CH_3CH_2OCOCHCH_3(DPPS_n\ (CH_2CH(C_6H_4)PO(C_6H_5)_2)_x$ -*co*-$FDA_m)SCSC_6H_5 + H^+$

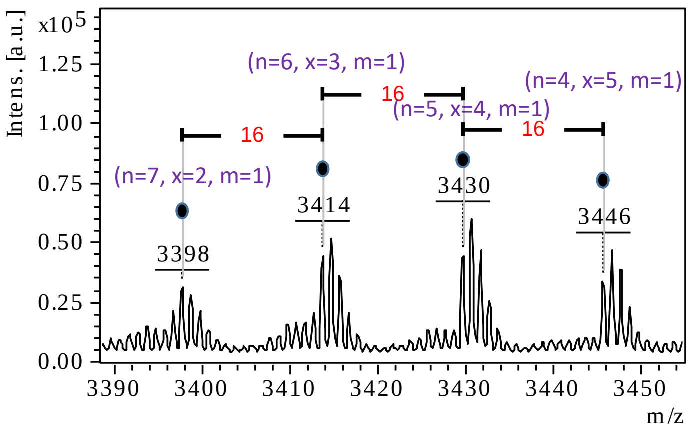

Figure S15. Zoom between 3388 and 3455 *m/z* of MALDI-TOF-MS mass spectrum in positive ion mode of P(DPPS-*co*-oxidized DPPS-*co*-FDA) copolymer to evidence the mass difference of +16 *m/z*.

This mixture of oxidized and non-oxidized DPPS units is confirmed (Table S3) where the experimental values $(m/z)_{expt}$ were in good correlation with the calculated values $(m/z)_{calc}$. Note that molecules containing oxidized DPPS were detected as protonated adducts whereas molecules with non-oxidized DPPS were detected as sodium adducts. MALDI analyses make protonated adducts the majority. It is noteworthy that phosphine is a reducing compound and oxidation of reducing compound (such as thiol) has already been described in the literature [Gaillot C.; Delolme F.; Fabre L.; Charreyre M.-T.; Ladavie□re C.; Favier A. Taking advantage of oxidation to characterize thiol-containing polymer chains by MALDI-TOF mass spectrometry. *Anal. Chem.* **2020**, *92*, 3804−3809].

Table S3. Assignments of peaks in P(DPPS-*co*-oxidized DPPS-*co*-FDA), with n DPPS units, x oxidized DPPS units and m FDA units

$(m/z)_{expt}$	n	x	m	$(m/z)_{calc}$ [a]	Δ = I $(m/z)_{expt}$ - $(m/z)_{calc}$ I
3109.4	6	2	1	3109.9	0.5
3126	5	3	1	3125.9	0.1
3398.1[b]	7	2	1	3398	0.1
3413.5[b]	6	3	1	3414	0.5
3430.1[b]	5	4	1	3430	0.1
3445.8[b]	4	5	1	3446	0.2
3670	9	1	1	3670.1	0.1
3686	8	2	1	3686.1	0.1
3702	7	3	1	3702.1	0.5

[a] Calculated from the monoisotopic mass.
[b] $(m/z)_{expt}$ referenced in Figure S15

The MALDI-TOF-MS mass spectrum obtained from the P(StySAc-*co*-FDA) copolymer is presented from 2900 to 9700 *m/z* (Figure S16).

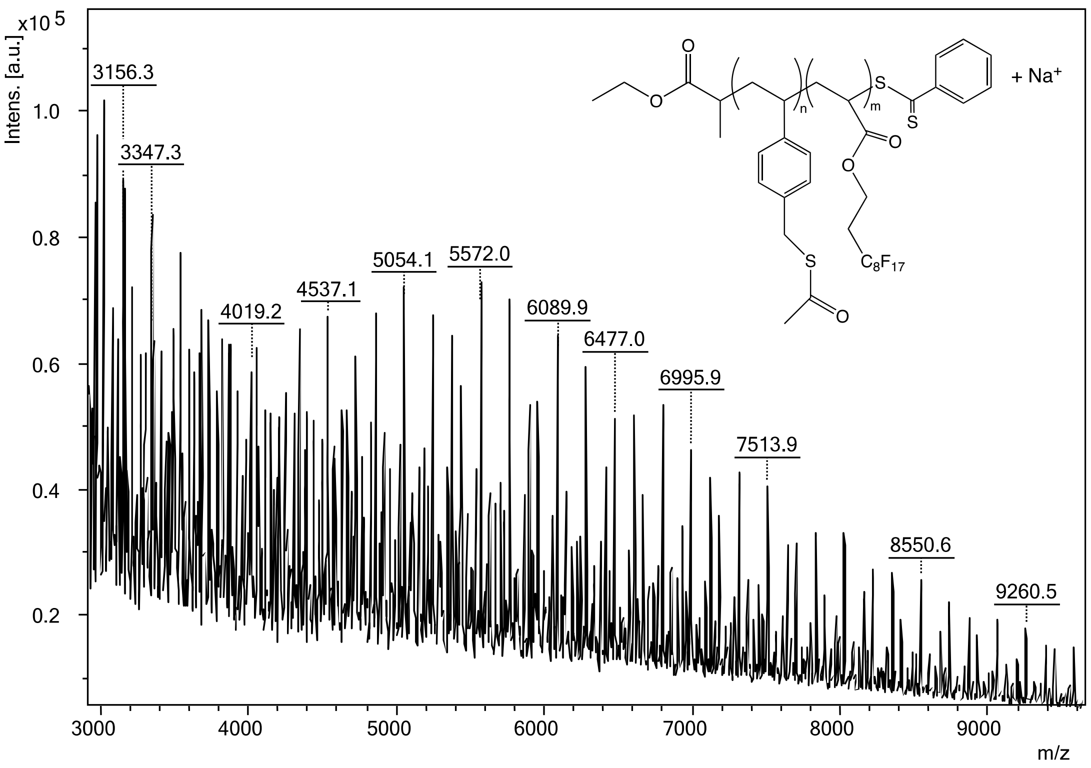

Figure S16. MALDI-TOF-MS mass spectrum in positive ion mode of P(StySAc-*co*-FDA) with DCTB as matrix and sodium trifluoroacetate as cationizing agent

A more detailed analysis of the MALDI-TOF data in the region from 5560 to 6180 *m/z* (Figure S17) shows that the main peaks arise from the species ionized with sodium with formula $CH_3CH_2OCOCHCH_3(StySAc_n\text{-}co\text{-}FDA_m)SCSC_6H_5 + Na^+$, i.e. one CTA end-group associated with n units of StySAc and m units of FDA monomer incremented with one sodium cation due to ionization. The number of repeating units of StySAc (n) and FDA (m) present in the copolymer was determined by comparing the $(m/z)_{expt}$ values with the calculated monoisotopic $(m/z)_{calc}$ values (Equation S37):

$$(m/z)_{calc} = n_{StySAc} \times M_{StySAc} + m_{FDA} \times M_{FDA} + M_{CTA} + M_{Na} \quad \text{(S37)}$$

where M_{StySAc} = 192.06 *m/z*, M_{FDA} = 518.02 *m/z*, M_{CTA} = 254.04 *m/z* and M_{Na} = 22.99 *m/z*

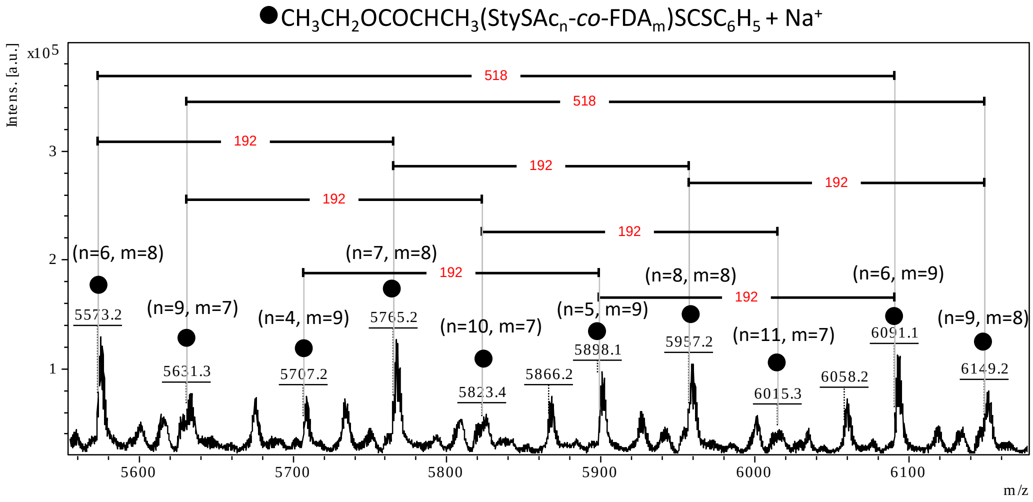

Figure S17. Enlarged MALDI-TOF-MS mass spectrum in positive ion mode of P(StySAc-*co*-FDA) (zoom between 5560 to 6180 *m/z*).

The expected values $(m/z)_{expt}$ were in good correlation with the calculated $(m/z)_{calc}$ values (Table S4).

Table S4. Assignments of peaks in P(StySAc-*co*-FDA), with n StySAc units and m FDA units

$(m/z)_{expt}$	n	m	$(m/z)_{calc}$ [a]	$\Delta = \lvert (m/z)_{expt} - (m/z)_{calc} \rvert$
5573.2	6	8	5573.5	0.3
5631.3	9	7	5631.7	0.4
5707.2	4	9	5707.4	0.2
5765.2	7	8	5765.5	0.3
5823.3	10	7	5823.7	0.4
5899.1	5	9	5899.4	0.3
5957.2	8	8	5957.6	0.4
6015.3	11	7	6015.8	0.5
6091.1	6	9	6091.5	0.4
6149.2	9	8	6149.7	0.5

[a] Calculated from the monoisotopic mass.

Lastly, the isotope patterns of theoretical and experimental sodium adducts of P(StySAc-*co*-FDA) copolymer exhibit a similar shape and are therefore in good agreement (Figure S18). The formation of the P(StySAc-*co*-FDA) copolymer is therefore well confirmed.

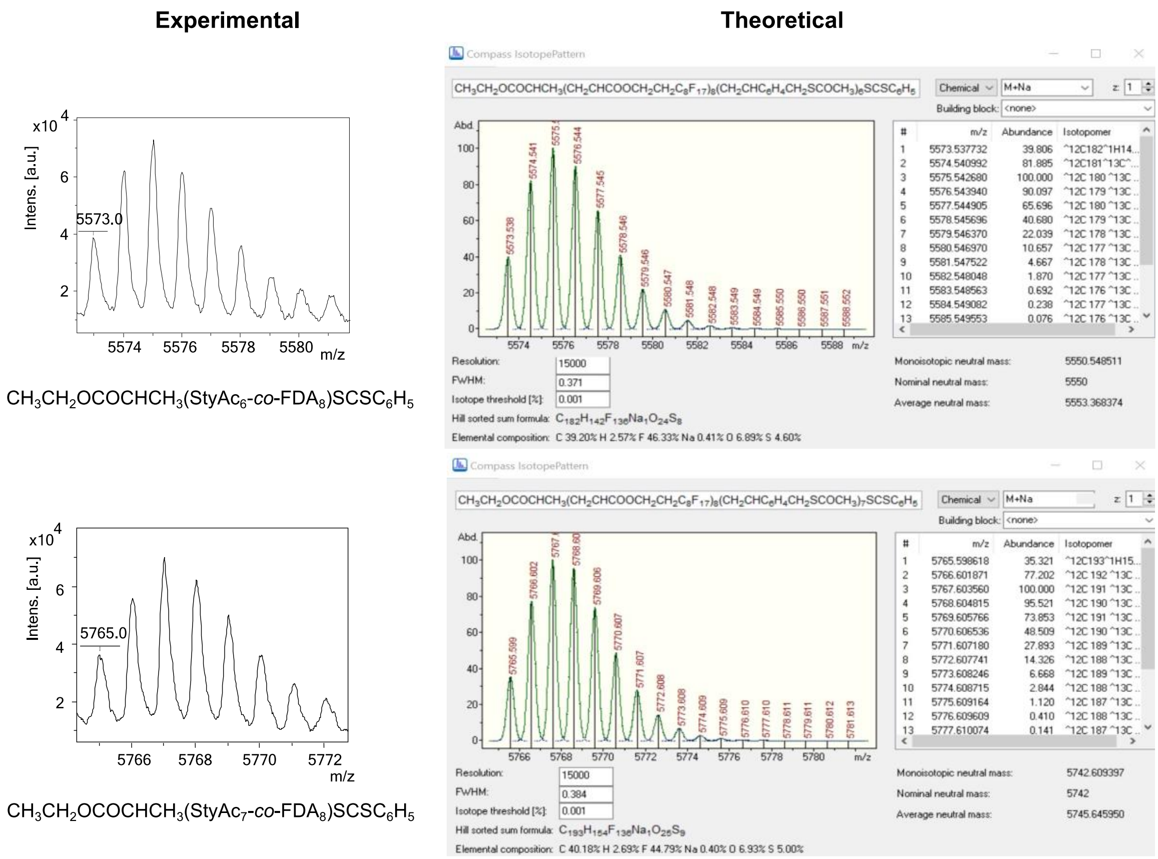

Figure S18. Experimental and theoretical isotope patterns of sodium adducts of $CH_3CH_2OCOCHCH_3(StySAc_n\text{-}co\text{-}FDA_m)SCSC_6H_5$

S3. Polymer after aminolysis

The molar ratio between monomers was compared before and after aminolysis to be sure that the composition of the copolymers was not affected by the reaction and that no fractionation occurs during polymer precipitation/purification.

S3.1 P(FDA)SH

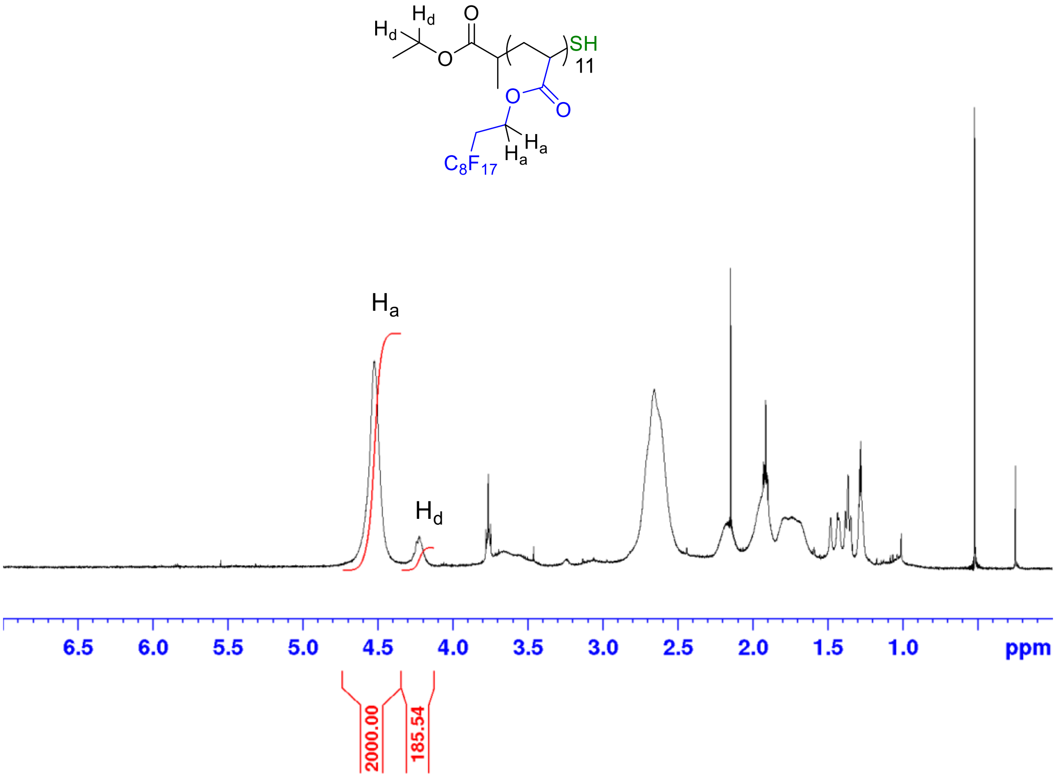

Figure S19. ^{1}H-NMR spectrum (400 MHz, CFC-113 + C_6D_6 capillaries) of P(FDA)SH after precipitation.

S3.2 P(4VP-co-FDA)SH

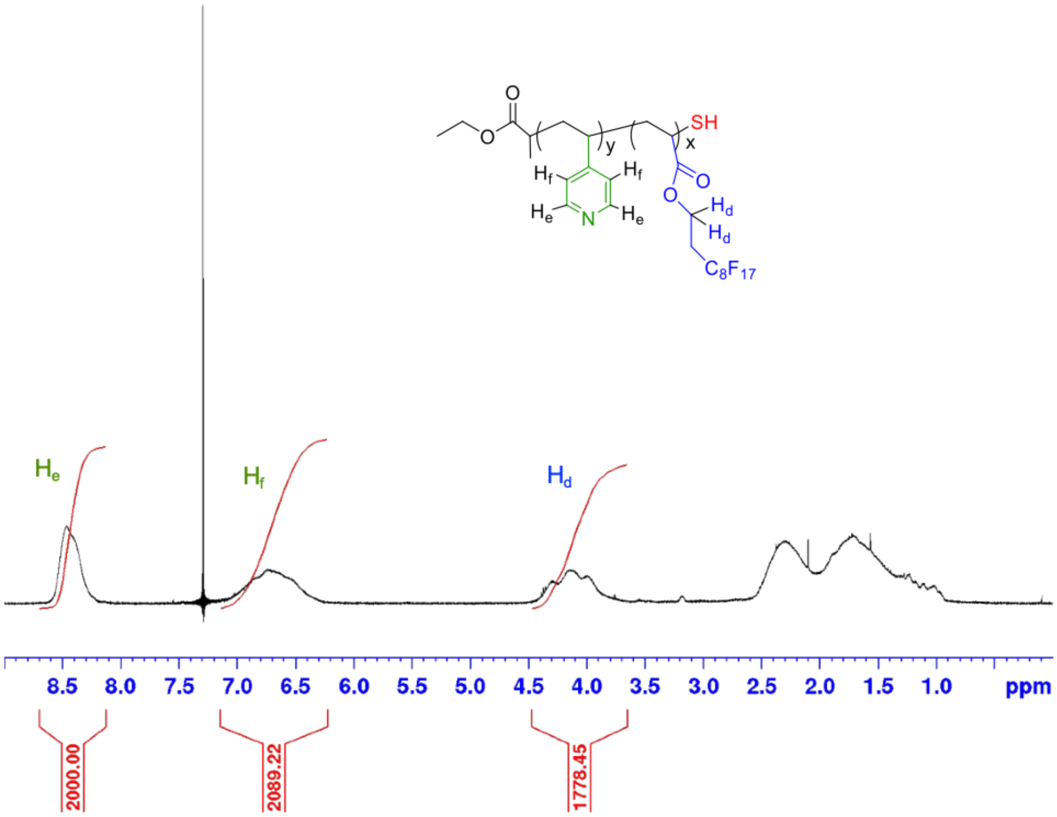

Figure S20. ^{1}H-NMR spectrum (400 MHz, $CDCl_3$) of P(4VP-*co*-FDA)SH after precipitation.

Before aminolysis (Figure 3):

$$[4VP] = \frac{H_e + H_f}{4} = \frac{41350 + 44207}{4} = 21389$$

$$[FDA] = \frac{H_d}{2} = \frac{39500}{2} = 19750$$

$$\text{so } \frac{[4VP]}{[FDA]} = 1.08$$

After aminolysis (Figure S20):

$$[4VP] = \frac{H_e + H_f}{4} = \frac{2000 + 2089}{4} = 1022$$

$$[FDA] = \frac{H_d}{2} = \frac{1778}{2} = 889$$

$$\text{so } \frac{[4\text{VP}]}{[\text{FDA}]} = 1.15$$

S3.3 P(AAEM-co-FDA)SH (piperidine aminolysis)

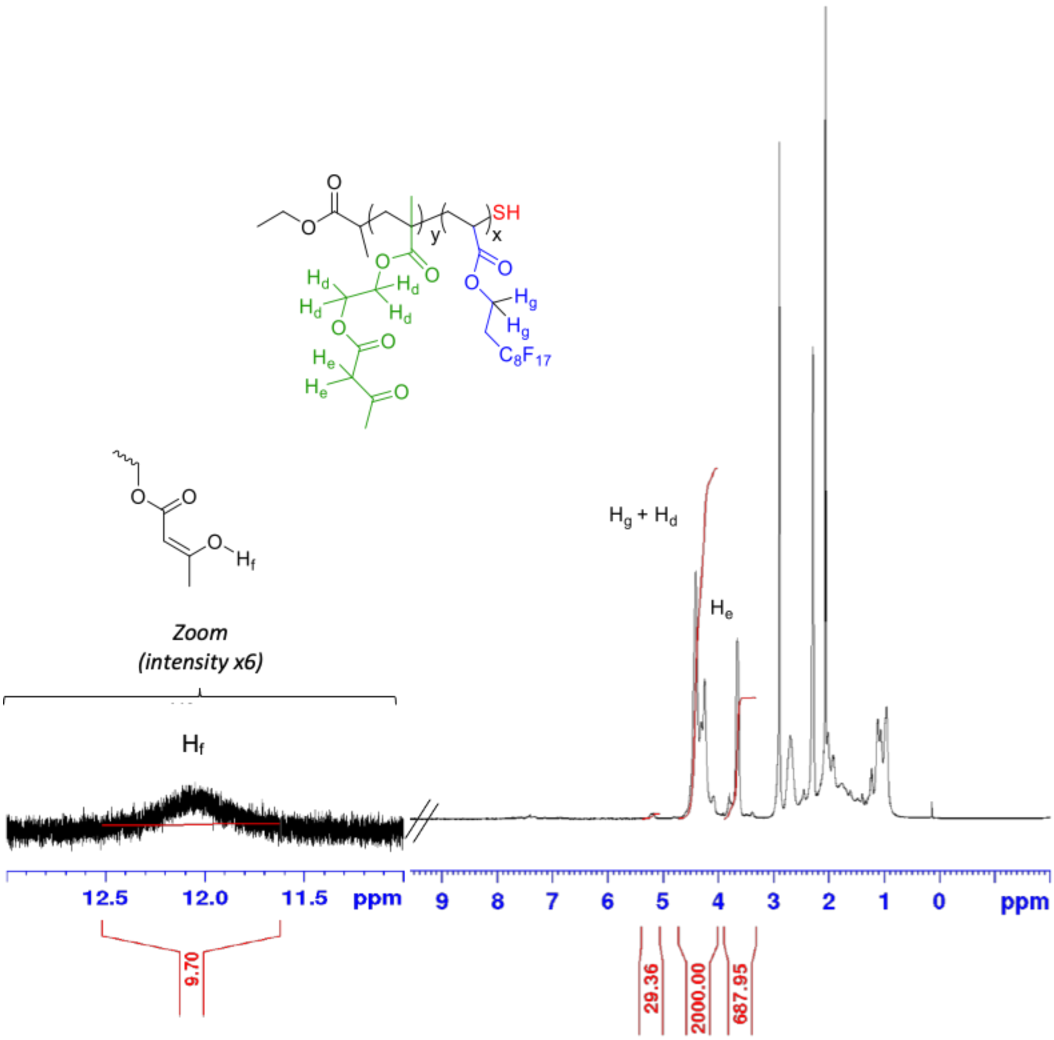

Figure S21. ^{1}H-NMR spectrum (400 MHz, acetone-d6) of P(AAEM-*co*-FDA)SH after precipitation, obtained by aminolysis with piperidine.

Before aminolysis (Figure 4):

$$[\text{AAEM}] = \frac{\text{H}_\text{f}}{1} + \frac{\text{H}_\text{e}}{2} = 986 + \frac{42581}{2} = 22276$$

$$[\text{FDA}] = \frac{(\text{H}_\text{g} + \text{H}_\text{d}) - 4(\text{H}_\text{f} + \frac{\text{H}_\text{e}}{2})}{2} = \frac{130853 - 4(986 + \frac{42581}{2})}{2} = 20873$$

$$\text{so } \frac{[\text{AAEM}]}{[\text{FDA}]} = 1.07$$

After aminolysis (Figure S21):

$$[\mathrm{AAEM}] = \frac{\mathrm{H_f}}{1} + \frac{\mathrm{H_e}}{2} = 9.70 + \frac{688}{2} = 354$$

$$[\mathrm{FDA}] = \frac{(\mathrm{H_g} + \mathrm{H_d}) - 4(\mathrm{H_f} + \frac{\mathrm{H_e}}{2})}{2} = \frac{2000 - 4(9.70 + \frac{688}{2})}{2} = 293$$

$$\text{so } \frac{[\mathrm{AAEM}]}{[\mathrm{FDA}]} = 1.2$$

S3.4 P(AAEM-co-FDA)SH (n-Butylamine aminolysis)

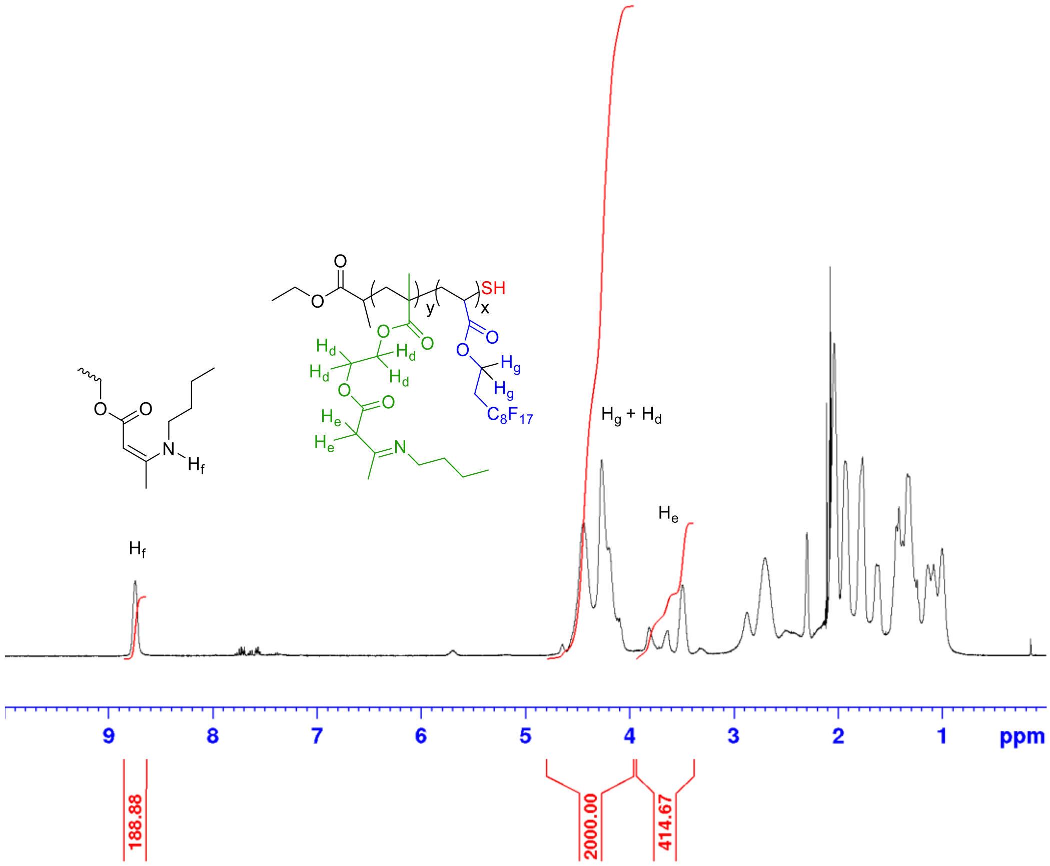

Figure S22. ^{1}H-NMR spectrum (400 MHz, acetone-d6) of P(AAEM-*co*-FDA)SH after precipitation, obtained by aminolysis reaction with n-butylamine.

After aminolysis (Figure S22):

$$[\mathrm{AAEM}] = \frac{\mathrm{H_f}}{1} + \frac{\mathrm{H_e}}{2} = 189 + \frac{415}{2} = 396$$

$$[\mathrm{FDA}] = \frac{(\mathrm{H_g} + \mathrm{H_d}) - 4(\mathrm{H_f} + \frac{\mathrm{H_e}}{2})}{2} = \frac{2000 - 4(189 + \frac{415}{2})}{2} = 207$$

$$\text{so } \frac{[\mathrm{AAEM}]}{[\mathrm{FDA}]} = 1.9$$

S3.5 P(DPPS-co-FDA)SH

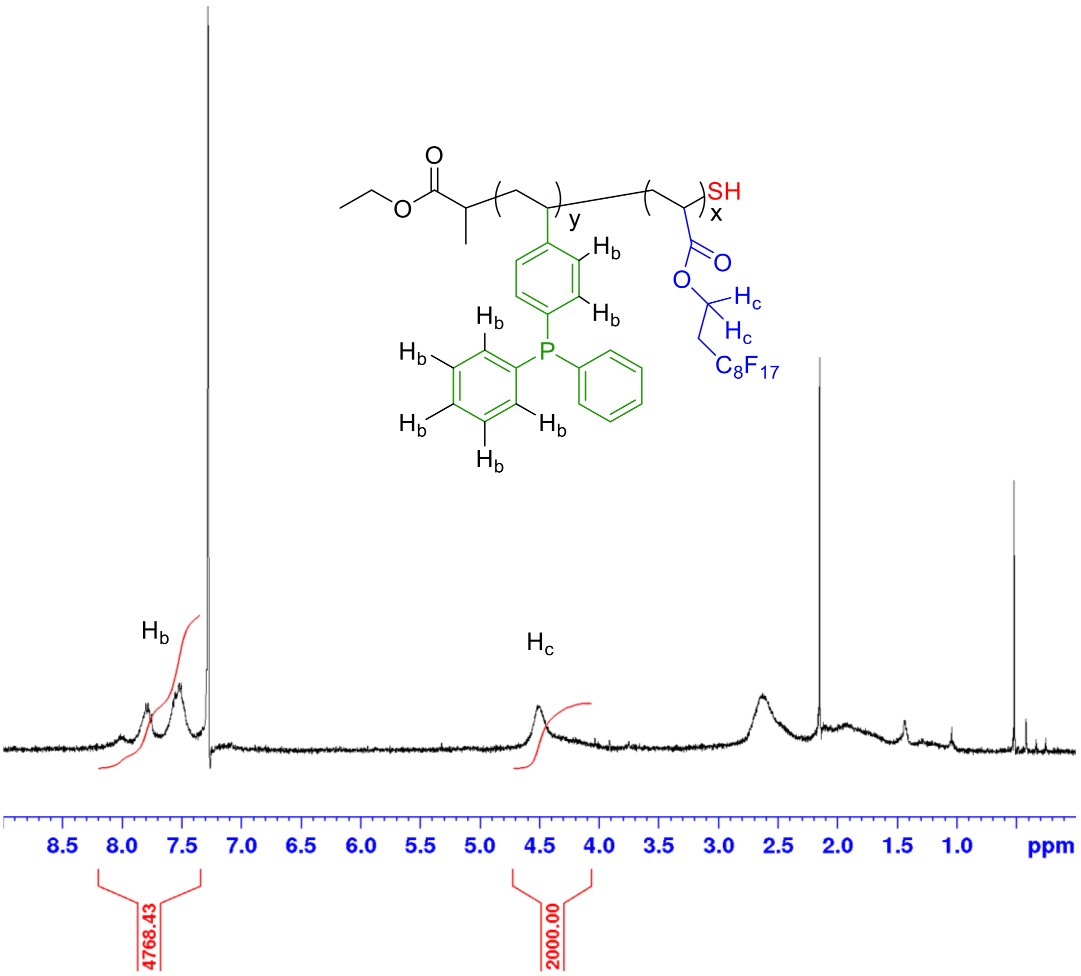

Figure S23. ^{1}H-NMR spectrum (400 MHz, CFC-113 + C_6D_6 capillaries) of P(DPPS-*co*-FDA)SH after precipitation.

Before aminolysis (Figure 5):

$$[\mathrm{DPPS}] = \frac{\mathrm{H_c}}{14} = \frac{181059}{14} = 12933$$

$$[\mathrm{FDA}] = \frac{\mathrm{H_d}}{2} = \frac{61544}{2} = 30772$$

$$so\ \frac{[\mathrm{DPPS}]}{[\mathrm{FDA}]} = 0.42$$

After aminolysis (Figure S23):

$$[\mathrm{DPPS}] = \frac{\mathrm{H_b}}{14} = \frac{4768}{14} = 341$$

$$[\mathrm{FDA}] = \frac{\mathrm{H_c}}{2} = \frac{2000}{2} = 1000$$

$$so\ \frac{[\mathrm{DPPS}]}{[\mathrm{FDA}]} = 0.34$$

S3.6 P(StySH-co-FDA)SH

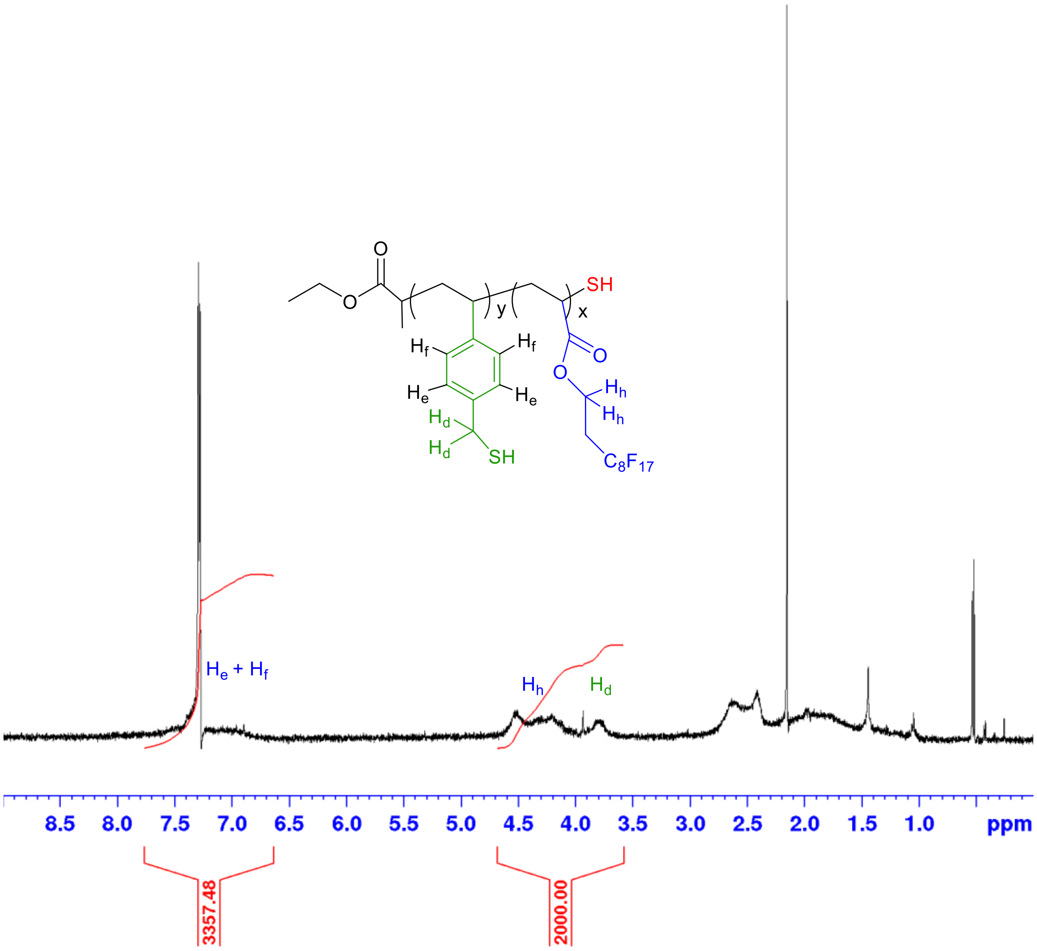

Figure S24. ^{1}H-NMR spectrum (400 MHz, $CDCl_3$) of P(StySH-*co*-FDA)SH after precipitation.

Before aminolysis (Figure 6):

$$[\mathrm{StySAc}] = \frac{(\mathrm{H_e} + \mathrm{H_f})}{4} = \frac{46803}{4} = 11701$$

$$[\mathrm{FDA}] = \frac{(\mathrm{H_h} + \mathrm{H_d}) - 2(\frac{\mathrm{H_e} + \mathrm{H_f}}{4})}{2} = \frac{58876 - 2(\frac{46803}{4})}{2} = 17737$$

$$\text{so } \frac{[\mathrm{StySAc}]}{[\mathrm{FDA}]} = 0.66$$

After aminolysis (Figure S24):

$$\frac{[\mathrm{StySH}]}{[\mathrm{FDA}]} = \text{not calculated, too much deuterated solvent}$$

S4. Thermal characterization of the polymers

S4.1 P(FDA)

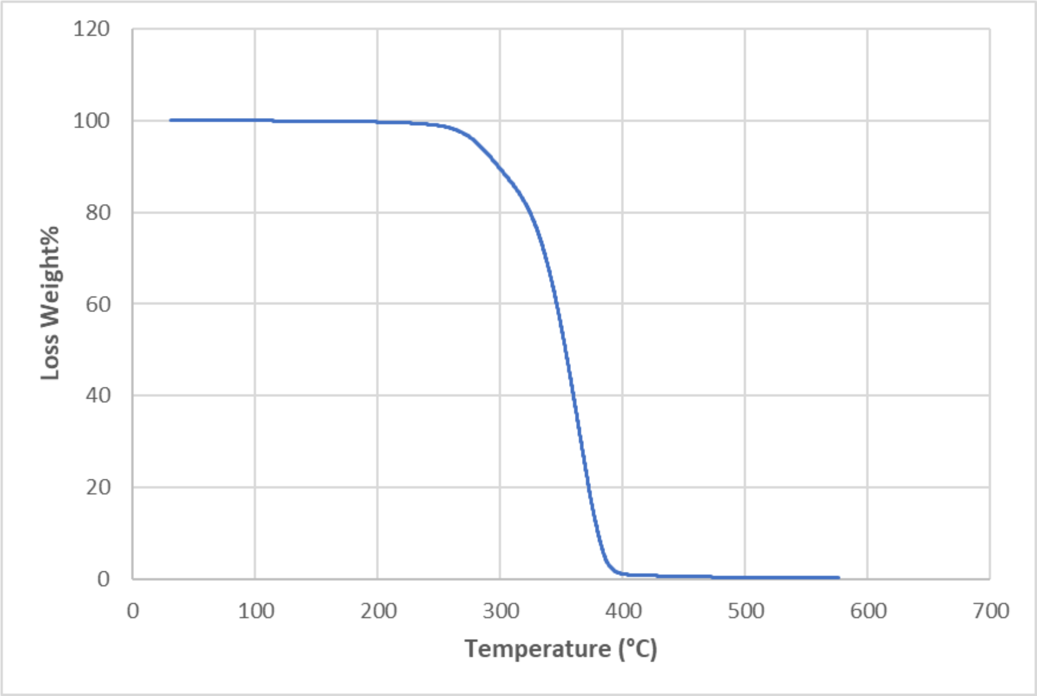

Figure S25. TGA measurement of P(FDA).

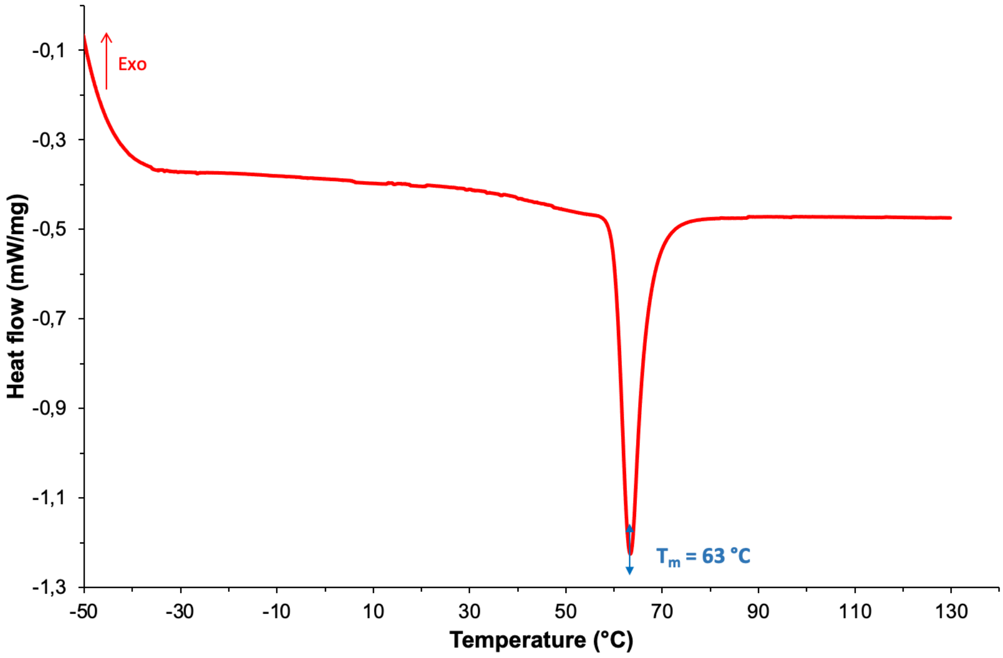

Figure S26. DSC measurement of P(FDA**)**

S4.2 P(FDA)SH

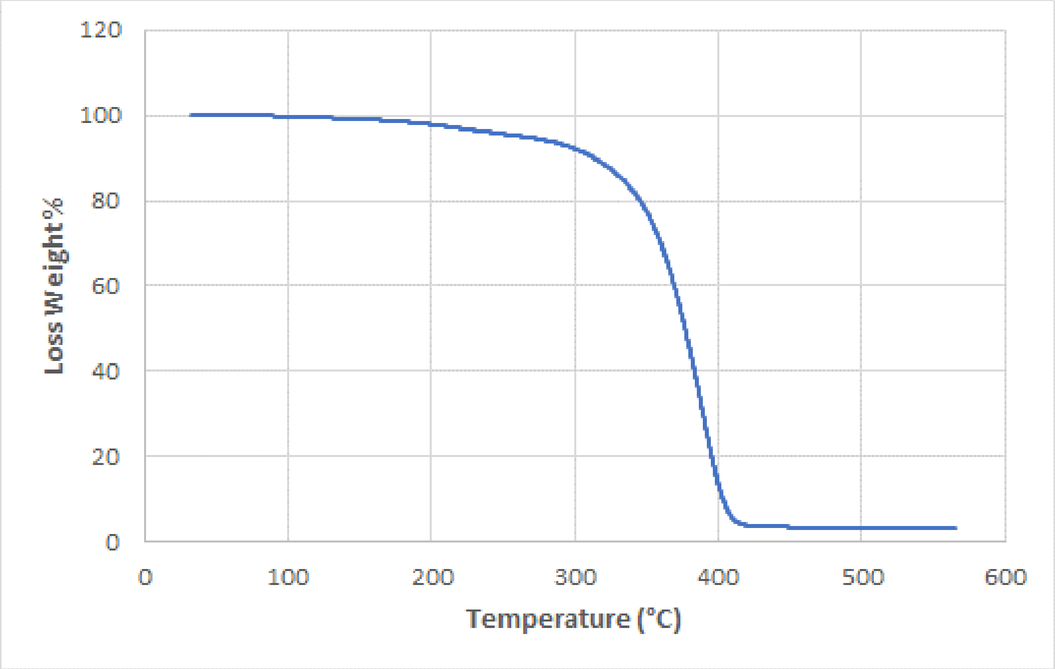

Figure S27. TGA measurement of P(FDA)SH.

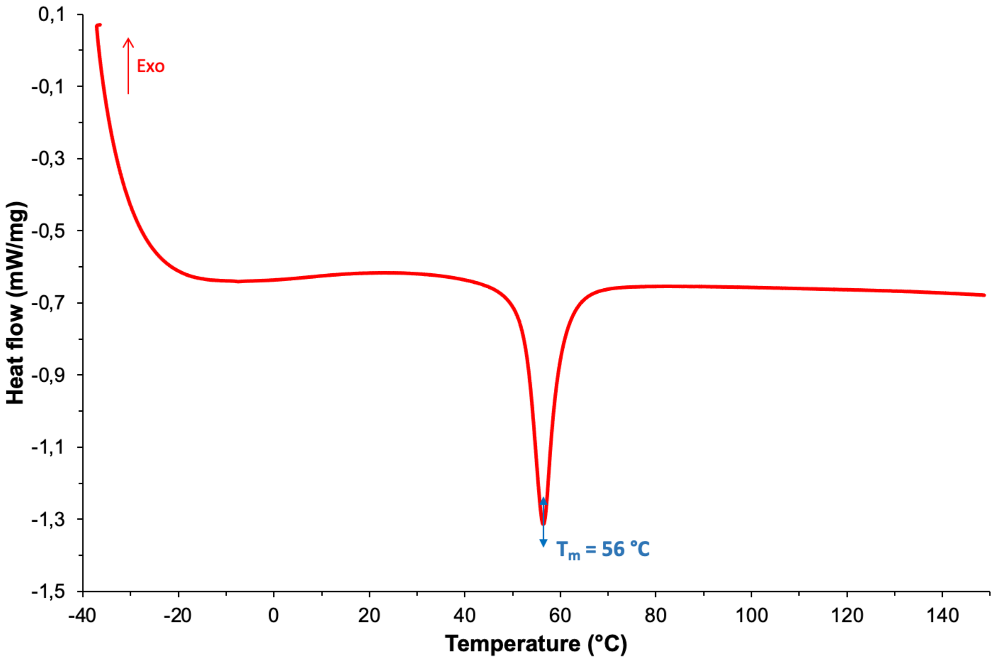

Figure S28. DSC measurement of P(FDA)SH.

S4.3 P(4VP-co-FDA)

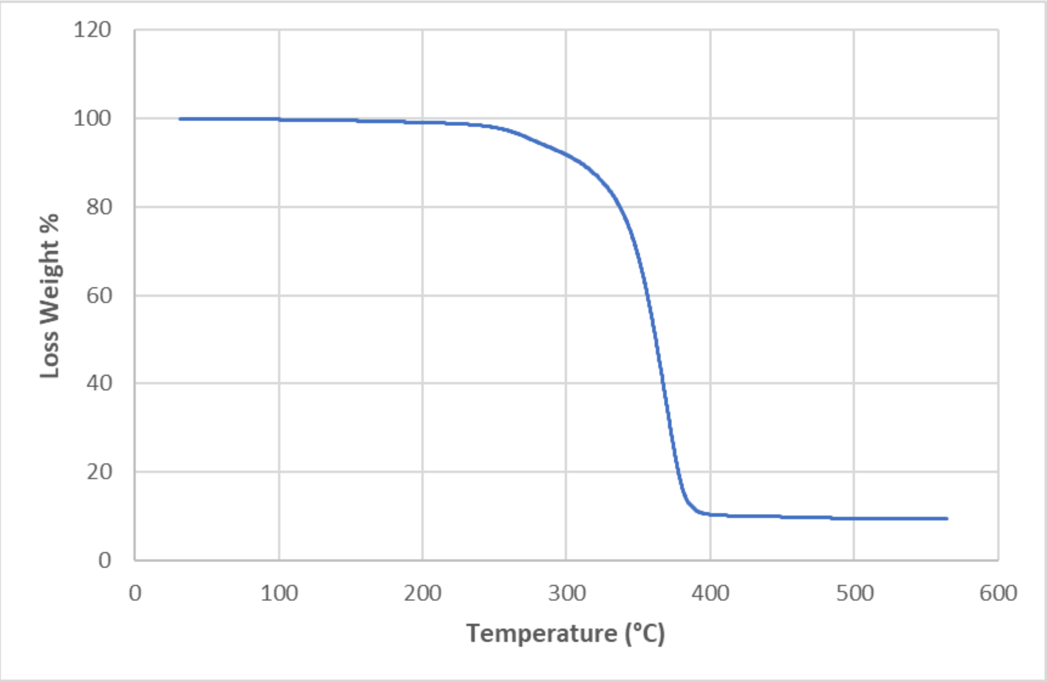

Figure S29. TGA measurement of P(4VP-*co*-FDA).

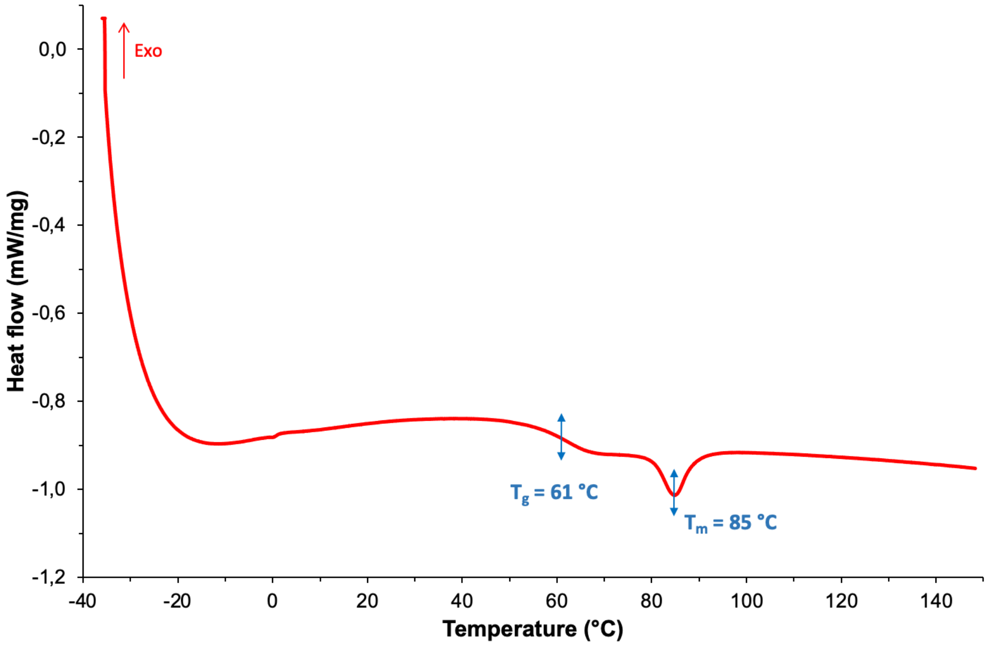

Figure S30. DSC measurement of P(4VP-*co*-FDA).

S4.4 P(4VP-co-FDA)SH

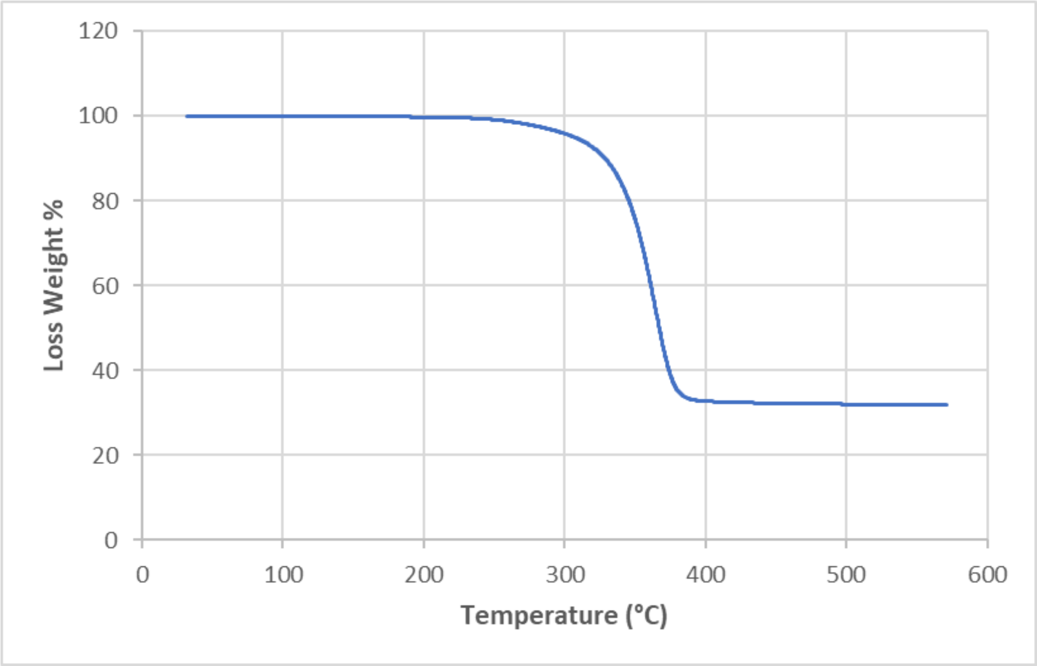

Figure S31. TGA measurement of P(4VP-*co*-FDA)SH.

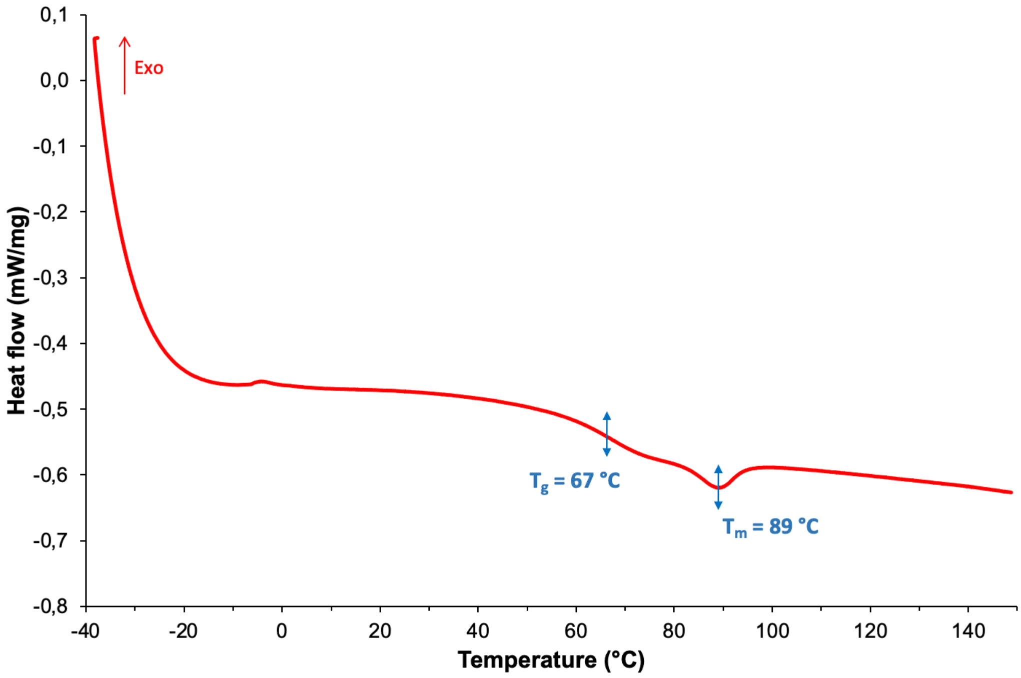

Figure S32. DSC measurement of P(4VP-*co*-FDA)SH.

S4.5 P(AAEM-co-FDA)

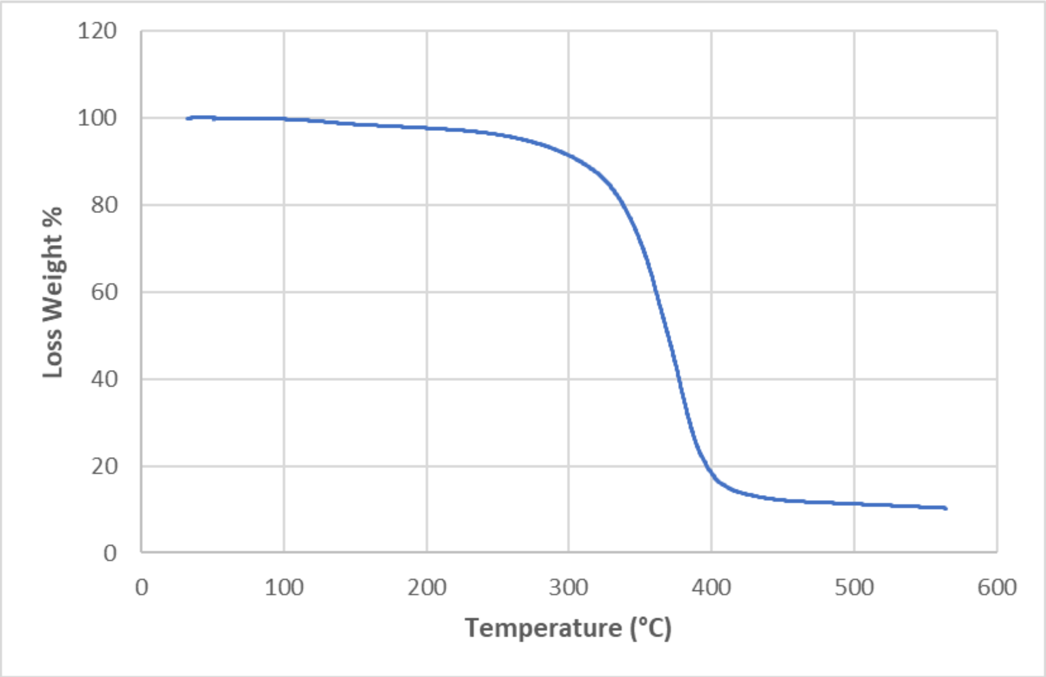

Figure S33. TGA measurement of P(AAEM-*co*-FDA).

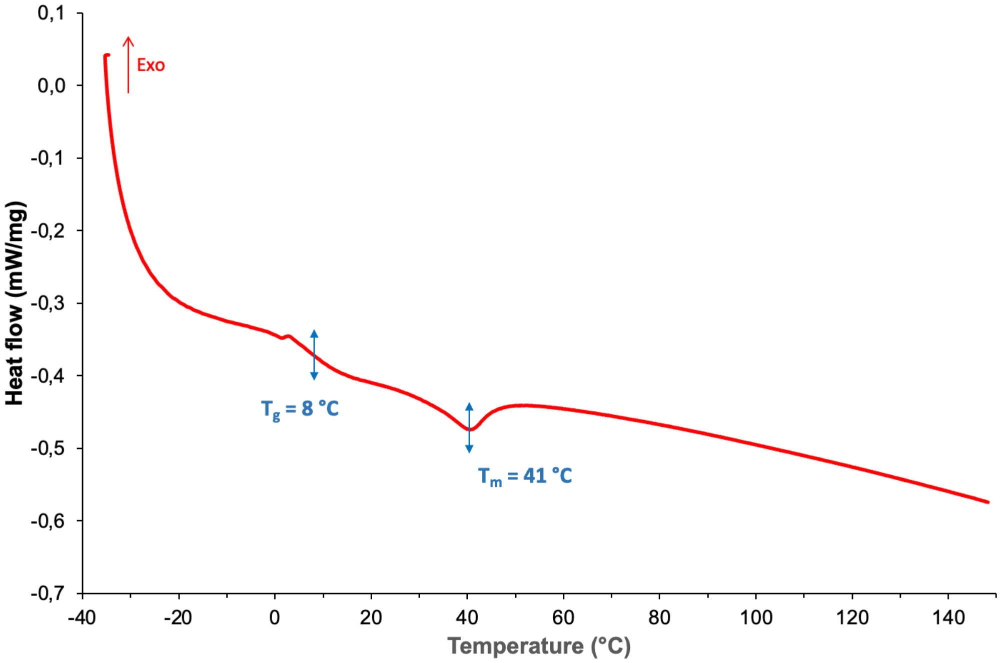

Figure S34. DSC measurement of P(AAEM-*co*-FDA).

S4.6 P(AAEM-co-FDA)SH

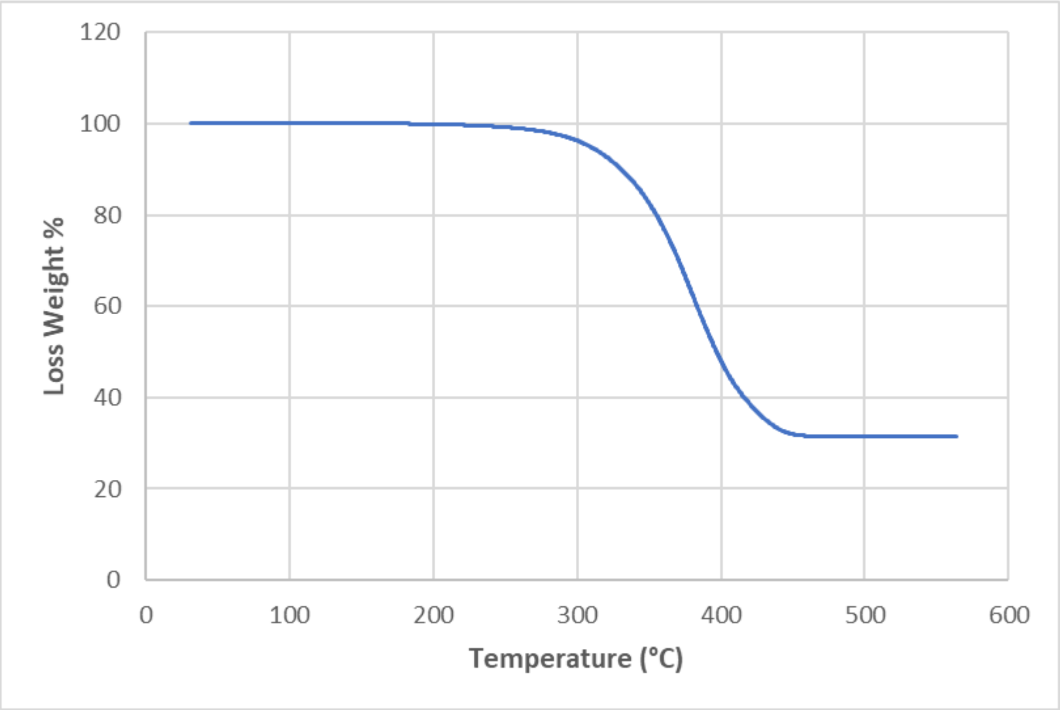

Figure S35. TGA measurement of P(AAEM-*co*-FDA)SH.

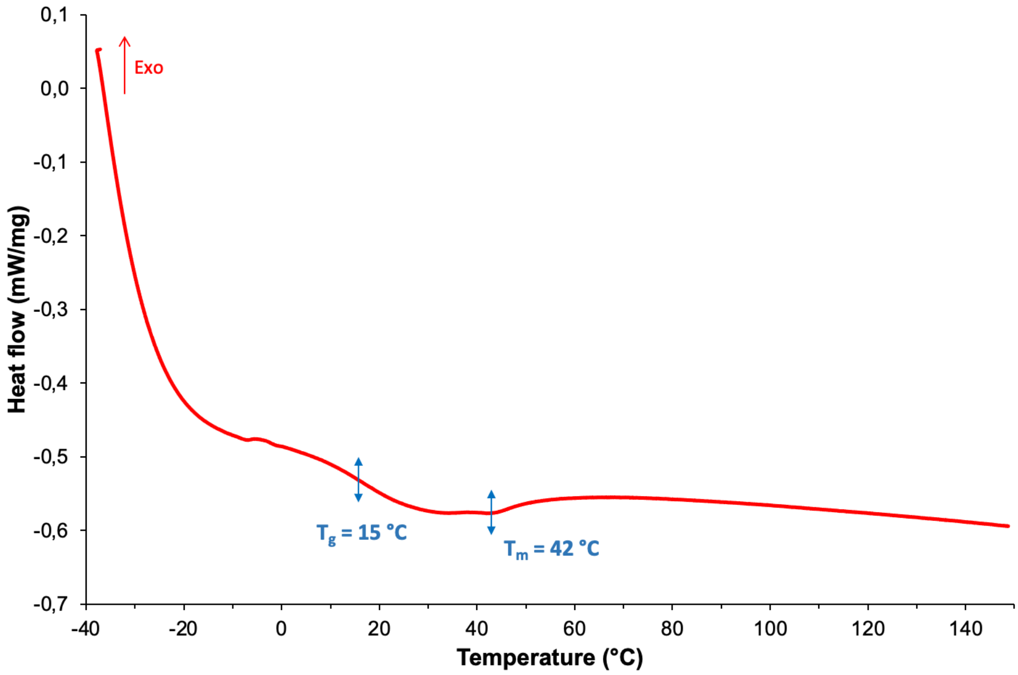

Figure S36. DSC measurement of P(AAEM-*co*-FDA)SH.

S4.7 P(DPPS-co-FDA)

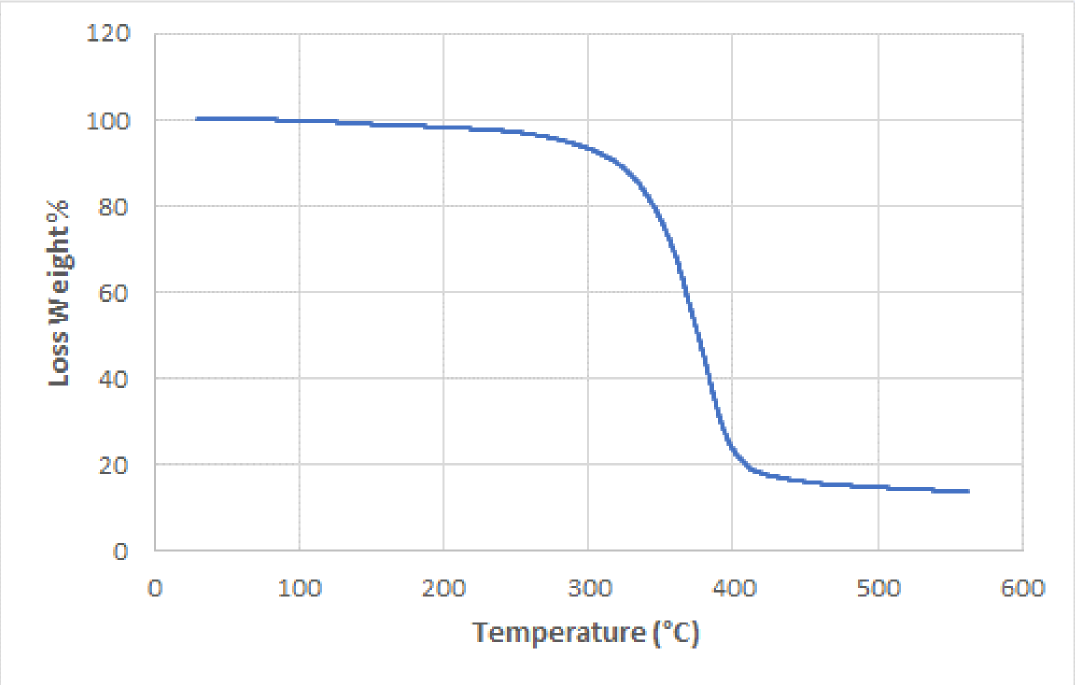

Figure S37. TGA measurement of P(DPPS-*co*-FDA).

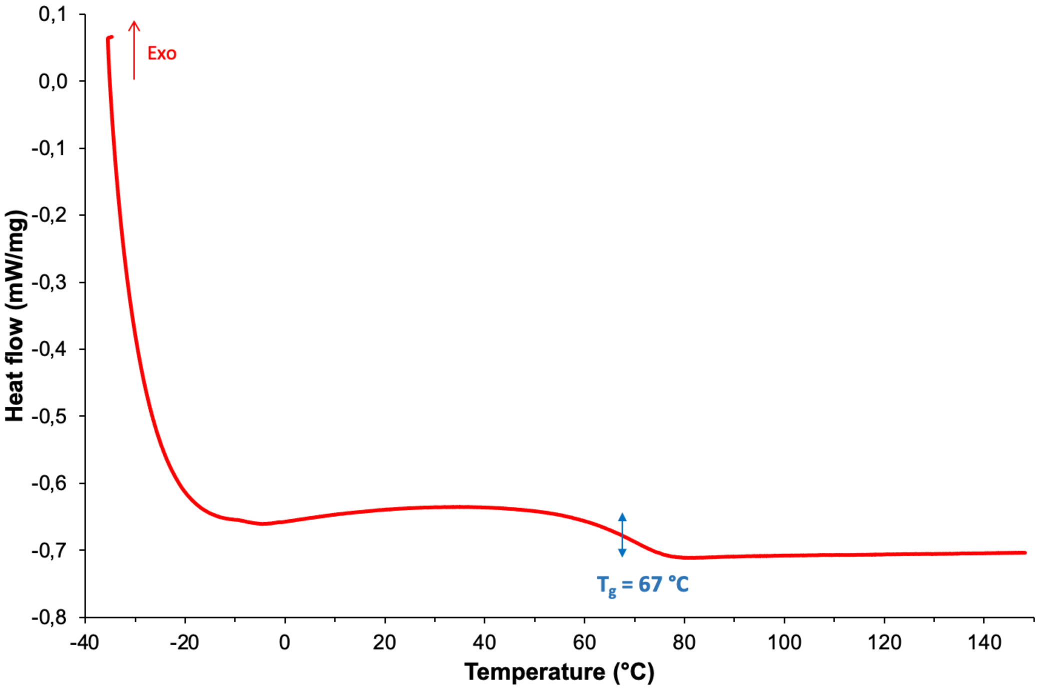

Figure S38. DSC measurement of P(DPPS-*co*-FDA).

S4.8 P(DPPS-co-FDA)SH

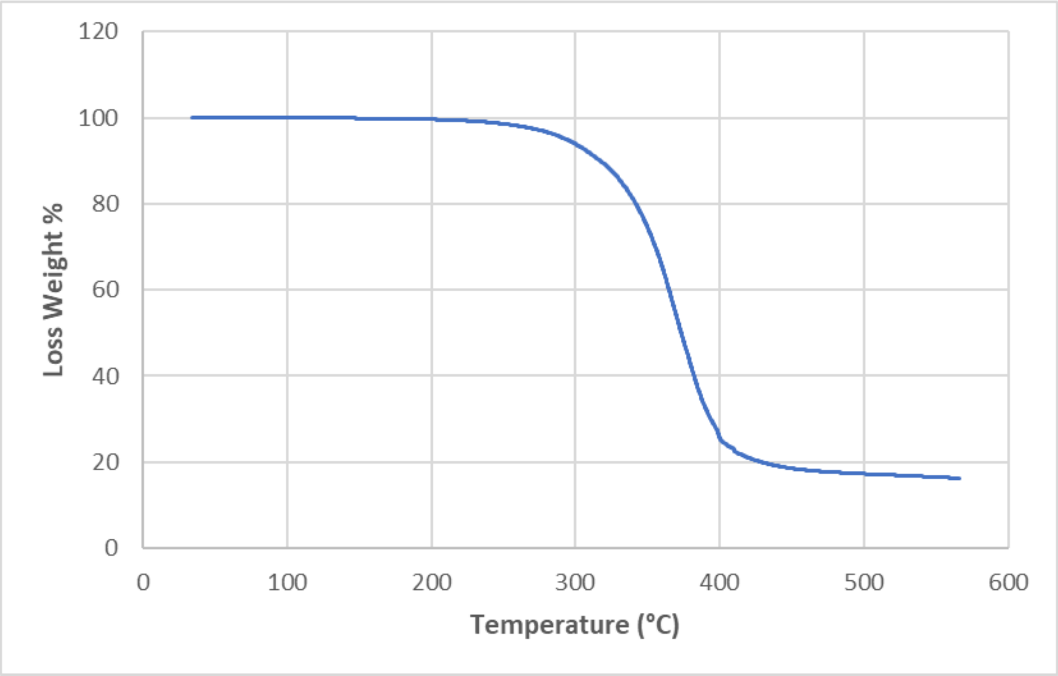

Figure S39. TGA measurement of P(DPPS-*co*-FDA)SH.

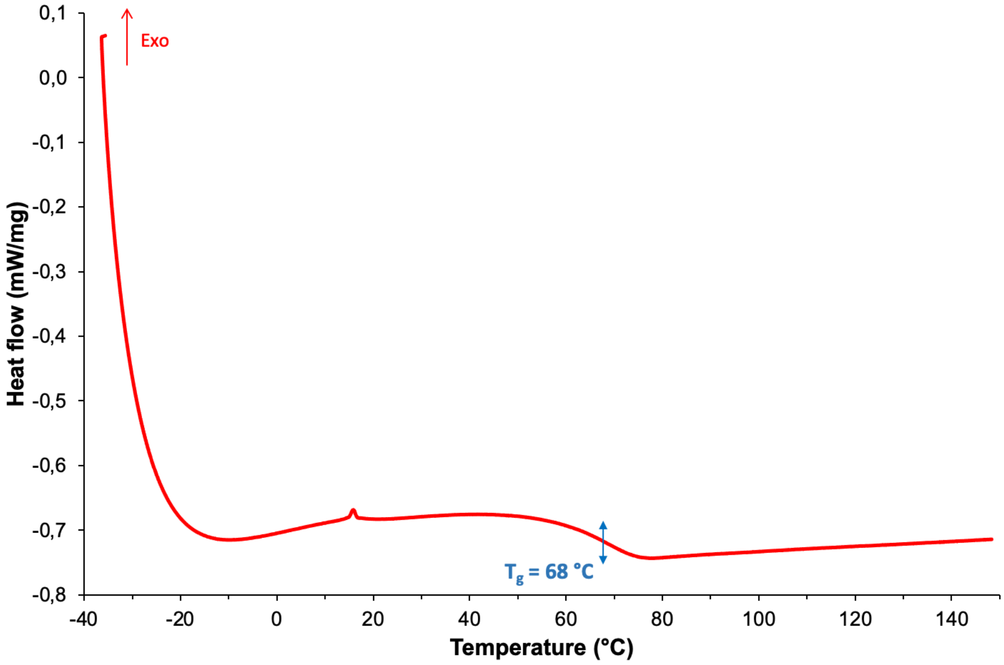

Figure S40. DSC measurement of P(DPPS-*co*-FDA)SH.

S4.9 P(StySAc-co-FDA)

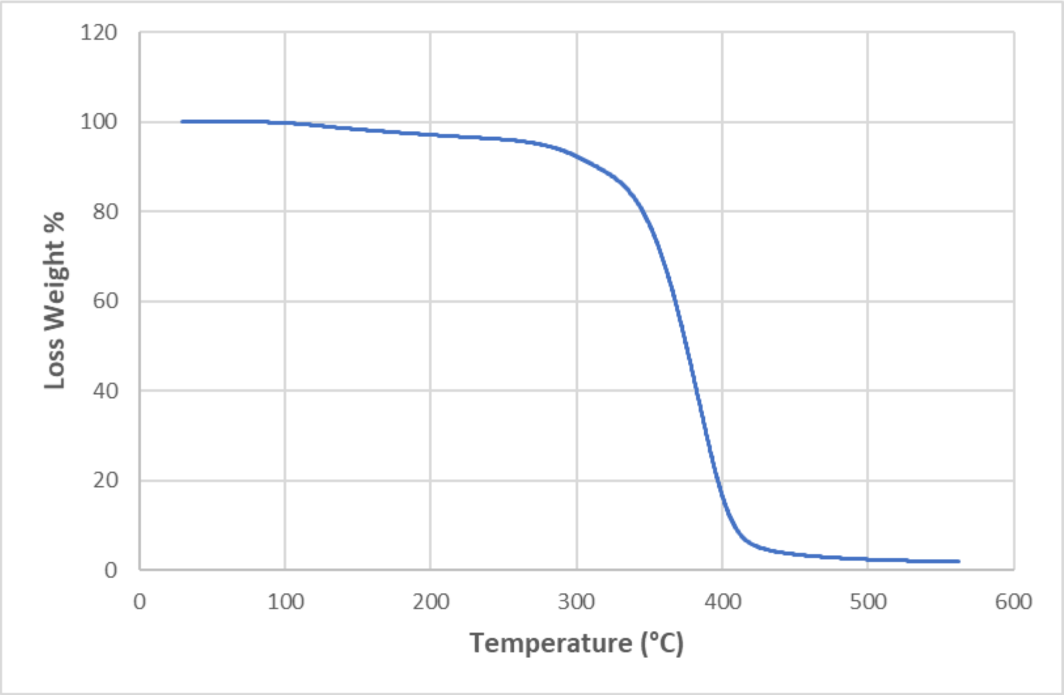

Figure S41. TGA measurement of P(StySAc-*co*-FDA).

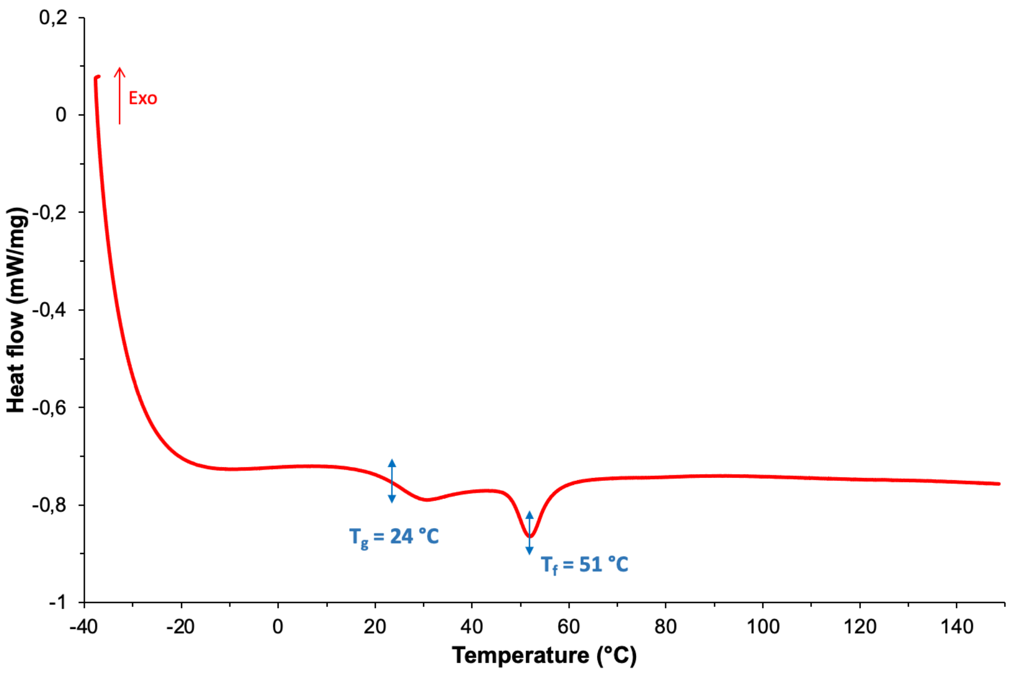

Figure S42. DSC measurement of P(StySAc-*co*-FDA).

S4.10 P(StySH-co-FDA)SH

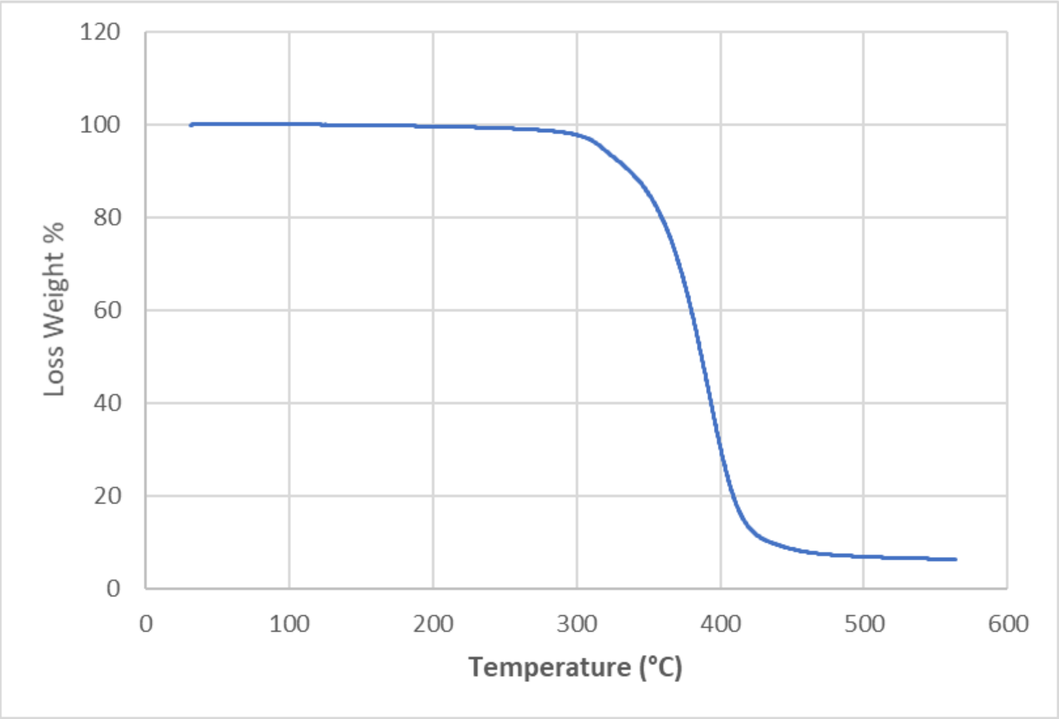

Figure S43. TGA measurement of P(StySH-*co*-FDA)SH.

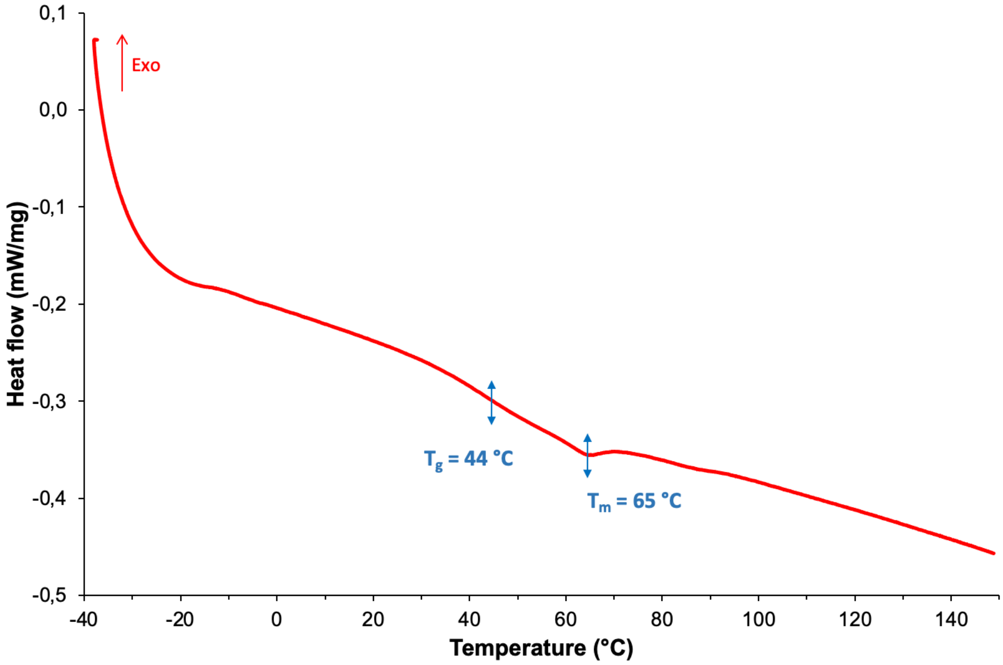

Figure S44. DSC measurement of P(StySH-*co*-FDA)SH.

S5. Cloud-Point measurements

S5.1 P(FDA)

polymer concentration = 0.01035 g_{pol}/g_{CO2}

T (°C)	P (MPa)
24.6	6.2
29.9	7.4
35.3	8.4
39.2	9.6
44.1	10.9
49	12.1
53.5	13.4
58	14.6
63.6	15.8

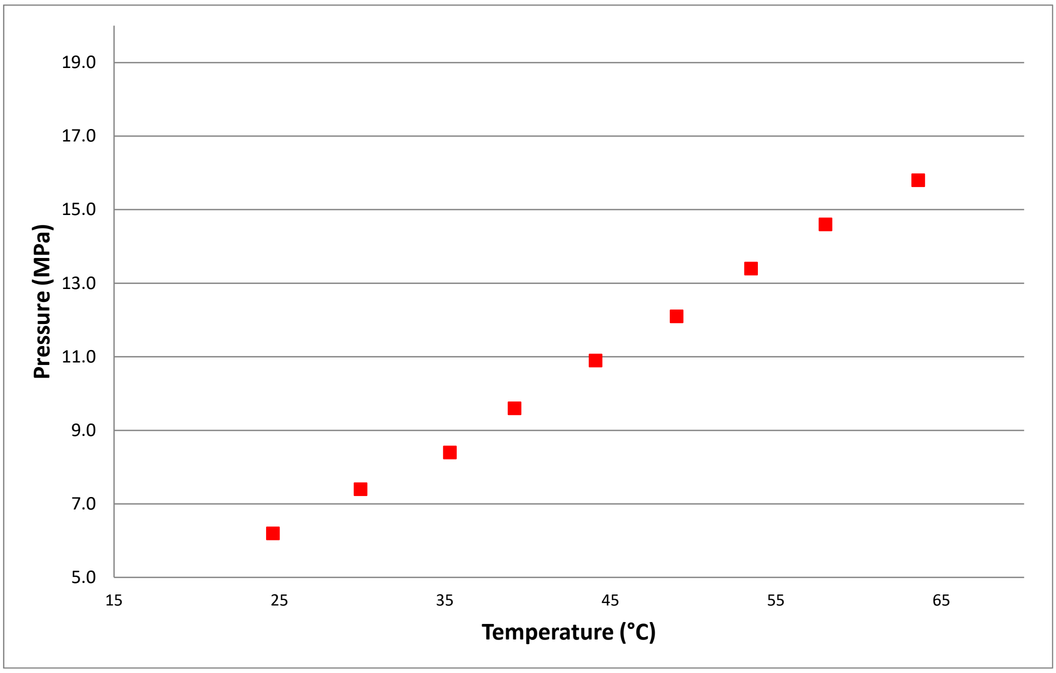

Figure S45. Cloud-Point measurement for P(FDA).

S5.2 P(FDA)SH

polymer concentration = 0.00954 g_{pol}/g_{CO2}

T (°C)	P (MPa)
25.8	6.3
30.6	7.8
36.2	9.3
40.4	10.6
44.5	11.7
49.2	13.1
53.6	14.2
58.4	16.0
64.3	19.0

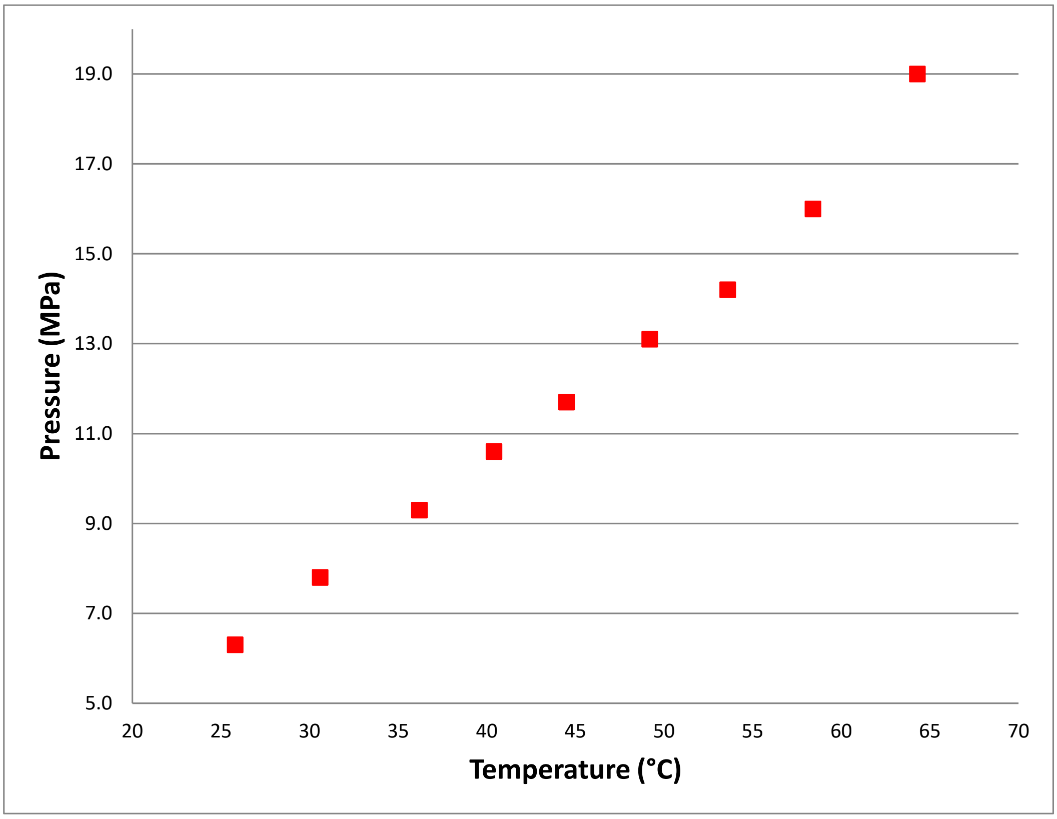

Figure S46. Cloud-Point measurement for P(FDA)SH.

S5.3 P(4VP-co-FDA)

polymer concentration = 0.01097 g_{pol}/g_{CO2}

T (°C)	P (MPa)
26.2	12.8
30.8	14.4
35.7	16.3
39.3	17.7
44.2	19.4
49.2	21.0
53.6	22.8
58.1	24.4
63.3	26.3

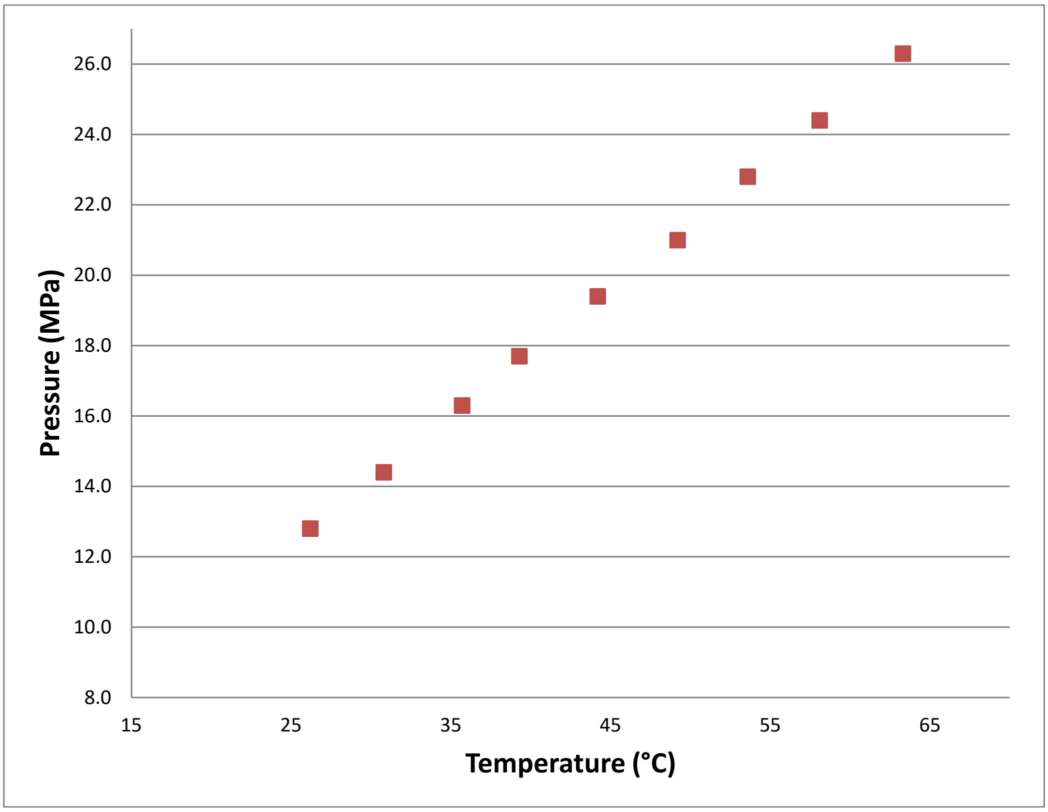

Figure S47. Cloud-Point measurement for P(4VP-*co*-FDA).

S5.4 P(4VP-co-FDA)SH

polymer concentration = 0.01089 g_{pol}/g_{CO2}

T (°C)	P (MPa)
24.9	12.2
30.3	13.8
34.1	15.4
39.1	17.1
43.4	18.7
48.3	20.5
53.1	22.5
59.4	24.8
64.1	26.8

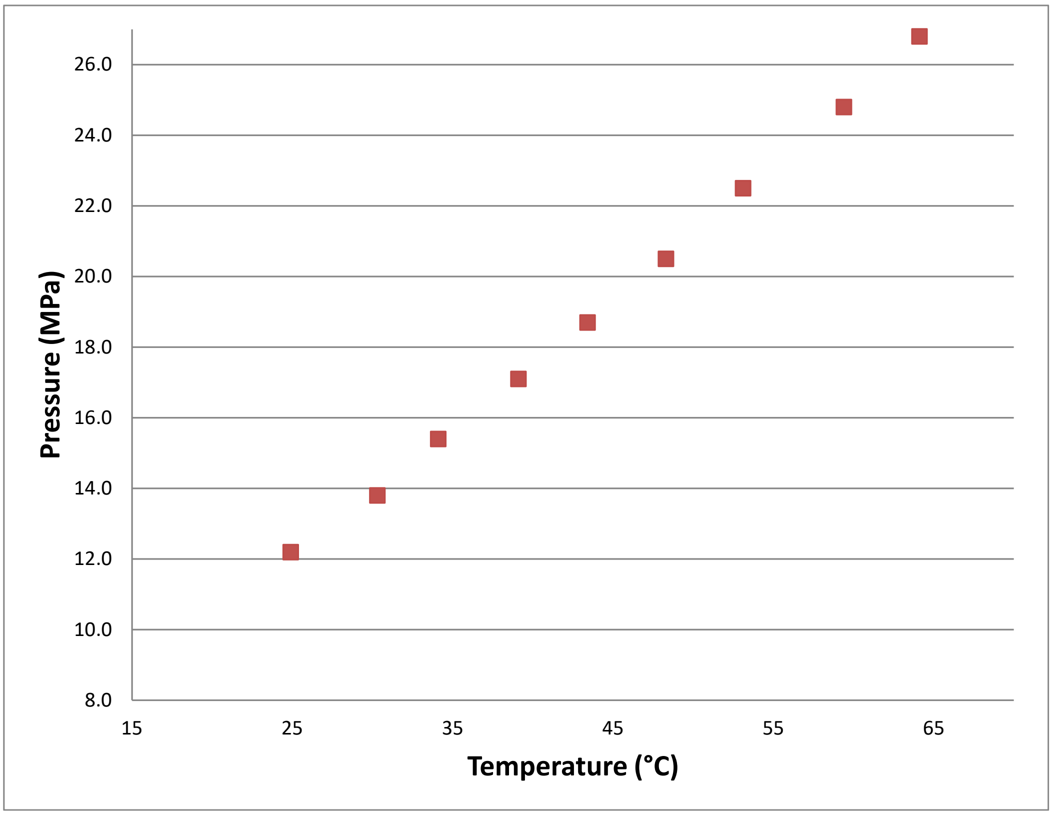

Figure S48. Cloud-Point measurement for P(4VP-*co*-FDA)SH.

S5.5 P(AAEM-co-FDA)

T (°C)	P (MPa)
25.2	8.0
30.6	10.1
34.8	12.1
39.5	14.0
45.2	16.2
49.4	18.0
54.4	19.9
59.2	21.7
64	23.3

polymer concentration = 0.01005 g_{pol}/g_{CO2}

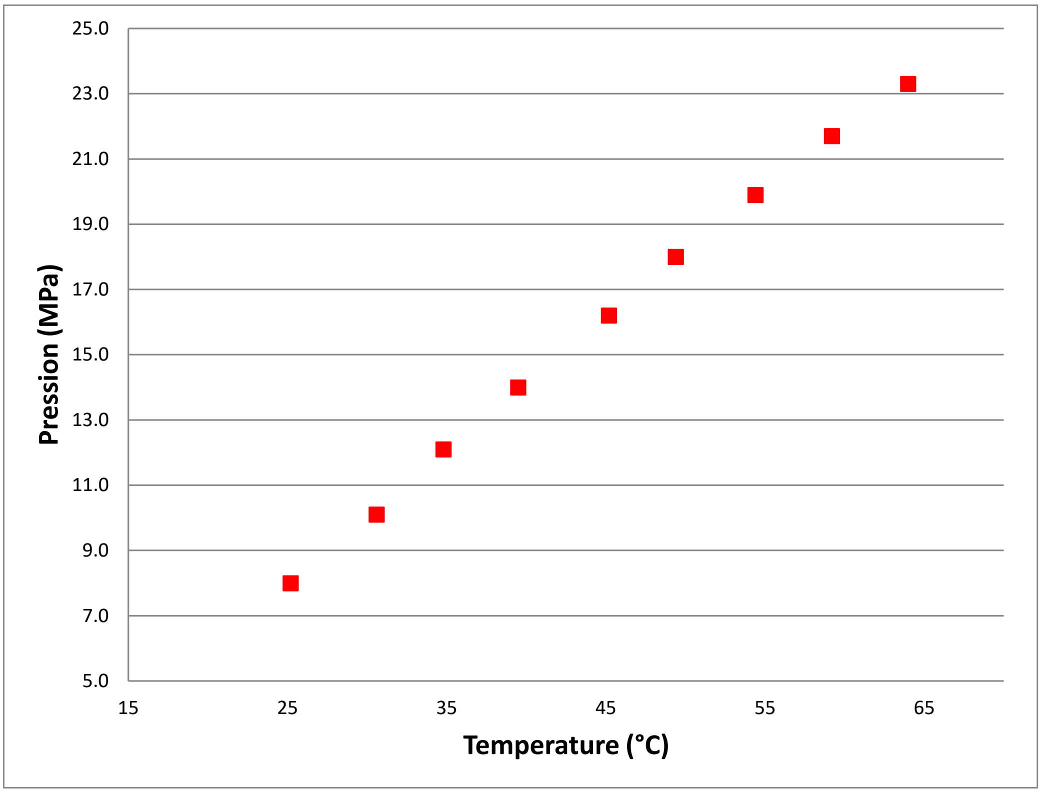

Figure S49. Cloud-Point measurement for P(AAEM-*co*-FDA).

S5.6 P(AAEM-co-FDA)SH

polymer concentration = 0.01124 g_{pol}/g_{CO2}

T (°C)	P (MPa)
26	8.1
30.9	10.2
35	12.2
40	14.6
44.2	16.6
48.8	18.9
53.4	21.1
58.8	23.9
63	26.5

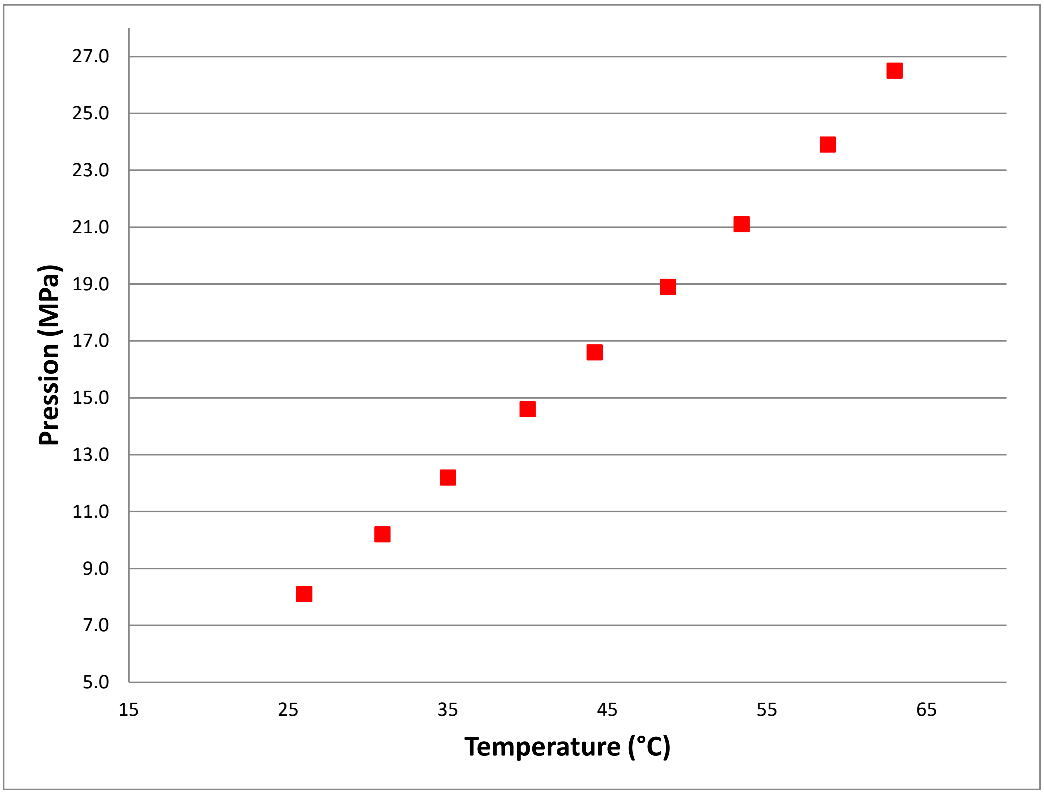

Figure S50. Cloud-Point measurement for P(AAEM-*co*-FDA)SH.

S5.7 P(DPPS-co-FDA)

T (°C)	P (MPa)
25.1	11.0
29.9	12.5
34.2	14.1
38.1	15.6
44.1	17.7
49.1	19.2
53	20.5
57.9	22.2
62.8	24.0

polymer concentration = 0.00849 g_{pol}/g_{CO2}

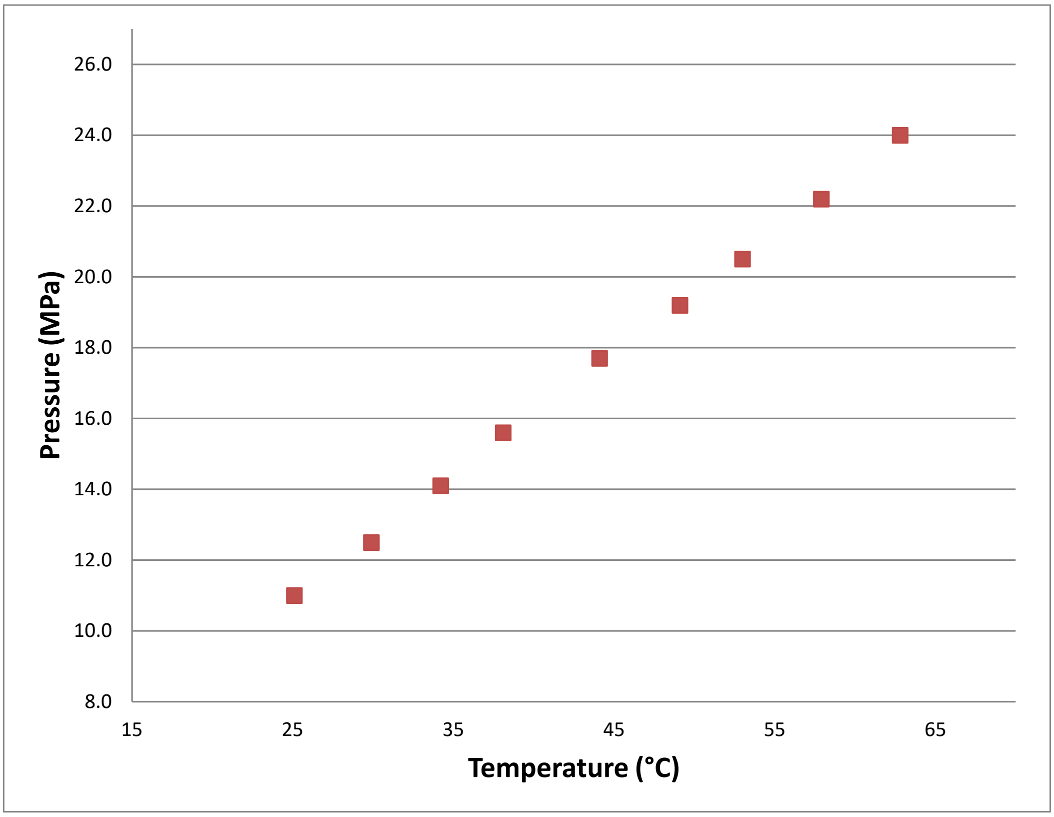

Figure S51. Cloud-Point measurement for P(DPPS-*co*-FDA).

S5.8 P(DPPS-co-FDA)SH

T (°C)	P (MPa)
25.2	11.8
30	13.6
35	15.8
39.5	18.1
45	20.1
50.2	21.4
55.1	22.9
59	24.3
64.5	26.2

polymer concentration = 0.00939 g_{pol}/g_{CO2}

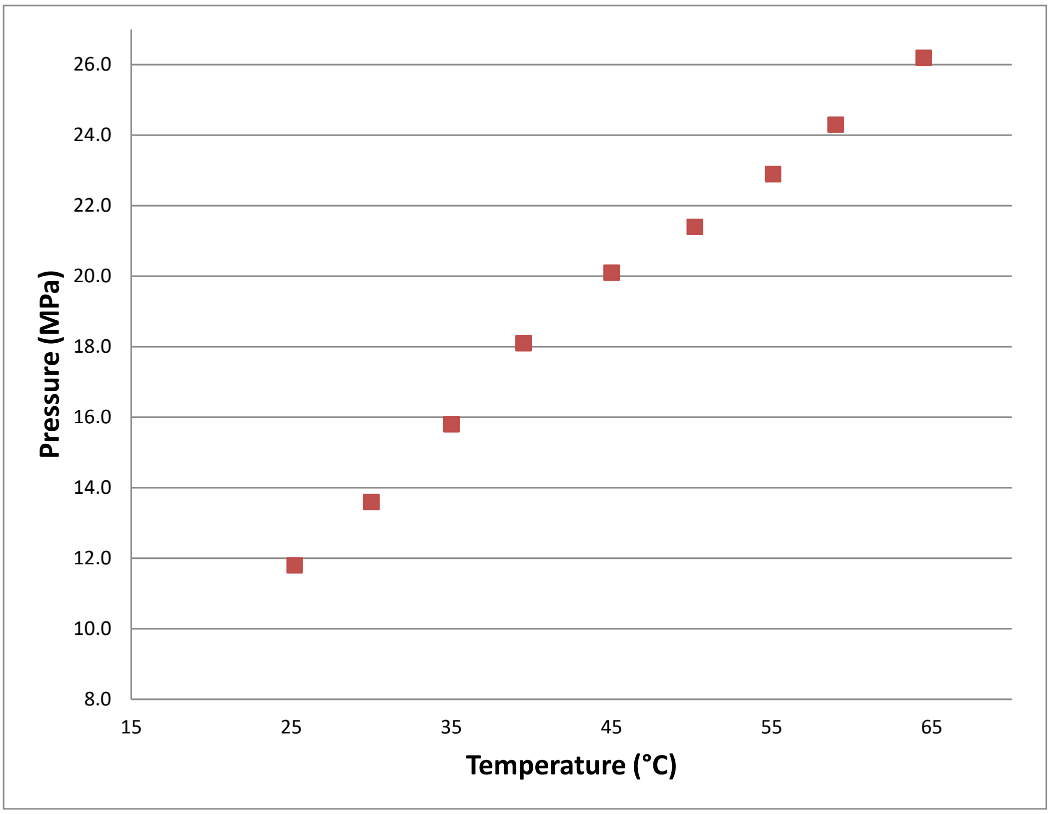

Figure S52. Cloud-Point measurement for P(DPPS-*co*-FDA)SH.

S5.9 P(StySAc-co-FDA)

T (°C)	P (MPa)
26.1	8.6
30.6	10.2
35	11.7
39.7	13.6
44.4	15.4
49.3	16.8
53.8	18.4
58.1	20.2
63.5	22.2

polymer concentration = 0.01044 g_{pol}/g_{CO2}

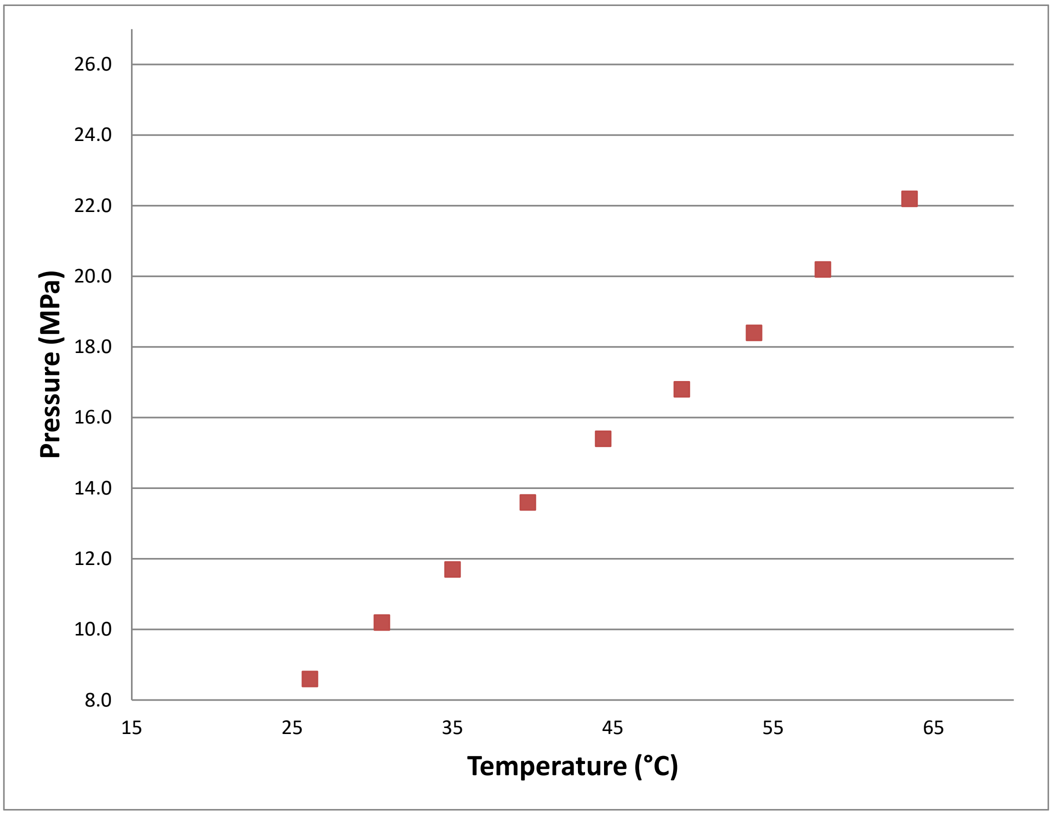

Figure S53. Cloud-Point measurement for P(StySAc-*co*-FDA).

S5.10 P(StySH-co-FDA)SH

polymer concentration = 0.01124 g_{pol}/g_{CO2}

T (°C)	P (MPa)
26	8.9
30.6	10.4
35.1	12.1
39.5	13.8
44.2	15.3
49.6	16.9
53.5	18.5
58.8	20.2
63.3	21.9

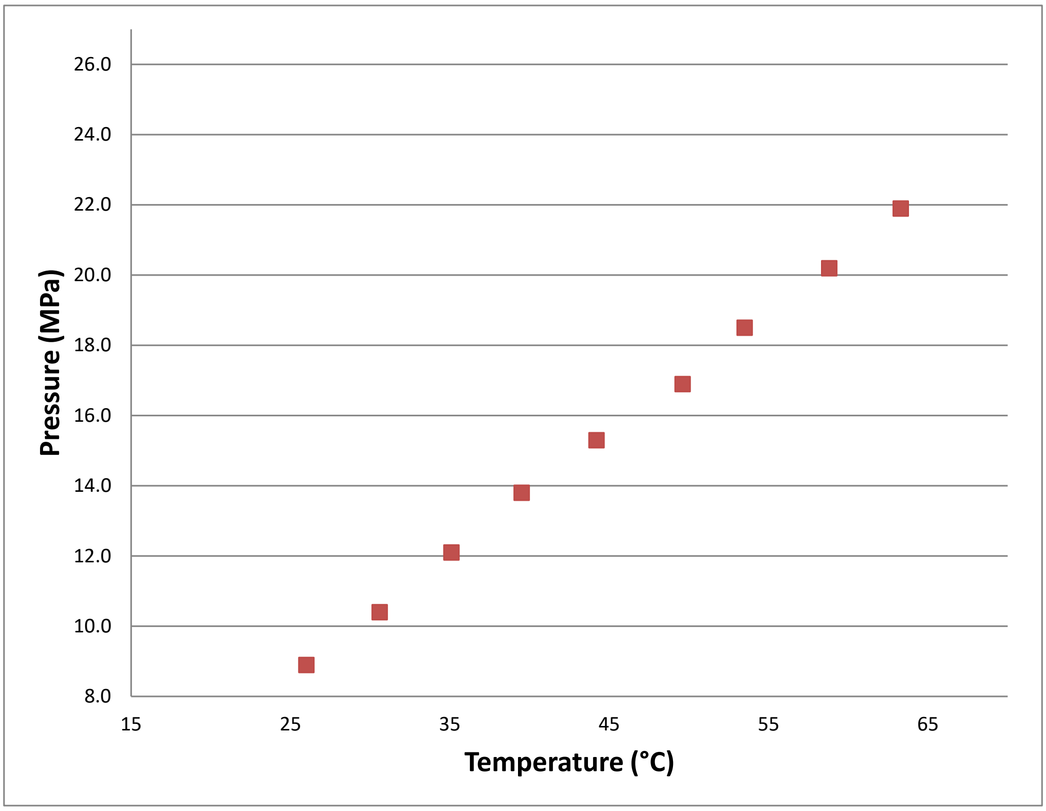

Figure S54. Cloud-Point measurement for P(StySH-*co*-FDA)SH.

S5.11 Overlay of the (co)polymer cloud points

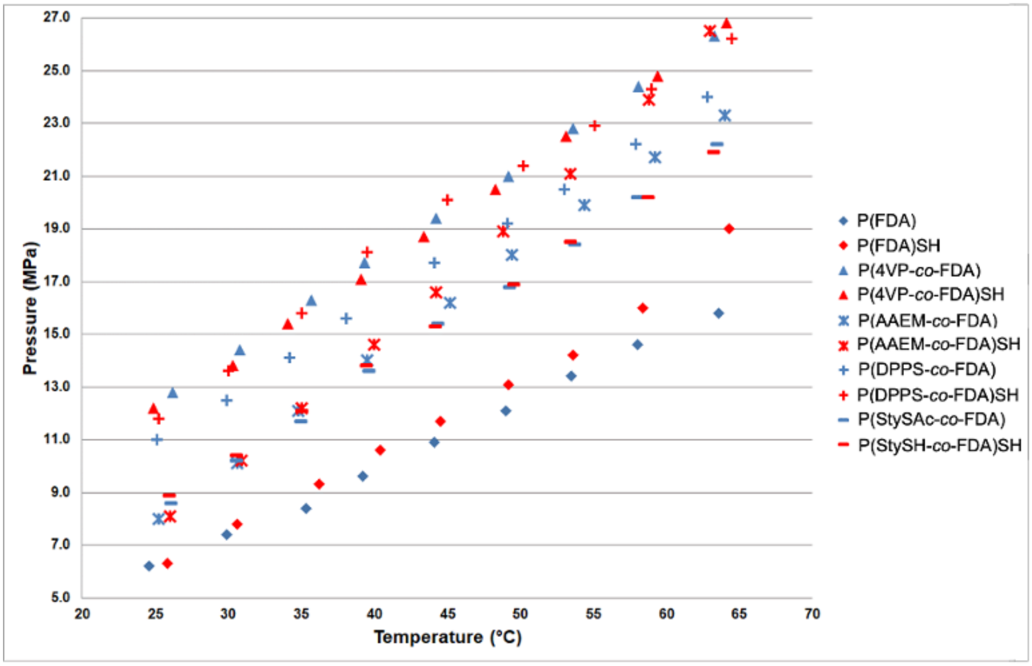

Figure S55. Cloud point curves in dense CO_2 for the synthesized polymers (at a polymer concentration of ca. 1 wt% of polymer relative to CO_2).
